# ﻿An updated world checklist of velvet worms (Onychophora) with notes on nomenclature and status of names

**DOI:** 10.3897/zookeys.1184.107286

**Published:** 2023-11-16

**Authors:** Ivo de Sena Oliveira

**Affiliations:** 1 Department of Zoology, Institute of Biology, University of Kassel, Heinrich-Plett-Straße 40, D-34132, Kassel, Germany University of Kassel Kassel Germany; 2 Departamento de Zoologia, Instituto de Ciências Biológicas, Universidade Federal de Minas Gerais, Av. Presidente Antônio Carlos 6627, 31270-901, Belo Horizonte, Minas Gerais, Brazil Universidade Federal de Minas Gerais Belo Horizonte Brazil

**Keywords:** Catalogue, extant, fossil, Peripatidae, Peripatopsidae, species list, taxonomy

## Abstract

More than a decade has passed since the publication of the only world checklist available for Onychophora. During this period, numerous nomenclatural acts and taxonomic changes have been suggested within the group and a wealth of novel data has been published on many taxa. Herein, the up-to-date taxonomic scenario within Onychophora is presented, with appraisal of name status. This checklist covers both extant (Peripatidae and Peripatopsidae) and fossil taxa, and each species is accompanied by information on synonyms, type designation, holotype location, type locality, and language of original description. Additional remarks include nomenclatural inconsistencies, synonymizations, name misspellings, conflicting collecting event data, availability of taxonomically informative molecular data, etc. According to the data, 237 species are currently assigned to Onychophora: 140 of Peripatopsidae, 92 of Peripatidae, and five fossil species with unclear relationship to extant taxa. Since the previous checklist, 37 species have been added to Onychophora, representing an increase of 18.5% in the diversity described for the group. Yet, taxonomic descriptions seem slow-paced, with an average of 3.6 onychophoran species being described annually. From the taxonomic standpoint, 216 species are valid, although many of them require morphological revision and molecular characterization; 21 species exhibit major taxonomic ambiguities and have been regarded as nomina dubia. Recurrent taxonomic issues identified in the literature include inaccurate collecting event data, doubtful taxonomic assignment of molecular sequences, and non-observance of nomenclatural rules. These and other taxonomic aspects are addressed herein in the light of the directives established by the International Code of Zoological Nomenclature.

## ﻿Introduction

Onychophorans, or velvet worms, have long attracted the attention of scientists due to their rarity, peculiar lifestyle, and significance for studies of animal diversity, evolution, biogeography, and conservation. These organisms were discovered nearly two centuries ago (Guilding 1826) but many aspects of their biology are still little understood. For example, the actual number and validity status of species assigned to Onychophora has only been clarified recently, when the first and only world checklist available for this group was published (Oliveira et al. 2012a). This study showed that until early 2012, a total of 197 recent species had formally been described and assigned to two major subgroups: the Peripatidae, holding 82 species, and the Peripatopsidae, with 115 species; unambiguous fossils with uncertain relationship to the extant taxa included three additional species (Oliveira et al. 2012a). Most importantly, Oliveira et al. (2012a) exposed the fact that nearly all peripatids and several peripatopsids required revision, and at least 20 species must be regarded as nomina dubia due to major taxonomic inconsistencies.

The onychophoran checklist represented a milestone in the studies of the group and, even though a decade has passed since its publication, the list still stands as one of the primary sources of taxonomic information on Onychophora for many researchers and databases worldwide. However, numerous nomenclatural changes have occurred within Onychophora in the last years: several species have been described (e.g., Oliveira et al. 2012b, 2013, 2015, 2018; Ruhberg and Daniels 2013; Daniels et al. 2013, 2016; Oliveira and Mayer 2017; Costa et al. 2018; Sato et al. 2018; Barquero-González et al. 2020; Barnes et al. 2020; Costa and Giribet 2021; Barnes and Daniels 2022), different taxa have been revised in the light of novel morphological and molecular data (e.g., Chagas-Júnior and Costa 2014; Daniels et al. 2016; Oliveira et al. 2018; Costa et al. 2018; Barnes and Daniels 2022), and fossil species have been revisited and resolved within either the stem- or crown-group Onychophora (e.g., Oliveira et al. 2016; Murdock et al. 2016; Garwood et al. 2016). Furthermore, relevant historical information has belatedly been uncovered in old literature and numerous amendments have kindly been suggested by international colleagues over the years. Therefore, updating the world checklist of Onychophora seemed timely.

Herein, relevant taxonomic data collected during the past decade have been combined with those of Oliveira et al. (2012a) into a more comprehensive and up-to-date version of the annotated checklist of Onychophora. The new checklist follows the same layout of Oliveira et al. (2012a), but its content has completely been revisited to better meet the current standards of onychophorology. Previous data on synonyms, type designation, location of the holotype, type locality, and language of original description have been updated, as well as the remarks on nomenclatural inconsistencies, synonymizations, misspellings, etc. In addition, comments on conflicting collecting event data, possible species complexes, and availability of molecular sequences useful for taxonomy have been added to the revised checklist. The updated information was then used to re-assess species names status within Onychophora in the light of the International Code of Zoological Nomenclature. Therefore, the revised world checklist of Onychophora may constitute a valuable reference material for researchers interested in velvet worms, in addition to creating a basis for future taxonomical works on the group.

## ﻿Materials and methods

The present checklist has been compiled using its previous version (Oliveira et al. 2012a) as template. Taxonomic data and nomenclatural acts published after Oliveira et al. (2012a), as well as missing data found in old literature and amendments suggested by international colleagues, have been collected over the years and incorporated into the revised list. As in the previous version, the rules of the International Code of Zoological Nomenclature (ICZN 1999) have been applied thoughtfully to assess the validity status of each species name, address nomenclatural inconsistencies, and evaluate ambiguous synonymizations.

Onychophoran species typically exhibit high levels of endemism and very limited distribution ranges, with cryptic speciation being a recurrent phenomenon among species believed to be widely distributed (Lacorte et al. 2011a, b; Oliveira et al. 2011, 2018; Daniels et al. 2016; Costa et al. 2018; Barnes and Daniels 2019, 2022; Barnes et al. 2020). With that in mind, collecting data and geographic coordinates provided in original species descriptions have been verified and the distance between the type locality and additional collecting sites recorded for each species has been measured using the freeware Google Earth Pro. The obtained data have been used for inferring putative species complexes and evaluating synonymizations, assuming that conspecificity becomes unlikely when specimens have been collected ≥ 30 km away from each other (see Oliveira et al. 2011: 2, 2012a: 3).

The revised checklist is structured in three subsections corresponding to the two major subgroups of living onychophorans, Peripatidae and Peripatopsidae, and to fossil species with uncertain relationship to the extant taxa. Within each section, genera and valid species names are sorted alphabetically, and numbered consecutively, with Roman and Arabic numerals, respectively. Nomina dubia are presented separately within the corresponding taxon but numbered consecutively throughout each subsection using the initials nd followed by Arabic numerals (e.g., nd1, nd2, nd3…). Fossil taxa are identified by a dagger (†). The synonym list presented for each species is sorted chronologically and only includes the first mention to a given synonym; except for diacritical and other marks, misspellings are not regarded as synonyms, but rather mentioned in the Remarks section of each species. For the sake of clarity, abbreviations are intentionally avoided throughout the entire checklist.

**Figure 1.**
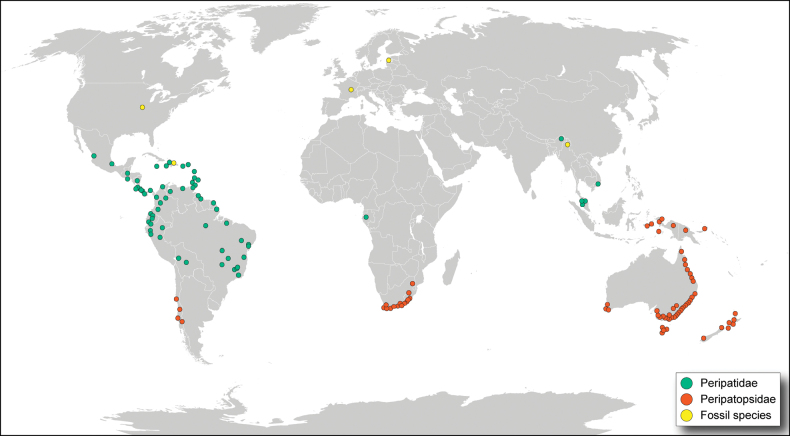
Word map showing the geographical distribution of valid onychophoran species. Dots represent the type locality of one or more species of Peripatidae [in green] and Peripatopsidae [in orange]; yellow dots represent the approximate position of fossiliferous deposits, from which onychophorans have been reported. Nomina dubia are not included in the map.

Data on type designation are restricted to name-bearing types that consist of a single trackable specimen, i.e., holotypes, neotypes, and lectotype. Since syntype series may include specimens from different localities, the conspecificity of which has not been assessed herein, information on syntypes is only provided in the Remarks section. Paratypes are not considered in the present checklist, as they do not represent name-bearing types (ICZN 73.1). Only type locality data are presented for each species, instead of putative geographical ranges; in case of syntypes obtained from different localities, the site where most specimens have been collected has been regarded as the species type locality. Locality data are given in the following format: country name, subordinated administrative divisions from broader to narrower, locality name, geographic coordinates, altitude. When necessary, locality names have been updated and old names have been provided alongside current ones. Geographic coordinates (if available) are presented in DDM form (Degrees, Decimal Minutes); coordinates published in different formats have herein been converted to this system. Missing altitude data has been retrieved using the freeware Google Earth Pro based on geographic coordinates provided in the original literature. The International System of Units (SI) (NIST, 2019) is used throughout the list; information published in different units are presented along with its converted values (herein 1 ft = 0.3048 m; 1 mile = 1.6 km). All available data on the type localities of valid species (Fig. 1) are included into a world map based on the information obtained from Google Earth.

## ﻿Checklist

### ﻿Onychophora Grube, 1850

**Remarks.** The name Onychophora has commonly been assigned to Grube (1853: 351), although in fact, the same author had already suggested the term three years earlier (Grube 1850: 275). Hence, the correct citation of the name is ‘Onychophora Grube, 1850’.

#### ﻿Peripatidae Audouin & Milne-Edwards, 1832

**Type genus.***Peripatus* Guilding, 1826.

**Remarks.** Although the name Peripatidae has long been credited to Evans (1901a: 480), the term ‘Péripatiens’ deemed as a family had been introduced several decades earlier by Audouin and Milne-Edwards (1832: 384). The earliest record of the spelling “Peripatidae” is found in Burmeister (1837: 541). Since earlier reference to this name has not yet been found in the literature, its correct citation is proposed as ‘Peripatidae Audouin & Milne-Edwards, 1832’. Peripatidae is arguably as the least studied subgroup of Onychophora, although the number of publications on this clade has increased substantially in recent years. A thorough revision is required, particularly of neotropical taxa (Oliveira et al. 2012a: 4).

##### I. *Cerradopatus* Oliveira, Lacorte, Weck-Heimann, Cordeiro, Wieloch & Mayer, 2015

**Type species.***Cerradopatussucuriuensis* Oliveira, Lacorte, Weck-Heimann, Cordeiro, Wieloch & Mayer, 2015, by monotypy (Oliveira et al. 2015: 216).

**Remarks.***Cerradopatus* has recently been regarded as junior synonym of *Epiperipatus* Clark, 1913a (Costa et al. 2021). However, the classification of *Epiperipatus* is still inconclusive (see Remarks for *Epiperipatus* below). For the sake of nomenclatural stability, and following the ICZN (Art. 23.3.6), *Cerradopatus* is considered herein as the valid name; *Epiperipatus*, as defined by Costa et al. (2021) is referred to as sensu lato.

**1. *Cerradopatussucuriuensis* Oliveira, Lacorte, Weck-Heimann, Cordeiro, Wieloch & Mayer, 2015**

**Synonyms.***Epiperipatussucuriuensis* (Costa et al. 2021: 790) (see Remarks).

**Holotype.** Deposited in the Zoology Department of the Instituto de Ciências Biológicas, Universidade Federal de Minas Gerais, Belo Horizonte, Brazil.

**Type locality.** Brazil, Mato Grosso do Sul, Chapadão do Sul, environs of the PCH [= small hydroelectric power plant] Porto das Pedras at the river Sucuriú, 52°32.55'W, 19°28.73'S, ca 420 m above sea level (= ASL).

**Language of species description.** English.

**Remarks.** Species recently classified within *Epiperipatus* sensu lato by Costa et al. (2021) but treated herein under its original combination following the ICZN (Art. 23.3.6); further details provided in the Remarks for *Epiperipatus* below. Species description includes morphological, molecular, karyotype, and slime protein profiling data (Oliveira et al. 2015). Slime protein profiling data first provided by Baer et al. (2014) under the name *Epiperipatus* sp.

##### II. *Eoperipatus* Evans, 1901a

**Type species.***Eoperipatushorsti* (Evans, 1901a), by subsequent designation (Oliveira et al. 2012a: 4) (see Remarks).

**Remarks.***Eoperipatushorsti* has been designated as the type species of *Eoperipatus* by Oliveira et al. (2012a: 4) following recommendations of the ICZN (Art. 67) and based on the large amount of information available for this species in the literature. Also, *Eoperipatushorsti* is the only originally included nominal species, for which data from both sexes are available. An emended diagnosis has recently been provided for this genus (Oliveira et al. 2012b: 18).

**2. *Eoperipatusbutleri* Evans, 1901b**

**Synonyms.***Eoperipatusweldoni* (Bouvier 1905: 358) (see Remarks).

**Holotype.** Deposited in the Natural History Museum of London, UK (see Remarks).

**Type locality.** Malaysia, Perak, Bukit Larut [= Larut Hills], ca 1,219 m [4,000 ft] ASL.

**Language of species description.** English.

**Remarks.***Eoperipatusbutleri* has previously been regarded as a synonym of *Eoperipatusweldoni* (Bouvier 1905: 358). However, the great distance between the type localities of these two species (more than 300 km apart) together with their morphological differences (Evans 1901b, a), led Oliveira et al. (2012a: 5) to regard *Eoperipatusbutleri* and *Eoperipatusweldoni* as separate species. The holotype has not been designated explicitly in the original description. Since *Eoperipatusbutleri* has been described based on a single specimen, the ‘syntype’ deposited in the Natural History Museum of London, UK, should be regarded as holotype fixed by monotypy, following the ICZN (Art. 73.1.2). Requires morphological revision and molecular characterization.

**3. *Eoperipatushorsti* Evans, 1901a**

**Synonyms.***Eoperipatussumatranus* (van der Lande and Holthuis 1986: 18) (see Remarks).

**Holotype.** Not designated (see Remarks).

**Type locality.** Malaysia, Kelantan, Kuala Aring.

**Language of species description.** English.

**Remarks.** Additional historical and morphological details of this species have been published after the original description (Bouvier 1905: 369). The holotype has not been designated explicitly in the original description. Bouvier (1907a: 519) refers to a cotype [obsolete for syntype] deposited in the Natural History Museum of London, UK. According to Kloss (1926: 167), *Eoperipatushorsti* is a lowland species, in contrast to the two other Malaysian species of *Eoperipatus*, namely *Eoperipatusbutleri* and *Eoperipatusweldoni*. Van der Lande and Holthuis (1986: 18) considered *Eoperipatushorsti* as a variety of *Eoperipatussumatranus*, a name which has later been used for all the Malaysian species (van der Lande 1988: 13; see Remarks for *Eoperipatussumatranus* below). Oliveira et al. (2012a: 6) regarded *Eoperipatushorsti* as a valid species, while *Eoperipatussumatranus* is as a nomen dubium. Requires morphological revision and molecular characterization.

**4. *Eoperipatustotoro* Oliveira, Schaffer, Kvartalnov, Galoyan, Palko, Weck-Heimann, Geissler, Ruhberg & Mayer, 2013**

**Synonyms.** None.

**Holotype.** Deposited in the Museum für Naturkunde Berlin, Germany.

**Type locality.** Vietnam, Dong Nai, Cát Tiên National Park, 107°20.73'E, 11°27.55'N, ca 120 m ASL.

**Language of species description.** English.

**Remarks.***Eoperipatustotoro* is described based on molecular and morphological data (Oliveira et al. 2013).

**5. *Eoperipatusweldoni* Evans, 1901a**

**Synonyms.***Eoperipatussumatranus* (van der Lande and Holthuis 1986: 18) (see Remarks).

**Holotype.** Not designated (see Remarks).

**Type locality.** Malaysia, possibly the Bukit Perangin Forest Reserve, northern Malaysia, next to the border to the southern part of Thailand (see Remarks). Originally described as “Bukit Besar, on the boundary line between the states of Nawng Chick and Jalor, a full day’s journey from the town of Patani ca 685 m [2,250 ft]” (Evans 1901a: 475, 486).

**Language of species description.** English.

**Remarks.** Additional historical and morphological details of this species have been published after the original description (Bouvier 1905: 357). The holotype has not been designated explicitly in the original description. Syntypes are deposited in the Natural History Museum of London, UK. Label data accompanying the syntypes only indicate ‘Bukit Besar, Malacca’ as type locality. According to Oliveira et al. (2012a: 6), however, the name Bukit Besar is attributed to different localities in Malaysia and the putative type locality of *Eoperipatusweldoni* may not lie in the Malacca State, as suggested by the label, but within the limits of the Bukit Perangin Forest Reserve (Kedah State). An area referred to as Bukit Besar is located in the western part of the Perangin Forest Reserve and matches both the altitude (690 m) and distance from Patani [sic] (104 km from Pattani, South Thailand) described by Evans (1901a: 475). Also, the Bukit Perangin Forest Reserve is located close to the border of the province Yala, also known as Jala or Jolor, indicating that the author might have misspelt it as Jalor [sic] (Oliveira et al. 2012a: 6). No region named Nawng Chick State could be identified in Malaysia. Van der Lande and Holthuis (1986: 18) considered *Eoperipatusweldoni* as a variety of *Eoperipatussumatranus*, a name which was later used for all the Malaysian species (van der Lande 1988: 13; see Remarks for *Eoperipatussumatranus* below). Oliveira et al. (2012a: 6) regarded *Eoperipatusweldoni* as a valid species, while *Eoperipatussumatranus* is as a nomen dubium. Requires morphological revision and molecular characterization.

**Nomen dubium**

**nd1. *Eoperipatussumatranus* (Sedgwick, 1888)**

**Synonyms.***Peripatussumatranus*, as originally described (Sedgwick 1888: 485); *Eoperipatussumatranus* (Evans 1901a: 484).

**Holotype.** Not designated (see Remarks).

**Type locality.** Unknown (see Remarks).

**Language of species description.** English.

**Remarks.** Additional historical and morphological details of this species have been published after the original description (Bouvier 1905: 353). The holotype has not been designated explicitly in the original description. The type locality of *Eoperipatussumatranus* is surrounded by speculations. The only specimen known was found “in a bottle containing insects from East Sumatra” (Sedgwick 1888: 202). Van der Lande and Holthuis (1986) stated that the type locality of this species is unlikely to be in Sumatra, as the collector assigned to the specimen jar (W.J.E. Hekmeijer) may have never been to this island, and suggested the Mount Arjuno in East Java as a putative type locality of *Eoperipatussumatranus* (van der Lande and Holthuis 1986: 15). However, this assumption can be neither confirmed nor ruled out unambiguously, as onychophorans have never been recollected in either Java or Sumatra. Therefore, Oliveira et al. (2012a: 6) regarded *Eoperipatussumatranus* as a nomen dubium, since neither the type specimen nor the type locality is known, thus precluding an unambiguous revision of this species.

##### III. *Epiperipatus* Clark, 1913a

**Type species.***Epiperipatusedwardsii* (Blanchard, 1847), by original designation (Clark 1913a: 17).

**Remarks.**Epiperipatus has originally been introduced as a subgenus of Peripatus (see Clark 1913a) but it is commonly treated at the generic rank in the literature (e.g., Read 1988a: 189; Costa et al. 2021: 764). To prevent taxonomic instability, *Epiperipatus* is regarded herein as a valid genus. *Epiperipatus* has recently been revised by Costa et al. (2021) using morphological and molecular data and several nomenclatural changes/synonymizations have been suggested ‘to avoid paraphyly’ of this taxon (Costa et al. 2021: 789). For instance, *Principapillatus*Oliveira et al., 2012b and *Cerradopatus*Oliveira et al., 2015 have been declared as junior synonyms of *Epiperipatus*. Since the analyses of Costa et al. (2021) only included four of the 13 species that fix genus names within of Peripatidae [= type species] and, since some of the missing taxa are subject to nomenclatural priority (ICZN Art. 23.1), it may be still premature to suggest synonymizations among genera of Peripatidae. For the sake of nomenclatural stability, and following the ICZN (Art. 23.3.6), I opted for retaining *Principapillatus* and *Cerradopatus* as valid names. Also, additional species reclassified within *Epiperipatus* by Costa et al. (2021: 789) are treated herein under their previous combination. *Epiperipatus*, as defined by Costa et al. (2021), is referred to as sensu lato in the present checklist.

**6. *Epiperipatusacacioi* (du Bois-Reymond Marcus & Marcus, 1955)**

**Synonyms.***Peripatusacacioi*, as originally described (du Bois-Reymond Marcus and Marcus 1955: 189); *Peripatusouropretanus* Trindade, 1958 (junior synonym, nomen nudum; Trindade 1958: 520); Peripatus (Macroperipatus) acacioi (Froehlich 1968: 168); *Macroperipatusacacioi* (Peck 1975: 346); *Epiperipatusacacioi* (Oliveira et al. 2010: 21) (see Remarks).

**Holotype.** Not designated (see Remarks).

**Type locality.** Brazil, Minas Gerais, Ouro Preto, Estação Ecológica do Tripuí, 20°22.95'S, 43°33.05'W, ca 1,215 m ASL (see Remarks).

**Language of species description.** English.

**Remarks.** The full name of the first author [Eveline du Bois-Reymond Marcus] is used herein to disambiguate the authors’ names provided in the original species description [Eveline Marcus & Ernest Marcus]. The name *Peripatusouropretanus* Trindade, 1958 has been regarded as nomen nudum by Oliveira et al. (2012a: 7). The species name has been misspelt as *Penipatus* [sic] (*Macroperipatus*) *acacioi* in Vasconcellos et al. (2004: 140). The holotype has not been designated explicitly in the original description. Specimens deposited in the Museu de Zoologia da Universidade de São Paulo, São Paulo, Brazil and referred to as holotype and paratype by Sampaio-Costa et al. (2009: 557) and Oliveira et al. (2010: 21) should be regarded as syntypes. Geographic coordinates correspond to ‘Tripuí population site 2’ of Lacorte et al. (2011b: 2779). Species redescribed by Oliveira et al. (2010) and characterized molecularly by Lacorte et al. (2011a, b).

**7. *Epiperipatusadenocryptus* Oliveira, Lacorte, Fonseca, Wieloch & Mayer, 2011**

**Synonyms.***Epiperipatusanalogos* Lacorte, Oliveira & Fonseca, 2010 (nomen nudum; Lacorte et al. 2010: 342) (see Remarks).

**Holotype.** Deposited in the Zoology Department of the Instituto de Ciências Biológicas, Universidade Federal de Minas Gerais, Belo Horizonte, Brazil.

**Type locality.** Brazil, Minas Gerais, Santa Bárbara do Leste, Córrego dos Ferreiras, 19°58.98'S, 42°06.76'W, ca 1,050 m ASL.

**Language of species description.** English.

**Remarks.** The name *Epiperipatusanalogos* Lacorte, Oliveira & Fonseca, 2010 is a nomen nudum (see Oliveira et al. 2012a: 7), as it was suggested prematurely in an abstract by Lacorte et al. (2010: 342) without formal description or type designation. *Epiperipatusadenocryptus* is morphologically very similar to *Epiperipatuspaurognostus*. Species described based on molecular and morphological data (Oliveira et al. 2011).

**8. *Epiperipatusbarbadensis* (Froehlich, 1962)**

**Synonyms.**Peripatus (Peripatus) dominicae barbadensis, as originally described (Froehlich 1962: 325); *Peripatusdominicaebarbadensis* (Peck 1975: 348); *Epiperipatusbarbadensis* (Read 1988b: 237).

**Holotype.** Deposited in the Zoology Department of the Universidade de São Paulo, São Paulo, Brazil.

**Type locality.** Barbados Island, St. John, Codrington College.

**Language of species description.** English.

**Remarks.** Collection material presumably in suboptimal conditions for morphological and/or molecular analyses (Costa et al. 2021: 764). Requires morphological revision and molecular characterization.

**9. *Epiperipatusbarbouri* (Brues, 1911)**

**Synonyms.***Peripatusbarbouri*, as originally described (Brues 1911: 305); *Epiperipatusbarbouri* (Peck 1975: 345).

**Holotype.** Deposited in the Museum of Comparative Zoology, Harvard University, Cambridge, USA.

**Type locality.** Grenada Island, Grand Etang, ca 550 m [1,800 ft] ASL.

**Language of species description.** English.

**Remarks.** Species redescribed by Read (1988b). *Epiperipatusbarbouri* has not been included in the recent revision of *Epiperipatus* sensu lato (Costa et al. 2021). Requires morphological revision and molecular characterization.

**10. *Epiperipatusbeckeri* Costa, Chagas-Júnior & Pinto-da-Rocha, 2018**

**Synonyms.** None.

**Holotype.** Previously deposited in the Museu Nacional do Rio de Janeiro, Rio de Janeiro, Brazil (see Remarks).

**Type locality.** Brazil, Bahia, Camacan, RPPN [= Particular Reserve of Natural Patrimony] Serra Bonita, 15°23.08'S, 39°32.97'W, ca 240 m ASL (see Remarks).

**Language of species description.** English.

**Remarks.** The holotype has confirmedly been destroyed during the fire that consumed great part of collections held at the Museu Nacional do Rio de Janeiro in 2019 (A.B. Kury and C.S. Costa, pers. comm. 2023). Paratypes are deposited in the Museu de Zoologia da Universidade de São Paulo, São Paulo, Brazil (Costa et al. 2018: 8). Geographic coordinates obtained from Costa et al. (2021: 766); altitude data obtained from Google Earth based on the given coordinates. Molecular data have subsequently been provided for this species by Costa et al. (2021).

**11. *Epiperipatusbernali* Costa & Giribet, 2021**

**Synonyms.** None.

**Holotype.** Deposited in the Museu de Zoologia da Universidade de São Paulo (MZUSP), São Paulo, Brazil.

**Type locality.** Panama, Chiriquí, San José de David, Campus of the Universidad Autónoma de Chiriquí, 8°25.97'N, 82°27.12'W, ca 30 m ASL (see Remarks).

**Language of species description.** English.

**Remarks.** Altitude data obtained from Google Earth based on the given geographic coordinates. *Epiperipatusbernali* is described based on morphological and molecular data (Costa and Giribet 2021); transcriptome data have recently been provided for this species (Baker et al. 2021).

**12. *Epiperipatusbetheli* (Cockerell, 1913a)**

**Synonyms.**Peripatus (Epiperipatus) biolleyi var. betheli, as originally described (Cockerell 1913a: 87); *Epiperipatusbetheli* (Oliveira et al. 2012a: 8).

**Holotype.** Deposited in the Smithsonian National Museum of Natural History, Washington D.C., USA (see Remarks).

**Type locality.** Guatemala, Puerto Barrios.

**Language of species description.** English.

**Remarks.***Epiperipatusbetheli* has been raised to species status by Oliveira et al. (2012a: 8) based on the great distance (808 km) between its type locality and the type locality of *Epiperipatusbiolleyi* (Bouvier, 1902a). The holotype has not been designated explicitly in the original description. According to Cockerell (1913a: 87), however, *Epiperipatusbetheli* was described based on a single specimen deposited in the U.S. National Museum [currently Smithsonian National Museum of Natural History in Washington D.C., USA]. This should be regarded as the holotype fixed by monotypy, following the ICZN (Art. 73.1.2). Collection material presumably in suboptimal conditions for morphological and/or molecular analyses (Costa et al. 2021: 764). Requires morphological revision and molecular characterization.

**13. *Epiperipatusbiolleyi* (Bouvier, 1902a)**

**Synonyms.***Peripatusbiolleyi*, as originally described (Bouvier 1902a: 258); Peripatus (Epiperipatus) biolleyi (Clark 1913a: 18); *Epiperipatusbiolleyi* (Peck 1975: 345).

**Holotype.** Deposited in the Museum National d’Histoire Naturelle de Paris, France (see Remarks).

**Type locality.** Costa Rica, possibly San José, Alto de la Palma between Moravia and Vázquez de Coronado, ancient Carrillo road, Lower Montane Rain Forest, 10°02.88'N, 83°59.20'W, ca 1,530 m ASL (see Remarks). Originally described as “environs of San José, 1,161 m” (Bouvier 1902a: 259).

**Language of species description.** French.

**Remarks.** Additional historical and morphological details of this species have been published after the original description (Bouvier 1905: 321). The species name has been misspelt as *boilleyi* [sic] in Walker (1986: 209). The holotype has not been designated explicitly in the original description. Since *Epiperipatusbiolleyi* has been described based on a single specimen (Bouvier 1902a: 259), the ‘type’ deposited in the Museum National d’Histoire Naturelle de Paris, France (see Bouvier 1907a: 519; Le Bras et al. 2015) should be regarded as holotype fixed by monotypy, following the ICZN (Art. 73.1.2). According to Oliveira et al. (2012a: 8), specimens matching the original description were found in Las Nubes-Cascajal de Coronado, near San José, Costa Rica (e.g., Monge-Nájera et al. 1993; Mora et al. 1996; Mayer 2006a, b), but recent historical evidences suggest that *Epiperipatusbiolleyi* was originally collected 6 km away from Las Nubes-Cascajal de Coronado, at Alto de la Palma in the province of San José (Barquero-González et al. 2016: 1406); specimens from these two localities are likely to be conspecific. The term ‘neotype locality’ used by Barquero-González et al. (2016: 1407) is not recognized by the ICZN (Art. 76) and could be misleading, considering that a neotype cannot be designated for as long as the holotype still exists in the Museum National d’Histoire Naturelle de Paris (see ICZN Art. 75.1). Oliveira et al. (2012a) regarded *Epiperipatusbiolleyi* and *Epiperipatusbetheli* as separate species due to the great distance (808 km) between their type localities. Additional morphological data for *Epiperipatusbiolleyi* have been provided by Oliveira et al. (2012b); the complete mitochondrial genome (Podsiadlowski et al. 2008; Rota-Stabelli et al. 2010) and additional molecular data are available for this species (e.g., Oliveira et al. 2012b; Murienne et al. 2014; Giribet et al. 2018; Costa et al. 2021).

**14. *Epiperipatusbrasiliensis* (Bouvier, 1899a)**

**Synonyms.***Peripatussantarem* Sedgwick, 1888 (senior synonym, nomen oblitum; Sedgwick 1888: 484); *Peripatusbrasiliensis* Bouvier, 1899a, as originally described (junior synonym, nomen protectum; Bouvier 1899a: 1031); Peripatus (Epiperipatus) brasiliensis (Clark 1913a: 18); *Epiperipatusbrasiliensisbrasiliensis* (Peck 1975: 345); *Epiperipatusbrasiliensis* (Oliveira et al. 2012a: 9) (see Remarks).

**Holotype.** Not designated (see Remarks).

**Type locality.** Brazil, Pará, Santarém.

**Language of species description.** French.

**Remarks.** Additional historical and morphological details of this species have been published after the original description (Bouvier 1905: 269). Oliveira et al. (2012a: 9) regarded the name *Peripatusbrasiliensis* Bouvier, 1899a as nomen protectum and the senior synonym *Peripatussantarem* Sedgwick, 1888 (Sedgwick 1888: 484) as nomen oblitum, following the rules of the ICZN (Art. 23.9.1). The holotype has not been designated explicitly in the original description. According to Bouvier (1905: 270), specimens collected by W.H.J. Carter and placed in the Natural History Museum of London do not represent type specimens (see Oliveira et al. 2012a: 9). Species name is commonly misspelt as *braziliensis* (e.g., Arnett 1947: 59; Eakin and Brandenburger 1966: 507). Oliveira et al. (2012a: 9) considered the subspecies *Epiperipatusbrasiliensisvagans* (Brues, 1925) as a separate species. Molecular data recently assigned to *Epiperipatusbrasiliensis* (see Costa et al. 2021) may not strictly correspond to the original species, as the sequenced specimen was collected ~ 52 km away from the type locality.

**15. *Epiperipatusbroadwayi* (Clark, 1913b)**

**Synonyms.**Peripatus (Epiperipatus) trinidadensis var. broadwayi (Clark 1913b: 255); *Epiperipatustrinidadensisbroadwayi* (Peck 1975: 346); *Epiperipatusbroadwayi* (Read 1988b: 245).

**Holotype.** Not designated (see Remarks).

**Type locality.** Republic of Trinidad and Tobago, Tobago Island, possibly the environs of Charlotteville at the northeastern border of the Tobago Main Ridge Forest Reserve (see Remarks). Originally described as “Botanic station, Tobago” (Clark 1913b: 254).

**Language of species description.** Italian.

**Remarks.** The holotype has not been designated explicitly in the original description. The precise type locality is not provided in the original description (Clark 1913b: 255). According to Read (1988b: 245), the species occurs in a large area spanning from Scarborough to Charlotteville, while most specimens were found at ‘the forested eastern end of the island’, along the Windward Road and the Charlotteville-L’Anse Fourmi Road, an area which corresponds to the northeastern border of the Tobago Main Ridge Forest Reserve (see Read 1988b for further details). Whether or not additional species occur on the island is unclear. The annotated draft genome of *Epiperipatusbroadwayi* has recently been provided for a specimen collected at 11°17.04'N, 60°36.43'W, ca 500 m ASL (Sato et al. 2023). Additional molecular data are also available for this species (Giribet et al. 2018; Baker et al. 2021).

**16. *Epiperipatuscratensis* Brito, Pereira, Ferreira, Vasconscellos & Almeida, 2010**

**Synonyms.** None.

**Holotype.** Deposited in the Coleção de Invertebrados da Universidade Regional do Cariri, Crato, Brazil.

**Type locality.** Brazil, Ceará, Crato, Rio Batateiras, 07°16'S, 39°26'W, ca 700 m ASL.

**Language of species description.** English.

**Remarks.** Molecular data recently available for this species (e.g., Giribet et al. 2018; Costa et al. 2021).

**17. *Epiperipatusdiadenoproctus* Oliveira, Lacorte, Fonseca, Wieloch & Mayer, 2011**

**Synonyms.***Epiperipatusdiadenoproctus* Lacorte, Oliveira & Fonseca, 2010 (nomen nudum; Lacorte et al. 2010: 342) (see Remarks).

**Holotype.** Deposited in the Department of Zoology of the Instituto de Ciências Biológicas da Universidade Federal de Minas Gerais, Belo Horizonte, Brazil.

**Type locality.** Brazil, Minas Gerais, Simonésia, RPPN [= Particular Reserve of Natural Patrimony] Mata do Sossego, 20°04.35'S, 42°04.20'W, ca 1,150 m ASL.

**Language of species description.** English.

**Remarks.** The name *Epiperipatusdiadenoproctus* has been suggested prematurely in an abstract by Lacorte et al. (2010: 342) without formal description or type designation, thus not fulfilling the criteria of publication and availability established by the ICZN (Art. 9.10, 13.1, and 16.4). Thus, *Epiperipatusdiadenoproctus* Lacorte, Oliveira & Fonseca, 2010 is a nomen nudum. The name has become available one year later (Oliveira et al. 2011) based on the same material studied by Lacorte et al. (2010). *Epiperipatusdiadenoproctus* is described based on molecular and morphological data (Oliveira et al. 2011).

**18. *Epiperipatusedwardsii* (Blanchard, 1847)**

**Synonyms.***Peripatusedwardsii*, as originally described (Blanchard 1847: 140); Peripatus (Epiperipatus) edwardsii (Clark 1913a: 18); *Epiperipatusedwardsii* (Peck 1975: 345).

**Holotype.** Deposited in the Museum National d’Histoire Naturelle de Paris, France (see Remarks).

**Type locality.** French Guiana, Cayenne (arrondissement), Cayenne, on the banks of the Approuague River, 12 km [3 leagues] from its mouth (see Remarks).

**Language of species description.** French.

**Remarks.** The species name is commonly misspelt as *edwardsi* (e.g., Bouvier 1905: 162; Peck 1975: 345). Additional historical and morphological details of this species have been published after the original description (Bouvier 1905: 301). The holotype has not been designated explicitly in the original description. Since *Epiperipatusedwardsii* has been described based on a single specimen (Blanchard 1847: 139, 140; Audouin and Milne-Edwards 1833: 414), the ‘type’ deposited in the Museum National d’Histoire Naturelle de Paris, France (Bouvier 1907a: 519; Le Bras et al. 2015) should be regarded as holotype fixed by monotypy, following the ICZN (Art. 73.1.2). The original species description does not include precise locality data: ‘Cayenne’ (Blanchard 1847: 140) may refer to either the city or to the entire administrative division (arrondissement), with the latter occupying an area of 42,589 km^2^. However, Blanchard (1847: 140) raised the species name based on the same specimen previously studied by Audouin and Milne-Edwards, which has been collected “on the banks of the Approuague River, 12 km [3 leagues] from its mouth” (Audouin and Milne-Edwards 1833: 414); this should be regarded as the species type locality. Also, the authors most likely refer to the French metric league (1 league = 4 km) used in France between 1812–1840. *Epiperipatusedwardsii* has previously been recorded from an extensive area spanning from Brazil to Panama (Peck 1975; Read 1988b: 251, 253). A recent redescription work suggested that this species is restricted to the Nouragues Nature Reserve (Nouragues Field Station in Costa et al. 2018), between the cities of Régina and Roura in the arrondissement of Cayenne (Costa et al. 2018: 4), but since this locality lies more than 80 km from the point described by Audouin and Milne-Edwards (1833: 414), the existence of a species complex within *Epiperipatusedwardsii* cannot be excluded. For the sake of caution, the name *Epiperipatusedwardsii* should only be applied to specimens from the type locality. Molecular data recently assigned to *Epiperipatusedwardsii* (e.g., Murienne et al. 2014; Giribet et al. 2018; Costa et al. 2021) may not strictly correspond to the original species, as the sequenced specimen was collected ~ 82 km away from the type locality. Requires revision.

**19. *Epiperipatushilkae* Morera-Brenes & Monge-Nájera, 1990**

**Synonyms.** None.

**Holotype.** Deposited in the Museo de Zoologia de la Universidad de Costa Rica, San José, Costa Rica.

**Type locality.** Costa Rica, Guanacaste, Nicoya, Parque Nacional Barra Honda, Bosque de las Cascadas, 10°11'N, 85°20'W, ca 200 m ASL (see Remarks).

**Language of species description.** English.

**Remarks.** The species was described based on specimens from different localities lying 62 km apart from each other. According to Oliveira et al. (2012a: 10) and Barquero-González et al. (2016: 1409), the existence of a species complex within *Epiperipatushilkae* cannot be excluded. For the sake of caution, the name *Epiperipatushilkae* should only be applied to specimens from the type locality. Species not included in the recent revision of *Epiperipatus* sensu lato (Costa et al. 2021). Requires morphological revision and molecular characterization.

**20. *Epiperipatushyperbolicus* Costa, Chagas-Júnior & Pinto-da-Rocha, 2018**

**Synonyms.** None.

**Holotype.** Previously deposited in the Museu Nacional do Rio de Janeiro, Rio de Janeiro, Brazil (see Remarks).

**Type locality.** Brazil, Alagoas, Murici, Ecological Station of Murici, Mata da Bananeira, 9°15.13'S, 35°47.89'W, ca 335 m ASL (see Remarks).

**Language of species description.** English.

**Remarks.** The holotype and paratypes have confirmedly been destroyed during the fire that consumed great part of the collections held at the Museu Nacional do Rio de Janeiro in 2019 (A.B. Kury and C.S. Costa, pers. comm. 2023). Geographic coordinates obtained from Costa et al. (2021: 766); altitude data obtained from Google Earth based on the given coordinates. Molecular data have subsequently been provided for this species by Costa et al. (2021).

**21. *Epiperipatusimthurni* (Sclater, 1888)**

**Synonyms.***Peripatusdemeraranus* Sedgwick, 1888 (junior synonym, nomen nudum; Sedgwick 1888: 476); *Peripatusimthurni* Sclater, 1888, as originally described (Sclater 1888: 344); Peripatus (Epiperipatus) imthurmi (Clark 1913a: 18); *Epiperipatusimthurmi* (Peck 1975: 345) (see Remarks).

**Holotype.** Not designated (see Remarks).

**Type locality.** Co-operative Republic of Guyana, possibly Maccaseema on Pomeroon River (see Remarks). Originally described as “from Demerara” (Sclater 1888: 343).

**Language of species description.** English.

**Remarks.** Additional historical and morphological details of this species have been published after the original description (Bouvier 1905: 275). Species name commonly misspelt as *imthurmi* (e.g., Clark 1913a: 18; Peck 1975: 345; Sampaio-Costa et al. 2009: 559). The name *Peripatusdemeraranus* has been suggested one month after the description of *Peripatusimthurni*, thus constituting a junior synonym of this species. To avoid confusion, *Peripatusdemeraranus* Sedgwick, 1888 has been regarded as nomen nudum by Oliveira et al. (2012a: 11). The holotype has not been designated explicitly in the original description. Bouvier (1907a: 519) refers to a cotype [obsolete for syntype] deposited in the Museum National d’Histoire Naturelle de Paris, France (see also Le Bras et al. 2015). The precise type locality is not provided in the original description: according to Oliveira et al. (2012a), the name Demerara could mean the river Demerara or the Demerara region (previous name of the Co-operative Republic of Guyana), but since specimens studied by Sedgwick (1888: 474) were the same ones analyzed by Sclater (1888) (see Evans 1903: 156), it is likely that Maccaseema on Pomeroon River is the correct locality for this species. This species name has also been assigned to specimens from other localities, including the Trinidad Island (Read 1988b: 241). Hence, the existence of a species complex within *Epiperipatusimthurni* cannot be excluded. For the sake of caution, the name *Epiperipatusimthurni* should only be applied to specimens from the type locality. Collection material is presumably in suboptimal conditions for morphological and/or molecular analyses (Costa et al. 2021: 764). Requires morphological revision and molecular characterization.

**22. *Epiperipatusisthmicola* (Bouvier, 1902b)**

**Synonyms.**Peripatusnicaraguensisvar.isthmicola, as originally described (Bouvier 1902b: 240); Peripatus (Epiperipatus) isthmicola (Clark 1913a: 18); *Epiperipatusisthmicola* (Peck 1975: 345).

**Holotype.** Deposited in the Museum National d’Histoire Naturelle de Paris, France (see Remarks).

**Type locality.** Costa Rica, possibly Barrio Cristo Rey, Hospital district, San José, 9°55.50'N, 84°05.08'W, ca 1,140 m ASL (see Remarks). Originally described as “Costa Rica, San José, 1100 m” (Bouvier 1902b: 240).

**Language of species description.** French.

**Remarks.** Additional historical and morphological details of this species have been published after the original description (Bouvier 1905: 329). The holotype has not been designated explicitly in the original description. Since *Epiperipatusisthmicola* has been described based on a single specimen (Bouvier 1902b: 239), the ‘type’ deposited in the Museum National d’Histoire Naturelle de Paris, France (see Bouvier 1907a: 519; Le Bras et al. 2015) should be regarded as holotype fixed by monotypy, following the ICZN (Art. 73.1.2). The original description contains imprecise type locality data (Bouvier 1902b) and the subsequent redescription of this species (Bouvier 1905) was based on specimens from different localities. According to Barquero-González et al. (2016: 1406), the type locality of *Epiperipatusisthmicola* is likely to be the Barrio Cristo Rey in San José, although the area is to date heavily urbanized. The term ‘neotype locality’ used by Barquero-González et al. (2016: 1403) is not recognized by the ICZN (Art. 76) and could be misleading, considering that a neotype cannot be designated for as long as the holotype still exists in the Museum National d’Histoire Naturelle de Paris (see ICZN Art. 75.1). Also, the term ‘neotype’ may have mistakenly been used by Barquero-González et al. (2016: 1406) while meaning topotype. Molecular data recently assigned to *Epiperipatusisthmicola* (see Giribet et al. 2018; Costa et al. 2021) may not strictly correspond to the original species, as the sequenced specimen was collected ~ 30 km away from the type locality. Since the existence of a species complex within *Epiperipatusisthmicola* cannot be excluded, closer revision is required. For the sake of caution, the name *Epiperipatusisthmicola* should only be applied to specimens from the type locality.

**23. *Epiperipatuslewisi* Arnett, 1961**

**Synonyms.** None.

**Holotype.** Deposited in the Smithsonian National Museum of Natural History, Washington D.C., USA.

**Type locality.** Jamaica, Portland, John Crow Mountains, ca 16 km [10 miles] southwest of Priestman’s River, ca 455 m [1,500 ft] ASL (see Remarks).

**Language of species description.** English.

**Remarks.** Altitude data provided by Peck (1975: 346). Species not included in the recent revision of *Epiperipatus* sensu lato (Costa et al. 2021). Requires morphological revision and molecular characterization.

**24. *Epiperipatuslucerna* Costa, Chagas-Júnior & Pinto-da-Rocha, 2018**

**Synonyms.** None.

**Holotype.** Previously deposited in the Museu Nacional do Rio de Janeiro, Rio de Janeiro, Brazil (see Remarks).

**Type locality.** Brazil, Alagoas, Murici, Ecological Station of Murici, Mata da Bananeira, 9°15.13'S, 35°47.89'W, ca 335 m ASL (see Remarks).

**Language of species description.** English.

**Remarks.** The holotype has confirmedly been destroyed during the fire that consumed great part of the collections held at the Museu Nacional do Rio de Janeiro in 2019 (A.B. Kury and C.S. Costa, pers. comm. 2023). A paratype is deposited in the Museu de Zoologia da Universidade de São Paulo (MZUSP), São Paulo, Brazil (Costa et al. 2018: 6). Geographic coordinates obtained from Costa et al. (2021: 766); altitude data obtained from Google Earth based on the given coordinates. Molecular data have subsequently been provided for this species by Costa et al. (2021).

**25. *Epiperipatusmachadoi* (Oliveira & Wieloch, 2005)**

**Synonyms.***Macroperipatusmachadoi*, as originally described (Oliveira and Wieloch 2005: 61); *Epiperipatusmachadoi* (Oliveira et al. 2010: 25).

**Holotype.** Deposited in the Department of Zoology of the Instituto de Ciências Biológicas, Universidade Federal de Minas Gerais, Belo Horizonte, Brazil.

**Type locality.** Brazil, Minas Gerais, Caratinga, RPPN Feliciano Miguel Abdala, 19°43.87'S, 41°49.03'W, ca 410 m ASL.

**Language of species description.** Portuguese.

**Remarks.** Species redescribed by Oliveira et al. (2010). Molecular data available for this species (e.g., Lacorte et al. 2011a; Costa et al. 2021).

**26. *Epiperipatusmarajoara* Costa, Chagas-Júnior & Pinto-da-Rocha, 2018**

**Synonyms.** None.

**Holotype.** Deposited in the Museu de Zoologia da Universidade de São Paulo (MZUSP), São Paulo, Brazil.

**Type locality.** Brazil, Pará, Marajó Island, Breves, Extractive Reserve Mapuá, 01°02.90'S, 50°28.78'W, ca 15 m ASL (see Remarks).

**Language of species description.** English.

**Remarks.** Geographic coordinates obtained from Costa et al. (2021: 766); altitude data obtained from Google Earth based on the given coordinates. Molecular data have subsequently been provided for this species by Costa et al. (2021).

**27. *Epiperipatusohausi* (Bouvier, 1900a)**

**Synonyms.***Peripatusohausi*, as originally described (Bouvier 1900a: 67); Peripatus (Macroperipatus) ohausi (Clark 1913a: 17); *Macroperipatusohausi* (Peck 1975: 347); *Epiperipatusohausi* (Chagas-Júnior and Costa 2014: 979).

**Holotype.** Not designated (see Remarks).

**Type locality.** Brazil, Rio de Janeiro, Petrópolis (see Remarks).

**Language of species description.** French.

**Remarks.** Additional historical and morphological details of this species have been published after the original description (Bouvier 1905: 204). The holotype has not been designated explicitly in the original description. Bouvier (1907a: 518) refers to a cotype [obsolete for syntype] deposited in the Museum National d’Histoire Naturelle de Paris, France (see also Le Bras et al. 2015). The holotype mentioned by Weidner (1959: 93) in the Zoologisches Staatsinstitut und Zoologisches Museum Hamburg, Hamburg, Germany should be regarded as syntype. The description contains imprecise type locality data (Bouvier 1900a: 66): the city of Petrópolis covers an area of 796 km^2^. Species has been re-described based on specimens collected in the RPPN [= Particular Reserve of Natural Patrimony] dos Petroleiros, Rio de Janeiro, Nova Iguaçu (22°35.75'S, 43°26.13'W, 89 m), approximately 28 km away from Petrópolis (Chagas-Júnior and Costa 2014). Molecular data have subsequently been provided for specimens from Nova Iguaçu (Costa et al. 2021). Whether or not specimens from Petrópolis and Nova Iguaçu are conspecific remains unclear due to the lack of data from either the syntype or topotypes.

**28. *Epiperipatuspaurognostus* Oliveira, Lacorte, Fonseca, Wieloch & Mayer, 2011**

**Synonyms.***Epiperipatusschedocrypticus* Lacorte, Oliveira & Fonseca, 2010 (nomen nudum; Lacorte et al. 2010: 342) (see Remarks).

**Holotype.** Deposited in the Department of Zoology of the Instituto de Ciências Biológicas, Universidade Federal de Minas Gerais, Belo Horizonte, Brazil.

**Type locality.** Brazil, Minas Gerais, Piedade de Caratinga, Mata do Eremitério, 19°45.55'S, 42°05.37'W, ca 895 m ASL.

**Language of species description.** English.

**Remarks.** The name *Epiperipatusschedocrypticus* Lacorte, Oliveira & Fonseca, 2010 is a nomen nudum (see Oliveira et al. 2012a: 12), as it has been suggested prematurely in an abstract by Lacorte et al. (2010: 342) without formal description or type designation. *Epiperipatuspaurognostus* is morphologically very similar to *Epiperipatusadenocryptus*. Species described based on molecular and morphological data (Oliveira et al. 2011).

**29. *Epiperipatussimoni* (Bouvier, 1899b)**

**Synonyms.***Peripatussimoni*, as originally described (Bouvier 1899b: 271); Peripatus (Epiperipatus) simoni (Clark 1913a: 18); *Epiperipatussimoni* (Peck 1975: 346).

**Holotype.** Deposited in the Museum National d’Histoire Naturelle de Paris, France (see Remarks).

**Type locality.** Venezuela, possibly the San Esteban National Park, near Caracas (see Remarks). Originally described as “Venezuela” (Bouvier 1899b: 270).

**Language of species description.** French.

**Remarks.** Additional historical and morphological details of this species have been published after the original description (Bouvier 1905: 315). The holotype has not been designated explicitly in the original description. Since *Epiperipatussimoni* has been described based on a single specimen (Bouvier 1899c: 408), the ‘type’ deposited in the Museum National d’Histoire Naturelle de Paris, France (see Bouvier 1907a: 519; Le Bras et al. 2015) should be regarded as holotype fixed by monotypy, following the ICZN (Art. 73.1.2). According to Oliveira et al. (2012a: 12), the type locality for the species might be the San Esteban National Park, near Caracas (see Bouvier 1905: 221). The species name has been misspelt as *F.semoni* [sic] by Vasconcellos et al. (2004: 140). Species not included in the recent revision of *Epiperipatus* sensu lato (Costa et al. 2021). Requires morphological revision and molecular characterization.

**30. *Epiperipatustitanicus* Costa, Chagas-Júnior & Pinto-da-Rocha, 2018**

**Synonyms.** None.

**Holotype.** Previously deposited in the Museu Nacional do Rio de Janeiro, Rio de Janeiro, Brazil (see Remarks).

**Type locality.** Brazil, Alagoas, Murici, Ecological Station of Murici, Mata da Bananeira, 9°15.13'S, 35°47.89'W, ca 335 m ASL (see Remarks).

**Language of species description.** English.

**Remarks.** The holotype and 15 paratypes have confirmedly been destroyed during the fire that consumed great part of the collections held at the Museu Nacional do Rio de Janeiro in 2019 (A.B. Kury and C.S. Costa, pers. comm. 2023). Additional paratypes are deposited in the Department of Zoology of the Instituto de Ciências Biológicas, Universidade Federal de Minas Gerais, Belo Horizonte, Brazil and Museu de Zoologia da Universidade de São Paulo (MZUSP), São Paulo, Brazil (Costa et al. 2018: 5). Geographic coordinates obtained from Costa et al. (2021: 766); altitude data obtained from Google Earth based on the given coordinates. Molecular data have subsequently been provided for this species by Costa et al. (2021).

**31. *Epiperipatustorrealbai* (Scorza, 1953)**

**Synonyms.**Peripatus (Epiperipatus) torrealbai as originally described (Scorza 1953: 785); *Epiperipatustorrealbai* (Oliveira et al. 2012a: 12) (see Remarks).

**Holotype.** Deposited in the Museo de Zoologia de la Universidad Central de Venezuela, Caracas, Venezuela.

**Type locality.** Venezuela, Los Chorros, near Caracas.

**Language of species description.** Spanish.

**Remarks.**Scorza (1953) uses different spellings for the species, including Peripatus (Epiperipatus) torrealbai (p. 785), *Peripatustorrealbai* (p. 787), and *Epiperipatustorrealbai* (p. 787). Species not included in the recent revision of *Epiperipatus* sensu lato (Costa et al. 2021). Requires morphological revision and molecular characterization.

**32. *Epiperipatustrinidadensis* (Sedgwick, 1888)**

**Synonyms.***Peripatustrinidadensis*, as originally described (Sedgwick 1888: 477); *Peripatustrinitatis* (Bouvier 1905: 289); Peripatus (Epiperipatus) trinidadensis (Clark 1913a: 18); *Epiperipatustrinidadensis* (Peck 1975: 346).

**Holotype.** Not designated (see Remarks).

**Type locality.** Republic of Trinidad and Tobago, Trinidad Island, possibly Northern Range of Trinidad, Simla Research Station, 6.4 km [4 miles] north of Arima (see Remarks). Originally described as “Trinidad” (Sedgwick 1888: 487).

**Language of species description.** English.

**Remarks.** An incorrect author (Stuhlmann) is commonly attributed to this species (e.g., Bouvier 1905: 290; Peck 1975: 346), although the referred publication does not include the species name (Stuhlmann 1886). Additional historical and morphological details of this species have been published after the original description (Bouvier 1905: 289). The holotype has not been designated explicitly in the original description. Bouvier (1907a: 519) refers to a cotype [obsolete for syntype] deposited in the Museum National d’Histoire Naturelle de Paris, France (see also Le Bras et al. 2015). The precise type locality has not been provided in the original description. A subsequent redescription of this species recorded it from different localities in the Northern Range of Trinidad (Read 1988b: 247), with most specimens being collected in the Simla Research Station, 6.4 km [4 miles] north of Arima (Read 1988b: 248). Since the existence of a species complex within *Epiperipatustrinidadensis* cannot be excluded, revision is required. Collection material is presumably in suboptimal conditions for morphological and/or molecular analyses (Costa et al. 2021: 764), although transcriptome data have recently been provided for this species (Baker et al. 2021).

**33. *Epiperipatusvagans* (Brues, 1925)**

**Synonyms.**Peripatus (Epiperipatus) brasiliensis var. vagans, as originally described (Brues 1925: 162); *Epiperipatusbrasiliensisvagans* (Peck 1975: 345); *Epiperipatusvagans* (Oliveira et al. 2012a: 13).

**Holotype.** Deposited in the Museum of Comparative Zoology at Harvard University, Cambridge, USA (see Remarks).

**Type locality.** Panama, former Canal Zone, Barro Colorado Island.

**Language of species description.** English.

**Remarks.**Brues (1925: 159, 163) indicates that the holotype was deposited in the U.S. National Museum [currently Smithsonian National Museum of Natural History in Washington D.C]. However, Costa and Giribet (2021: 5) stated that the holotype is now deposited in the Museum of Comparative Zoology of the Harvard University. Oliveira et al. (2012a: 13) raised *Epiperipatusvagans* to species level separated from *Epiperipatusbrasiliensis* based on the great distance between their type localities (3,066 km). The putative designation of this specie as nomen dubium (see Costa and Giribet 2021: 9, 10) could not be verified in the referred literature. Also, the use of parentheses enclosing the author and the date becomes mandatory according to the ICZN (Art. 51.3) since the species-group name is now combined with a generic name (i.e., *Epiperipatus*) other than the original one (i.e., *Peripatus*) (see Costa and Giribet 2021: 3 for alternative interpretation of the ICZN). This species has recently been re-described based on specimens from different localities (Costa and Giribet 2021), thus the existence of a species complex within *Epiperipatusvagans* cannot be excluded and further revision is still required. For the sake of caution, the name *Epiperipatusvagans* should only be applied to specimens from the type locality. Molecular data including transcriptome recently assigned to *Epiperipatusvagans* (see Giribet et al. 2018; Costa et al. 2021; Costa and Giribet 2021; Baker et al. 2021) may not strictly correspond to the original species, as the sequenced specimens were collected in the mainland Panama, 20 km away from the Barro Colorado Island.

**34. *Epiperipatusvespuccii* Brues, 1914**

**Synonyms.**Peripatus (Epiperipatus) vespuccii, as originally described (Brues 1914: 375); *Epiperipatusvespuccii* (Peck 1975: 346).

**Holotype.** Deposited in the Museum of Comparative Zoology at Harvard University, Cambridge, USA.

**Type locality.** Colombia, Magdalena, Sierra Nevada de Santa Marta, Cincinnati Coffee Plantation, ca 700 m [2,300 ft] ASL.

**Language of species description.** English.

**Remarks.** Collection material is presumably in suboptimal conditions for morphological and/or molecular analyses (Costa et al. 2021: 764). Requires morphological revision and molecular characterization.

**Nomina dubia**

**nd2. *Epiperipatusevansi* (Bouvier, 1904a)**

**Synonyms.***Peripatusevansi*, as originally described (Bouvier 1904a: 52); Peripatus (Epiperipatus) evansi (Clark 1913a: 18); *Epiperipatusevansi* (Peck 1975: 345).

**Holotype.** Not designated (see Remarks).

**Type locality.** Co-operative Republic of Guyana, east bank of the Demerara River (see Remarks).

**Language of species description.** French.

**Remarks.** Additional historical and morphological details of this species have been published after the original description (Bouvier 1905: 285). The holotype has not been designated explicitly in the original description. A syntype is deposited in the Natural History Museum of London, UK. The type locality of *Epiperipatusevansi* has mistakenly been described as Maccasseema on Pomeroon River by Oliveira et al. (2012a: 10). The collecting data provided by Evans (1903: 148) and Bouvier (1904a: 52, 1905: 288), as well as in the label accompanying the syntype, are imprecise: the Demerara River extends for more than 345 km. Collection material presumably in suboptimal conditions for morphological and/or molecular analyses (Costa et al. 2021: 764). *Epiperipatusevansi* is regarded herein as a nomen dubium since precise locality data are missing in the literature, thus precluding an unambiguous revision of this species based on topotypes.

**nd3. *Epiperipatusnicaraguensis* (Bouvier, 1900b)**

**Synonyms.***Peripatusnicaraguensis*, as originally described (Bouvier 1900b: 395); Peripatus (Epiperipatus) nicaraguensis (Clark 1913a: 18); *Epiperipatusnicaraguensis* (Peck 1975: 346).

**Holotype.** Deposited in the Museum für Naturkunde Berlin, Germany (see Remarks).

**Type locality.** Nicaragua, Matagalpa (see Remarks).

**Language of species description.** French.

**Remarks.** Additional historical and morphological details of this species have been published after the original description (Bouvier 1905: 326). The holotype has not been designated explicitly in the original description. Since *Epiperipatusnicaraguensis* has been described based on a single specimen (Bouvier 1900b: 395), the specimen deposited in the Museum für Naturkunde Berlin, Germany, should be regarded as holotype fixed by monotypy, following the ICZN (Art. 73.1.2). Note that Oliveira et al. (2012a: 14) mistakenly regarded this specimen as syntype, while Röhlig et al. (2010: 227) correctly assign it with holotype status. The description and the collection material contain imprecise type locality data: in Nicaragua, Matagalpa may refer to either a city or an entire department, with the latter occupying an area of 8,523 km^2^. Bouvier (1905: 327) stated that a specimen collected by Belt (1874: 140) could also possibly belong to this species. One the one hand, Bouvier (1905: 328) clearly misinterpreted the locality information provided by Belt, referring to San Benito – an area situated in the San Antonio Valley – instead of to San Benito Mine, which is located in the municipality of Santo Domingo (Belt 1874: 140). On the other hand, both Belt’s and Bouvier’s localities fall outside the limits of Matagalpa, suggesting that Belt’s specimen and the holotype placed in the Museum für Naturkunde Berlin are unlikely to be conspecific. The holotype has not been analyzed in the recent revision of *Epiperipatus* sensu lato (Costa et al. 2021). Oliveira et al. (2012a: 14) regarded *Epiperipatusnicaraguensis* as a nomen dubium since precise locality data are missing in the literature, thus precluding an unambiguous revision of this species based on topotypes.

**nd4. *Epiperipatustucupi* (Froehlich, 1968)**

**Synonyms.**Peripatus (Epiperipatus) tucupi, as originally described (Froehlich 1968: 168); *Epiperipatustucupi* (Peck 1975: 346).

**Holotype.** Deposited in the Museu de Zoologia da Universidade de São Paulo, São Paulo, Brazil.

**Type locality.** Brazil, Pará (see Remarks).

**Language of species description.** English.

**Remarks.** The description contains imprecise type locality data: the Pará State in Brazil occupies an area of 1,247,689.515 km^2^ within Amazonia. The holotype is presumably in suboptimal conditions for morphological and/or molecular analyses (Costa et al. 2021: 764). Oliveira et al. (2012a: 14) regarded *Epiperipatustucupi* as a nomen dubium since precise locality data are missing in the literature, thus precluding an unambiguous revision of this species based on topotypes.

##### IV. *Heteroperipatus* Zilch, 1954a

**Type species.***Heteroperipatusengelhardi* Zilch, 1954a, by original designation (Zilch 1954a: 148).

**Remarks.** Although an emended diagnosis has been provided for this genus based on characters of its type species (Oliveira et al. 2012b: 18), *Heteroperipatus* has never been re-investigated after its description, thus requiring proper morphological and molecular characterization.

**35. *Heteroperipatusclarki* (Dunn, 1943)**

**Synonyms.***Peripatusclarki*, as originally described (Dunn 1943: 2); *Heteroperipatusclarki* (Zilch 1954a: 148, as footnote).

**Holotype.** Deposited in the collection of the Academy of Natural Sciences of Philadelphia, Philadelphia, USA.

**Type locality.** Panama, Azuero, Veragua, north base of the ridge supporting Piedra del Tigre, near western border of Veragua, two days south of Las Minas, ca 790 m [2,600 ft] ASL.

**Language of species description.** English.

**Remarks.** Note that the specific epithet *clarki* has also been used by Arnett (1961) for *Macroperipatusinsularisclarki* (see *Macroperipatusclarki* below). Species likely to have been assigned to *Heteroperipatus* based on ambiguous character (Oliveira et al. 2012a: 15). Requires morphological revision and molecular characterization.

**36. *Heteroperipatusengelhardi* Zilch, 1954a**

**Synonyms.** None.

**Holotype.** probably deposited in the Senckenberg Research Institute and Natural History Museum, Frankfurt, Germany (see Remarks).

**Type locality.** El Salvador, San Vicente, Finca El Carmen, San Vicente Volcano (Las Chiches), ca 1,300 m ASL.

**Language of species description.** German.

**Remarks.** The location of the holotype is assumed based on the acronym SMF used by the author, possibly referring to his work institution [Senckenberg Museum Frankfurt] at that time (Zilch 1954a: 150). Requires morphological revision and molecular characterization.

##### V. *Macroperipatus* Clark, 1913a

**Type species.***Macroperipatustorquatus* (von Kennel, 1883), by original designation (Clark 1913a: 17).

**Remarks.**Macroperipatus has originally been introduced as a subgenus of Peripatus (see Clark 1913a) but it is commonly treated at the generic rank in the literature (e.g., Read 1988a: 189; Oliveira et al. 2010:16). To prevent taxonomic instability, *Macroperipatus* is regarded herein as a valid genus. The entire genus requires revision at morphological and molecular levels, as several species are likely to have been assigned to it due to fixation artefacts (Oliveira et al. 2010: 31). An emended diagnosis has been provided for *Macroperipatus* based on characters of its type species (Oliveira et al. 2012a: 18).

**37. *Macroperipatusclarki* Arnett, 1961**

**Synonyms.***Macroperipatusinsularisclarki*, as originally described (Arnett 1961: 215); *Macroperipatusclarki* (Oliveira et al. 2012a: 15).

**Holotype.** Deposited in the Science Museum of the Institute of Jamaica, Kingston, Jamaica.

**Type locality.** Jamaica, Portland, John Crow Mountains, ca 8 km [5 miles] southwest of the Priestman’s River, ca 455 m [1,500 ft] ASL.

**Language of species description.** English.

**Remarks.** Note that the specific epithet *clarki* has also been used by Dunn (1943) for *Peripatusclarki* (see *Heteroperipatusclarki* above). Oliveira et al. (2012a) regarded *Macroperipatusclarki* as a separated species from *Macroperipatusinsularis* given the great distance between their type localities (~ 430 km and on different islands). Requires morphological revision and molecular characterization.

**38. *Macroperipatusinsularis* Clark, 1937**

**Synonyms.***Macroperipatusinsularisinsularis* (Peck 1975: 347).

**Holotype.** Deposited in the Smithsonian National Museum of Natural Science, Washington D.C., USA.

**Type locality.** Haiti, between Jacmel and Trouin (see Remarks).

**Language of species description.** English.

**Remarks.**Peck (1975: 347) introduced the name *Macroperipatusinsularisinsularis* for distinguishing the original species from the subspecies *Macroperipatusinsularisclarki* [currently *Macroperipatusclarki*]. The locality described by Clark (1937: 3) as ‘Tronin’ may correspond to Trouin, in the arrondissement Léogâne, Ouest, Haiti. The precise type locality has not been provided in the original description: the two towns provided lie 27 km apart from each other. Oliveira et al. (2012a: 16) regarded *Macroperipatusinsularis* and *Macroperipatusclarki* as separate species based on the great distance between their type localities (~ 430 km and on different islands). Requires morphological revision and molecular characterization.

**39. *Macroperipatustorquatus* (von Kennel, 1883)**

**Synonyms.***Peripatustorquatus*, as originally described (von Kennel 1883: 532); Peripatus (Macroperipatus) torquatus (Clark 1913a: 17); *Macroperipatustorquatus* (Peck 1975: 347).

**Holotype.** Not designated (see Remarks).

**Type locality.** Republic of Trinidad and Tobago, Trinidad Island, possibly Mount Aripo, environs of Arima, ca 120 m [400 ft] ASL (see Remarks). Originally described as “Trinidad” (von Kennel 1883: 532).

**Language of species description.** German.

**Remarks.** Additional historical and morphological details of this species have been published after the original description (Bouvier 1905: 186). The holotype has not been designated explicitly in the original description. Bouvier (1907a: 518) refers to a cotype [obsolete for syntype] deposited in the Museum National d’Histoire Naturelle de Paris, France (see also Le Bras et al. 2015). The precise type locality is not provided in the original description (von Kennel 1883: 532). Read (1988b: 254), however, describes several localities for this species, all of which are located in the Northern Range of Trinidad. Specimens of *Macroperipatustorquatus* collected by I.T. Sanderson in 1937 and deposited in the Natural History Museum of London, UK, were found near Mount Aripo, east of Arima, 122 m [400 ft] (see Read 1988b: 253). The latter has been regarded as a more precise locality for this species by Oliveira et al. (2012a: 17). Molecular data including transcriptome have recently been provided for this species (Murienne et al. 2014; Giribet et al. 2018; Baker et al. 2021).

**40. *Macroperipatusvalerioi* Morera-Brenes & León, 1986**

**Synonyms.** None.

**Holotype.** Deposited in the Museo de Insectos de la Universidad de Costa Rica, Costa Rica.

**Type locality.** Costa Rica, Puntarenas, Parrita, San Antonio, southern side of Fila Chonta, 9°33.35'N, 84°11.51'W, ca 300 m ASL (see Remarks). Originally described as Rio Damitas, 16 km north of Puerto Quepos, 9°34'N, 84°10'W, 600 m (Morera-Brenes and León 1986: 277).

**Language of species description.** English.

**Remarks.** More precise locality for this species is provided in Barquero-González et al. (2016: 1408). Molecular data including transcriptome recently assigned to *Macroperipatusvalerioi* (see Giribet et al. 2018; Baker et al. 2021) were obtained from specimens collected ca 26 km away from the type locality. Requires revision.

**Nomina dubia**

**nd5. *Macroperipatusgeayi* (Bouvier, 1899d)**

**Synonyms.***Peripatusgeayi*, as originally described (Bouvier 1899d: 1345); Peripatus (Macroperipatus) geayi (Clark 1913a: 17); *Macroperipatusgeayi* (Peck 1975: 246).

**Holotype.** Deposited in the Museum National d’Histoire Naturelle de Paris, France (see Remarks).

**Type locality.** Brazil, Amapá, Carsevenne [= Calçoene], possibly high Carsevenne River (see Remarks). Originally described as “from Carsevenne” (Bouvier 1899d: 1345).

**Language of species description.** French.

**Remarks.** Additional historical and morphological details of this species have been published after the original description (Bouvier 1905: 200). The holotype has not been designated explicitly in the original description. Since *Macroperipatusgeayi* has been described based on a single specimen (Bouvier 1899c: 404), the ‘type’ deposited in the Museum National d’Histoire Naturelle de Paris, France (see Bouvier 1905: 201, 1907a: 519; Le Bras et al. 2015) should be regarded as holotype fixed by monotypy, following the ICZN (Art. 73.1.2). The description contains imprecise type locality data: the Calçoene region occupies 14,269 km^2^ of the Amapá State, Brazil. The syntype label says ‘high Carsevenne’, possibly referring to the river with the same name, which crosses the region (Oliveira et al. 2012a: 17). The species name was misspelt as *geagy* [sic] by Jerez-Jaimes and Bernal-Pérez (2009: 567) and as *geagi* [sic] by Morera-Brenes and León (1986: 278). Oliveira et al. (2012a: 17) regarded *Macroperipatusgeayi* as a nomen dubium since precise locality data are missing in the literature, thus precluding an unambiguous revision of this species based on topotypes.

**nd6. *Macroperipatusguianensis* (Evans, 1903)**

**Synonyms.***Peripatusguianensis*, as originally described (Evans 1903: 145); Peripatusohausivar.guianensis (Bouvier 1904a: 53); Peripatus (Macroperipatus) guianensis (Clark 1913a: 17); *Macroperipatusguianensis* (Peck 1975: 346).

**Holotype.** Not designated (see Remarks).

**Type locality.** Co-operative Republic of Guyana, Demerara-Haimaca, eastern bank of the river Demerara.

**Language of species description.** English.

**Remarks.** Additional historical and morphological details of this species have been published after the original description (Bouvier 1905: 208). Evans used the terms ‘male type specimen’ and ‘female type specimen’ in his figure legends (Evans 1903: 159–160), but since a holotype has not been designated explicitly, these specimens should be regarded as syntypes. Bouvier (1907a: 518) refers to a cotype [obsolete for syntype] deposited in the Natural History Museum of London, UK. The collecting data provided by Evans (1903: 148) and Bouvier (1905: 209), as well as in the label accompanying the syntype, are imprecise: the Demerara River extends for more than 345 km. Hence, *Macroperipatusguianensis* is regarded herein as a nomen dubium since precise locality data are missing in the literature, thus precluding an unambiguous revision of this species based on topotypes.

**nd7. *Macroperipatusperrieri* (Bouvier, 1899d)**

**Synonyms.***Peripatusperrieri*, as originally described (Bouvier 1899d: 1345); Peripatus (Macroperipatus) perrieri (Clark 1913a: 17); *Macroperipatusperrieri* (Peck 1975: 347).

**Holotype.** Deposited in the Museum National d’Histoire Naturelle de Paris, France (see Remarks).

**Type locality.** Mexico, Veracruz (see Remarks)

**Language of species description.** French.

**Remarks.** Additional historical and morphological details of this species have been published after the original description (Bouvier 1905: 195). The holotype has not been designated explicitly in the original description. Since *Macroperipatusperrieri* has been described based on a single specimen (Bouvier 1899c: 440), the ‘type’ deposited in the Museum National d’Histoire Naturelle de Paris, France (see Bouvier 1907a: 518; Le Bras et al. 2015) should be regarded as holotype fixed by monotypy, following the ICZN (Art. 73.1.2). The description contains imprecise type locality data: Veracruz (originally spelt Vera-Cruz) may either refer to the Mexican State of Veracruz, which covers 71,826 km^2^, or most likely the port city of Veracruz, which currently covers an area of 1,642 km^2^. Even assuming that the latter represents the type locality of *Macroperipatusperrieri*, it is possible that forested areas putatively inhabited by this species no longer exist due to urbanization. Hence, *Macroperipatusperrieri* is regarded herein as a nomen dubium since more precise locality data are unavailable and an unambiguous revision of this species based on topotypes will be difficult.

##### VI. *Mesoperipatus* Evans, 1901a

**Type species.***Mesoperipatustholloni* (Bouvier, 1898a), by monotypy (Evans 1901a: 479).

**Remarks.** An emended diagnosis has been provided for this genus (Oliveira et al. 2012b: 18; Costa and Giribet 2016: 4).

**41. *Mesoperipatustholloni* (Bouvier, 1898a)**

**Synonyms.***Peripatustholloni*, as originally described (Bouvier 1898a: 1359); *Mesoperipatustholloni* (Evans 1901a: 478).

**Holotype.** Not designated (see Remarks).

**Type locality.** Gabon, possibly Ngolé, on the river Ogowe (see Remarks). Originally described as “Gabon” (Bouvier 1898a: 1359).

**Language of species description.** French. English translation available (Bouvier 1898b).

**Remarks.** Additional historical and morphological details of this species have been published after the original description (Bouvier 1905: 337). The holotype has not been designated explicitly in the original description. The ‘two female types’ mentioned by Bouvier (1905: 339) should be regarded as syntypes. At least one syntype should be deposited in the Museum National d’Histoire Naturelle de Paris, France (Bouvier 1907a: 519), although this specimen has not been listed by Le Bras et al. (2015). The original description contains imprecise type locality data: Gabon covers an area of 267,667 km^2^. Bouvier (1905: 348) refers to additional specimens found in Ngolé (spelt ‘Ngômô’), along the river Ogowe (spelt ‘Ogôoué’) and suggests this as a more precise locality for the species, since the syntype label only says Gabon (a common practice at that time according to Bouvier 1905: 348). Additional specimens of this species have been collected in Ndjolé, along the same river (Bouvier 1907a: 519) and in the environs of Lambaréné (0°39.49'S, 10°11.95'E, 152 m), Moyen-Ogooué Province, Gabon (Costa and Giribet 2016: 4). *Mesoperipatustholloni* has recently been re-described by Costa and Giribet (2016). Given the wide distribution of this species, the existence of a species complex within *Mesoperipatustholloni* cannot be excluded and further revision is still required. For the sake of caution, the name *Mesoperipatustholloni* should only be applied to specimens from the type locality. Molecular data have recently become available for this species (e.g., Murienne et al. 2014; Giribet et al. 2018).

##### VII. *Mongeperipatus* Barquero-González, Sánchez-Vargas & Morera-Brenes, 2020

**Type species.***Mongeperipatuskekoldi* Barquero-González, Sánchez-Vargas & Morera-Brenes, 2020 by original designation (Barquero-González et al. 2020: 306).

**Remarks.** Genus raised based on morphological and molecular data.

**42. *Mongeperipatuskekoldi* Barquero-González, Sánchez-Vargas & Morera-Brenes, 2020**

**Synonyms.** None.

**Holotype.** Deposited in the Museo de Zoología de la Universidad de Costa Rica, San José, Costa Rica.

**Type locality.** Costa Rica, Talamanca, Reserva Indígena Keköldi, 9°37.96'N, 82°43.31'W, ca 40 m ASL (see Remarks).

**Language of species description.** English.

**Remarks.** Specific epithet originally spelt *keköldi* (Barquero-González et al. 2020: 307). According to the ICZN (Art. 32.5.2.1), however, “(i)n the case of a diacritic or other mark, the mark concerned is (to be) deleted, except that in a name published before 1985 and based upon a German word, …”. Therefore, the species name is herein corrected as *Mongeperipatuskekoldi*. Geographic coordinates provided by J.P. Barquero-González (in litt.); altitude data obtained from Google Earth based on the given coordinates. Species described based on molecular and morphological data (Barquero-González et al. 2020).

**43. *Mongeperipatussolorzanoi* (Morera-Brenes & Monge-Nájera, 2010)**

**Synonyms.***Peripatussolorzanoi*, as originally described (Morera-Brenes and Monge-Nájera 2010: 1128); *Mongeperipatussolorzanoi* (Barquero-González et al. 2020: 312); *Epiperipatussolorzanoi* (Costa et al. 2021: 790) (see Remarks).

**Holotype.** Deposited in the Museo de Zoología de la Universidad de Costa Rica, San José, Costa Rica.

**Type locality.** Costa Rica, Limón, Siquirres, Guayacán de Siquirres, 10°02.97'N, 83°32.52'W, ca 400–500 m ASL.

**Language of species description.** English.

**Remarks.** Species recently classified within *Epiperipatus* sensu lato by Costa et al. (2021) but treated herein under its original combination following the ICZN (Art. 23.3.6); further details provided in the Remarks for *Epiperipatus* above. *Mongeperipatussolorzanoi* is described based on molecular and morphological data (Morera-Brenes and Monge-Nájera 2010). Additional molecular data including transcriptome have recently been provided for this species (Giribet et al. 2018; Costa et al. 2021; Baker et al. 2021).

##### VIII. *Oroperipatus* Cockerell, 1908

**Type species.***Oroperipatuslankesteri* (Bouvier, 1899a), by original designation (Cockerell 1908: 620) (see Remarks).

**Remarks.** The name was initially introduced as a subgenus of *Peripatus* (see Cockerell 1908: 620) and subsequently raised to the genus level by Clark (1913a: 16). Cockerell (1908: 620) originally fixed *Oroperipatuslankesteri* as type species, thus its putative definition ‘by subsequent designation’, as inferred by Peck (1975: 347), is inappropriate. An emended diagnosis has been provided for this genus (Oliveira et al. 2012b: 18).

**44. *Oroperipatusbalzani* (Camerano, 1897)**

**Synonyms.***Peripatusbalzani*, as originally described (Camerano 1897: 14); *Oroperipatusbalzani* (Clark 1913a: 16).

**Holotype.** Not designated (see Remarks).

**Type locality.** Bolivia, Yungas, Chulumani, near Coroico, ca 1,600 m ASL.

**Language of species description.** Italian.

**Remarks.** Additional historical and morphological details of this species have been published after the original description (Bouvier 1905: 149). The holotype has not been designated explicitly in the original description. Syntypes possibly deposited in the Museo Civico di Storia Naturale Giacomo Doria (Museo Civico di Storia Naturale di Genova), Genoa, Italy (Camerano 1897: 12). Requires morphological revision and molecular characterization.

**45. *Oroperipatusbelli* (Bouvier, 1904b)**

**Synonyms.***Peripatusbelli*, as originally described (Bouvier 1904b: 56); *Oroperipatusbelli* (Clark 1913a: 16).

**Holotype.** Deposited in the Natural History Museum of London, UK (see Remarks).

**Type locality.** Ecuador, Durán, Guayas River, opposite to Guayaquil (see Remarks).

**Language of species description.** French.

**Remarks.** Additional historical and morphological details of this species have been published after the original description (Bouvier 1905: 136). The holotype has not been designated explicitly in the original description. Since *Oroperipatusbelli* has been described based on a single specimen (Bouvier 1904b: 56, 1905: 136), the ‘syntype’ deposited in the Natural History Museum of London, UK should be regarded as holotype fixed by monotypy, following the ICZN (Art. 73.1.2). Locality data obtained from the syntype label; Guayas River has been misspelt as Guayras [sic] River in the original label (Oliveira et al. 2012a: 18). Requires morphological revision and molecular characterization.

**46. *Oroperipatusbimbergi* (Fuhrmann, 1913)**

**Synonyms.***Peripatusbimbergi*, as originally described (Fuhrmann 1913: 242); *Oroperipatusbimbergi* (Clark 1915: 14).

**Holotype.** Not designated (see Remarks).

**Type locality.** Colombia, valley of the river Amagá, in the central mountain range (900–1,800 m) and in the eastern mountain range (800 m) next to Guaduas towards Bogota (see Remarks).

**Language of species description.** German. French translation available (Fuhrmann 1914).

**Remarks.** The holotype has not been designated explicitly in the original description. Thus, the ‘types’ deposited in the Natural History Museum of London, UK represent syntypes, following the ICZN (Art. 72.4). The locality name ‘Amagatal’ used by Fuhrmann (1913: 242) and previously not identified by Oliveira et al. (2012a: 19) is the German word for the valley [German = Tal] of the river Amagá. Requires morphological revision and molecular characterization.

**47. *Oroperipatusbluntschlii* Fuhrmann, 1915**

**Synonyms.** None (see Remarks).

**Holotype.** Probably deposited in the Senckenberg Research Institute and Natural History Museum, Frankfurt, Germany (see Remarks).

**Type locality.** Peru, Loreto, Shapajilla, Samiria River, ca 120 m ASL.

**Language of species description.** German.

**Remarks.**Fuhrmann (1915: 35) uses both spellings *Oroperipatusbluntschlii* and *Peripatusbluntschlii* for referring to the same species in the original description. The holotype has not been designated explicitly in the original description. *Oroperipatusbluntschlii* has been described based on a single specimen (Fuhrmann 1915: 277), which should be regarded as the holotype fixed by monotypy, following the ICZN (Art. 73.1.2). The location of the holotype is assumed based on the indication that the only specimen studied was provided by the ‘Senckenberg Museum’ (Fuhrmann 1915: 277), which at that time corresponded to the Senckenberg Museum Frankfurt, Germany. Requires morphological revision and molecular characterization.

**48. *Oroperipatuscameranoi* (Bouvier, 1899a)**

**Synonyms.***Peripatusquitensis* (Camerano 1898: 308); *Peripatuscameranoi*, as originally described (Bouvier 1899a: 1030); *Oroperipatuscameranoi* (Clark 1913a: 16).

**Holotype.** Not designated (see Remarks).

**Type locality.** Ecuador, Azuay, Sigsig, southeast of Cuenca, ca 2,550 m ASL.

**Language of species description.** French.

**Remarks.** Additional historical and morphological details of this species have been published after the original description (Bouvier 1905: 113). The holotype has not been designated explicitly in the original description. Requires morphological revision and molecular characterization.

**49. *Oroperipatuscorradi* (Camerano, 1898)**

**Synonyms.***Peripatuscorradi*, as originally described (Camerano 1898: 310); *Peripatuscorradoi* (Bouvier 1905: 20); *Oroperipatuscorradoi* (Clark 1913a: 16) (see Remarks).

**Holotype.** Not designated (see Remarks).

**Type locality.** Ecuador, Pichincha, environs of Quito (see Remarks).

**Language of species description.** Italian.

**Remarks.** The emendation of the specific epithet as ‘*corradoi*’ suggested by Oliveira et al. (2012a: 19) is incorrect: Camerano (1898: 310) latinized ‘Corrado’ as ‘Corradus’, the correct genitive form of which is ‘corradi’. Additional historical and morphological details of this species have been published after the original description (Bouvier 1905: 120). The holotype has not been designated explicitly in the original description. Bouvier (1907a: 518) refers to a cotype [obsolete for syntype] deposited in the Museum of Turin, Italy. The description contains imprecise locality data and the redescription of the species (Bouvier 1905: 120) was based on specimens from different localities. Hence, the existence of a species complex within *Oroperipatuscorradi* cannot be excluded. Molecular data recently assigned to *Oroperipatuscorradi* (see Costa et al. 2021) may not strictly correspond to the original species, as the sequenced specimens were collected 275–427 km away from the type locality. Requires revision.

**50. *Oroperipatusecuadorensis* (Bouvier, 1902c)**

**Synonyms.***Peripatusecuadorensis*, as originally described (Bouvier 1902c: 53); *Oroperipatusequadoriensis* (Clark 1913a: 16) (see Remarks).

**Holotype.** Deposited in the Museum National d’Histoire Naturelle de Paris, France.

**Type locality.** Ecuador, Púlun (previously Bulim), northwestern Ecuador, Pacific side of the Andes, ca 20 m [60 ft] ASL.

**Language of species description.** French.

**Remarks.** The species name is commonly misspelt as *equadoriensis* (e.g., Clark 1913a: 16) or *ecuadoriensis* (e.g., Clark 1915: 25; Peck 1975: 347). Additional historical and morphological details of this species have been published after the original description (Bouvier 1905: 80). Requires morphological revision and molecular characterization.

**51. *Oroperipatuseisenii* (Wheeler, 1898)**

**Synonyms.***Peripatuseisenii*, as originally described (Wheeler 1898: 1); *Oroperipatuseiseni* (Clark 1913a: 16) (see Remarks).

**Holotype.** Not designated (see Remarks).

**Type locality.** Mexico, Nayarit, outskirts of Tepic, ca 1,220 m [4,000 ft] ASL.

**Language of species description.** English.

**Remarks.** The species name is commonly misspelt as *eiseni* (e.g., Clark 1913a: 16; Vasconcellos et al. 2004: 140). Additional historical and morphological details of this species have been published after the original description (Bouvier 1905: 128). The holotype has not been designated explicitly in the original description. A syntype is deposited in the Museum of Comparative Zoology at Harvard University, Cambridge, USA. Bouvier (1907a: 518) also refers to a cotype [obsolete for syntype] deposited in the Museum National d’Histoire Naturelle de Paris, France (see also Le Bras et al. 2015). *Oroperipatuseisenii* has recently been redescribed based morphological and molecular data (Cupul-Magaña and Navarrete-Heredia 2008; Contreras-Félix et al. 2018) but specimens analyzed were obtained from different localities. While the great distance among collecting sites (up to 100km) does not allow excluding the existence of a species complex within *Oroperipatuseisenii*, specimens found outside the El Naranjo Cave (21°27.2'N, 105°0'W, 361 m) and Tetepozco River (21°27.53'N, 105°0'W, 362 m), in Jalcocotán could possibly represent the original species, given the proximity of these records to the type locality (Contreras-Félix et al. 2018). Additional molecular data including transcriptome have recently been provided for this species (Giribet et al. 2018; Baker et al. 2021).

**52. *Oroperipatusintermedius* (Bouvier, 1901a)**

**Synonyms.***Peripatusintermedius*, as originally described (Bouvier 1901a: 168); *Oroperipatusintermedius* (Clark 1913a: 16).

**Holotype.** Deposited in the Natural History Museum of Lübeck, Lübeck, Germany.

**Type locality.** Bolivia, La Paz, Sorata (see Remarks).

**Language of species description.** French.

**Remarks.** Same locality data has been provided for *Oroperipatussoratanus* (Bouvier 1901a: 168). Additional historical and morphological details of this species have been published after the original description (Bouvier 1905: 154). Requires morphological revision and molecular characterization.

**53. *Oroperipatuskoepckei* Zilch, 1954b**

**Synonyms.** None.

**Holotype.** Deposited in the Senckenberg Research Institute and Natural History Museum, Frankfurt, Germany (see Remarks).

**Type locality.** Peru, possibly Lambayeque, between Chochopé and Uyurpampa (see Remarks). Originally described as “Peru, western side of the Andes, next to kilometer 35 of the road from Olmos to Jaén, approximately 6°10'S, 79°30'W. Sparse mountain forest, approximately 1,400 m” (Zilch 1954b: 153).

**Language of species description.** German.

**Remarks.** The location of the holotype is assumed based on the acronym SMF used by the author, possibly referring to his work institution [Senckenberg Museum Frankfurt] at that time (Zilch 1954b: 151). The type locality has mistakenly been assigned to the province of Piura by Oliveira et al. (2012a: 20), while the given geographic coordinates indicate an area located in the Lambayeque province. Requires morphological revision and molecular characterization.

**54. *Oroperipatuslankesteri* (Bouvier, 1899a)**

**Synonyms.***Peripatuslankesteri*, as originally described (Bouvier 1899a: 1030); *Oroperipatuslankesteri* (Clark 1913a: 16).

**Holotype.** Deposited in the Natural History Museum of London, UK (see Remarks).

**Type locality.** Ecuador, possibly Imbabura, River Parambas, 16 km [10 miles] north of Quito (see Remarks). Originally described as “environs of Quito” (Bouvier 1899a: 1030).

**Language of species description.** French.

**Remarks.** Additional historical and morphological details of this species have been published after the original description (Bouvier 1905: 90). The holotype has not been designated explicitly in the original description. Since *Oroperipatuslankesteri* has been described based on a single specimen (Bouvier 1905: 91), the ‘syntype’ deposited in the Natural History Museum of London, UK should be regarded as holotype fixed by monotypy, following the ICZN (Art. 73.1.2). According to Oliveira et al. (2012a: 21), a more precise locality has been obtained from the syntype label. Requires morphological revision and molecular characterization.

**55. *Oroperipatusmultipodes* (Fuhrmann, 1913)**

**Synonyms.***Peripatusmultipodes*, as originally described (Fuhrmann 1913: 244); *Oroperipatusmultipodes* (Clark 1915: 25).

**Holotype.** Deposited in the Natural History Museum of London, UK (see Remarks).

**Type locality.** Colombia, Antioquia, Concordia, river Amagá (see Remarks).

**Language of species description.** German. French translation available (Fuhrmann 1914).

**Remarks.** The name of the river has been misspelt as Rio Amago [sic] in the original description (Fuhrmann 1913: 244). The holotype has not been designated explicitly in the original description. Since *Oroperipatusmultipodes* has been described based on single specimen (Fuhrmann 1913: 244), the ‘syntype’ deposited in the Natural History Museum of London, UK, should be regarded as holotype fixed by monotypy, following the ICZN (Art. 73.1.2). Requires morphological revision and molecular characterization.

**56. *Oroperipatusomeyrus* du Bois-Reymond Marcus, 1952**

**Synonyms.** None.

**Holotype.** Not designated (see Remarks).

**Type locality.** Peru, Cusco, Sahuayaco in the Urubamba Valley (between Abancay and Maras), 800 m and San José de Lourdes (Cajamarca), ca 1,000 m ASL (see Remarks).

**Language of species description.** English.

**Remarks.** The author’s name is often cited incompletely as ‘Marcus’, although in this case, the correct form of her name is ‘du Bois-Reymond Marcus’. The holotype has not been designated explicitly in the original description. Most specimens (syntypes) investigated in the original description come from Urubamba Valley, which is regarded herein as type locality. The author also considered one specimen (syntype) found in San José de Lourdes as belonging to the same species. Since these localities lie 1084 km apart from each other, the existence of a species complex within *Oroperipatusomeyrus* cannot be excluded. For the sake of caution, the name *Oroperipatusomeyrus* should only be applied to specimens from the main locality. Syntypes can possibly be deposited in the Museu de Zoologia da Universidade de São Paulo, São Paulo, Brazil, the author’s working institution at that time [not verified]. Requires morphological revision and molecular characterization.

**57. *Oroperipatusperuvianus* (Brues, 1917)**

**Synonyms.**Peripatus (Oroperipatus) peruvianus, as originally described (Brues 1917: 383); *Oroperipatusperuvianus* (du Bois-Reymond Marcus 1952: 191).

**Holotype.** Deposited in the Museum of Comparative Zoology at Harvard University, Cambridge, USA.

**Type locality.** Peru, Cajamarca, Tabaconas, near Huancabamba, ca 1,830 m [6,000 ft] ASL.

**Language of species description.** English.

**Remarks.** Requires morphological revision and molecular characterization.

**58. *Oroperipatussoratanus* (Bouvier, 1901a)**

**Synonyms.***Peripatussoratanus*, as originally described (Bouvier 1901a: 168); *Oroperipatussoratanus* (Clark 1913a: 16).

**Holotype.** Deposited in the Natural History Museum of Lübeck, Lübeck, Germany (see Remarks).

**Type locality.** Bolivia, La Paz, Sorata.

**Language of species description.** French.

**Remarks.** Additional historical and morphological details of this species have been published after the original description (Bouvier 1905: 143). The holotype has not been designated explicitly in the original description. Since *Oroperipatussoratanus* has been described based on a single specimen (Bouvier 1901a: 168), the specimen deposited in the Deposited in the Natural History Museum of Lübeck, Lübeck, Germany should be regarded as holotype fixed by monotypy, following the ICZN (Art. 73.1.2). Same locality data has been provided for *Oroperipatusintermedius* (Bouvier 1901a: 168). Requires morphological revision and molecular characterization.

**59. *Oroperipatustuberculatus* (Bouvier, 1898c)**

**Synonyms.***Peripatustuberculatus*, as originally described (Bouvier 1898c: 1525); *Oroperipatustuberculatus* (Clark 1913a: 16).

**Holotype.** Deposited in the Museum National d’Histoire Naturelle de Paris, France (see Remarks).

**Type locality.** Colombia, Cauca, Popayán.

**Language of species description.** French. English translation available (Bouvier 1898d).

**Remarks.** Additional historical and morphological details of this species have been published after the original description (Bouvier 1905: 100). The holotype has not been designated explicitly in the original description. Since *Oroperipatustuberculatus* has been described based on a single specimen (Bouvier 1898c: 1525), the ‘type’ deposited in the Museum National d’Histoire Naturelle de Paris, France (see Bouvier 1907a: 519; Le Bras et al. 2015) should be regarded as holotype fixed by monotypy, following the ICZN (Art. 73.1.2). Requires morphological revision and molecular characterization.

**60. *Oroperipatusweyrauchi* du Bois-Reymond Marcus, 1952**

**Synonyms.** None.

**Holotype.** Not designated (see Remarks).

**Type locality.** Peru, Yurac, river Aguaytía, west affluent of the Ucayali, ca 300 m ASL.

**Language of species description.** English.

**Remarks.** The author’s name is often cited incompletely as ‘Marcus’, although in this case, the correct form is ‘du Bois-Reymond Marcus’. The holotype has not been designated explicitly in the original description. Syntypes can possibly be deposited in the Museu de Zoologia da Universidade de São Paulo, São Paulo, Brazil, the author’s working institution at that time [not verified]. Requires morphological revision and molecular characterization.

**Nomina dubia**

**nd8. *Oroperipatusgoudoti* (Bouvier, 1899d)**

**Synonyms.***Peripatusgoudoti*, as originally described (Bouvier 1899d: 1345); *Oroperipatusgoudoti* (Clark 1913a: 16).

**Holotype.** Deposited in the Museum National d’Histoire Naturelle de Paris, France.

**Type locality.** Unknown (see Remarks).

**Language of species description.** French.

**Remarks.** Additional historical and morphological details of this species have been published after the original description (Bouvier 1905: 139). The holotype has not been designated explicitly in the original description. Since *Oroperipatusgoudoti* has been described based on a single specimen (see Bouvier 1905: 140), the ‘type’ deposited in the Museum National d’Histoire Naturelle de Paris, France (Bouvier 1907a: 518; Le Bras et al. 2015) should be regarded as holotype fixed by monotypy, following the ICZN (Art. 73.1.2). The collecting data of *Oroperipatusgoudoti* are surrounded by speculations. Bouvier (1905: 143) states that [loose English translation] “(t)his species was found in Mexico by Goudot, in 1842. The label of the jar does not give more precise indications, but this peripatid being Andicole^1^, there is every reason to believe that it comes from the Mexican area, the waters of which flow into the Pacific Ocean”. However, historical data kindly provided by Dominique Malécot (Jura Emulation Society; René Remond Conservation and Study Center Lons-le-Saunier) indicate that the type locality, collector, and date assigned to *Oroperipatusgoudoti* might be wrong. Regarding the type locality, there is no evidence that any of the three Goudot brothers has ever been to Mexico. Etienne Goudot (a pharmacologist) and Justin Marie Goudot (a naturalist) have been to New Grenada [currently Colombia, Ecuador, Panama, and Venezuela], while Jules Prosper Goudot (a naturalist) travelled eastwards to Île Bourbon [currently Réunion] and Madagascar. Here, it is possible that ‘Mexico’ in the label of *Oroperipatusgoudoti* did not refer to the country but rather to a homonymous area located in Tunjuelito, Bogota, Colombia. Similar confusion is observed for herbarium plates of *Oritrophiumperuvianum* attributed to ‘J. Goudot’ and deposited in the Museum National d’Histoire Naturelle de Paris, France (MNHN; P-P00571345 and P-P00571350), which have originally been assigned to ‘Toluca, Mexico’ but re-assigned to Colombia. ‘Toluca’ (a city in Mexico), in this case, could represent a misinterpretation of the name ‘Tolima’ (a Department in Colombia) – an area well sampled by Justin Marie Goudot (see MNHN online database). Regarding the collector, the syntype of *Oroperipatusgoudoti* (MNHN-MY-MY115) is assigned to Jules Prosper Goudot, although he, in contrast to his brother Justin Marie, has never collected within the geographical range of Peripatidae. I believe that, in this case, the names have simply been mixed up: the label ‘J. Goudot’ had previously been used without distinguishing between Jules Prosper and Justin Marie (see MNHN online database), and since Jules Prosper had previously reported an onychophoran from the Table Mountains in South Africa (Gervais 1837: 38), his name could have automatically been assigned to *Oroperipatusgoudoti* without further verification. Regarding the date, 1842 attributed to *Oroperipatusgoudoti* may not represent the collecting year, as at that time, Justin Marie Goudot had just returned to France bringing part of the material collected in ‘New Greanada’, whereas Jules Prosper was still residing in Madagascar. Since several Colombian specimens collected by ‘J. Goudot’ are assigned to a very limited period (1842–1844; see MNHN online database), the year here may rather represent the time, in which the material arrived in Europe. Despite speculative, it is thus possible that *Oroperipatusgoudoti* has been collected by Justin Marie Goudot in the environs of Bogota, Colombia during his stay in ‘New Grenada’ (1822–1842). However, these issues cannot be solved unambiguously, and since no further work with more precise collecting data is available for *Oroperipatusgoudoti*, a revision of this species based on topotypes will be difficult.

**nd9. *Oroperipatusquitensis* (Schmarda, 1871)**

**Synonyms.***Peripatusquitensis*, as originally described (Schmarda 1871: 371); *Oroperipatusquitensis* (Clark 1913a: 16).

**Holotype.** Not designated (see Remarks).

**Type locality.** Ecuador, equatorial highlands of South America (see Remarks).

**Language of species description.** German.

**Remarks.** Additional historical and morphological details of this species have been published after the original description (Bouvier 1905: 109). Even though locality data for this species appear in Schmarda’s book (1871: 134), the species name is only mentioned as a figure legend on page 371 of the same publication. The citation of *Oroperipatusquitensis* as ‘a forgotten species of *Peripatus*’ by Bell (1887) refers to a later edition of Schmarda’s book ‘Zoologie’ published in 1878. Although the name *quitensis* suggests that the species was found in the environs of Quito, Ecuador, neither type specimens nor the type locality are known for this species. The species regarded as *Oroperipatusquitensis* by Camerano (1898: 308) rather corresponds to *Oroperipatuscameranoi* (Bouvier, 1899a). Thus, Oliveira et al. (2012a: 23) regarded this species as nomen dubium since type material is unknown and precise locality data are missing in the literature, thus precluding an unambiguous revision of this species based on topotypes.

**nd10. *Oroperipatusperuanus* (Grube, 1876)**

**Synonyms.***Peripatusperuanus*, as originally described (Grube 1876: 72); *Oroperipatusperuanus* (Peck 1975: 348).

**Holotype.** Not designated (see Remarks).

**Type locality.** Peru.

**Language of species description.** German.

**Remarks.**Bouvier (1905: 74) considered the classification of this species within the ‘Péripates andicoles’ group [= *Oroperipatus*] as incertae sedis. The holotype has not been designated explicitly in the original description. Oliveira et al. (2012a: 23) classified *Oroperipatusperuanus* as a nomen dubium due to overall lack of information on this species, including morphological data, designation and deposit of type specimens, and precise type locality. *Oroperipatusperuanus* has also been regarded as doubtful by several authors (e.g., Bouvier 1905: 74, 1907b: 300; du Bois-Reymond Marcus 1952: 192; Zilch 1954b: 151).

##### IX. *Peripatus* Guilding, 1826

**Type species.***Peripatusjuliformis* Guilding, 1826, by monotypy (Guilding 1826: 444).

**Remarks.***Peripatus* is the oldest genus of Onychophora and for many years, it enclosed all velvet worm species described. As species were gradually classified into different taxa, *Peripatus* became restricted to a small set Neotropical peripatids. Yet, the vernacular name ‘peripatus’ is sometimes still used as a synonym of velvet worms (e.g., Walker 1986; Barrett 1938; Briscoe and Tait 1993). Some authors refer to this genus as *Peripatus* sensu stricto (e.g., Clark 1913a: 17; Froehlich 1962: 325) to avoid confusion with the obsolete, broad use of the name. An emended diagnosis has been provided for this genus (Oliveira et al. 2012b: 18), although proper morphological and molecular characterization is still required.

**61. *Peripatusbasilensis* Brues, 1935**

**Synonyms.**Peripatusdominicaevar.basilensis, as originally described (Brues 1935: 62); *Peripatusdominicaebasilensis* (Peck 1975: 348); *Peripatusbasilensis* (Oliveira et al. 2012a: 23) (see Remarks).

**Holotype.** Probably deposited in the Museum of Comparative Zoology at Harvard University, Cambridge, USA (see Remarks).

**Type locality.** Haiti, Morne Basile (Mount Basil), northwestern part of the island, approximately ca 1,220 m [4,000 ft] ASL (see Remarks).

**Language of species description.** English.

**Remarks.** The holotype has not been designated explicitly in the original description. *Peripatusbasilensis* has been described based on a single specimen (Brues 1935: 62), which should be regarded as holotype fixed by monotypy, following the ICZN (Art. 73.1.2). According to Brues (1935: 62), the only specimen studied was a female with 28 leg pairs collected by Dr. P.J. Darlington in 1934 at Mount Basil and deposited in the Museum of Comparative Zoology at Harvard University, USA. A specimen deposited in this collection under the name *Peripatushaitiensis* (MCZ:IZ:83621) matches the morphological and collecting data provided by Brues (1935: 62) and possibly correspond to the material originally studied, i.e., the holotype of *Peripatusbasilensis*. The name ‘var. basilensis’ (Brues 1935: 62) was most likely deemed to be subspecific, as the author did not expressly give it infrasubspecific rank (see ICZN Art. 45.6.4). Oliveira et al. (2012a: 23), however, regarded *Peripatusbasilensis*, as well as the other subspecies of *Peripatusdominicae*, as separate species based on the great distances between type localities (ranging from 115 km to 1,380 km). This species has subsequently been recorded from different localities (Brues 1939: 36), hence the existence of a species complex within *Peripatusbasilensis* cannot be excluded. For the sake of caution, the name *Peripatusbasilensis* should only be applied to specimens from the type locality. Molecular data recently assigned to *Peripatusbasilensis* (see Murienne et al. 2014; Giribet et al. 2018) may not strictly correspond to the original species, as the sequenced specimen was collected in the Dominican Republic, more than 340 km away from the type locality. Requires revision.

**62. *Peripatusbouvieri* Fuhrmann, 1913**

**Synonyms.***Epiperipatusbouvieri* (Costa et al. 2021: 790) (see Remarks).

**Holotype.** Not designated (see Remarks).

**Type locality.** Colombia, Boca del Monte, at the border between Casanare and Arauca.

**Language of species description.** German. French translation available (Fuhrmann 1914).

**Remarks.** Note that the name *bouvieri* has previously been used by Cockerell (1901: 326) for *Peripatusjamaicensis* mut. *bouvieri* (see *Plicatoperipatusjamaicensis* below). The holotype has not been designated explicitly in the original description. A syntype is deposited in the Natural History Museum of London, UK. Species recently classified within *Epiperipatus* sensu lato by Costa et al. (2021) but treated herein under its original combination following the ICZN (Art. 23.3.6); further details provided in the Remarks for *Epiperipatus* above. Molecular data recently assigned to *Peripatusbouvieri* (see Costa et al. 2021) may not strictly correspond to the original species, as the sequenced specimens were collected more than 313 km away from the type locality. The geographic coordinates provided for *Peripatusbouvieri* in ‘table 1’ of Costa et al. (2021: 765) may contain a typo (missing the minus sign associated with longitude). Requires revision.

**63. *Peripatusbroelemanni* Bouvier, 1899d**

**Synonyms.** None.

**Holotype.** Not designated (see Remarks). Lectotype deposited in the Museum National d’Histoire Naturelle de Paris, France.

**Type locality.** Venezuela, Mérida, Tovar.

**Language of species description.** French.

**Remarks.** Additional historical and morphological details of this species have been published after the original description (Bouvier 1905: 246). The holotype has not been designated explicitly in the original description. Bouvier (1905: 248) designates a male deposited in the Museum National d’Histoire Naturelle de Paris, France (see Bouvier 1907a: 519; Le Bras et al. 2015) as being the ‘type’ of *Peripatusbroelemanni*. This should be regarded as the lectotype following the ICZN (Art. 74.5). The specific epithet has originally been spelt as ‘*brölemanni*’ (Bouvier 1899d: 1345) but emended to ‘*broelemanni*’ by Oliveira et al. (2012a: 24), considering that “… a name published before 1985 and based upon a German word, the umlaut sign is deleted from a vowel and the letter ‘e’ is to be inserted after that vowel” (see ICZN Art. 32.5.2.1). The species name is commonly misspelt as *brolemanni* (e.g., Peck 1975: 348). Requires morphological revision and molecular characterization.

**64. *Peripatusdanicus* Bouvier, 1900c**

**Synonyms.***Peripatusjuliformisdanicus*, as originally described (Bouvier 1900c: 751); *Peripatusdanicus* (Clark 1913a: 17) (see Remarks).

**Holotype.** Not designated (see Remarks). Lectotype deposited in the Natural History Museum of Denmark [Museum of Copenhagen], Copenhagen Denmark.

**Type locality.** Virgin Islands, Saint Thomas Island (see Remarks).

**Language of species description.** French.

**Remarks.** Additional historical and morphological details of this species have been published after the original description (Bouvier 1905: 245). The holotype has not been designated explicitly in the original description. The species has originally been described based on a syntype series of three specimens (Bouvier 1900c: 751, 752). Bouvier (1905: 245) has subsequently designated a female deposited in the Museum of Copenhagen, Denmark [currently Natural History Museum of Denmark] as being the ‘type’ of *Peripatusdanicus*. According to the ICZN (Art. 74.5), this specimen constitutes the lectotype of this species. Although Clark (1913a: 17) raised the subspecies danicus to species level, Peck (1975: 348) kept its subspecific rank, as originally suggested by Bouvier (1900c: 751). The description contains imprecise type locality data: Saint Thomas covers an area of 81 km^2^. Since the island is separated from other islands and from the mainland by seawater, Oliveira et al. (2012a: 24) regarded *Peripatusdanicus* as a valid species, following Clark (1913a). However, Oliveira et al. (2012a: 24) did not rule out the possible existence of additional species on the same island. Requires morphological revision and molecular characterization, particularly including specimens from different localities of the island.

**65. *Peripatusdarlingtoni* Brues, 1935**

**Synonyms.**Peripatusdominicaevar.darlingtoni, as originally described (Brues 1935: 62); *Peripatusdominicaedarlingtoni* (Peck 1975: 348); *Peripatusdarlingtoni* (Oliveira et al. 2012a: 24) (see Remarks).

**Holotype.** Not designated (see Remarks).

**Type locality.** Haiti, Massif (Plateau) de la Hotte, southwestern peninsula of Haiti, between Camp Perrion and Mafin, ca 915 m [3,000 ft] ASL.

**Language of species description.** English.

**Remarks.** The holotype has not been designated explicitly in the original description. The name ‘var. darlingtoni’ (Brues 1935: 62) was most likely deemed to be subspecific, as the author did not expressly give it infrasubspecific rank (see ICZN Art. 45.6.4). Oliveira et al. (2012a), however, regarded *Peripatusdarlingtoni*, as well as the other subspecies of *Peripatusdominicae*, as separate species based on the great distances between type localities (ranging from 115 km to 1,380 km). The only exception is *Peripatuslachauxensis*, which occurs relatively close to *Peripatusdarlingtoni* (~ 7 km). Yet, morphological differences together with their occurrences at different altitudes (*Peripatuslachauxensis*: 305 m; *Peripatusdarlingtoni*: 914 m) and environments (Brues 1935: 61, 62) suggest non-conspecificity between these two species (see Oliveira et al. 2012a: 23). Requires morphological revision and molecular characterization.

**66. *Peripatusdominicae* Pollard, 1893**

**Synonyms.***Peripatusdominicae*, as originally described (Pollard 1893: 290); *Peripatusdominicaedominicae* (Peck 1975: 348) (see Remarks).

**Holotype.** Not designated (see Remarks).

**Type locality.** Dominica Island, Laudat (see Remarks).

**Language of species description.** English.

**Remarks.**Peck (1975: 348) introduced the name *Peripatusdominicaedominicae* for distinguishing the original species from other subspecies then assigned to *Peripatusdominicae*. Additional historical and morphological details of this species have been published after the original description (Bouvier 1905: 252). The holotype has not been designated explicitly in the original description. Bouvier (1907a: 519) refers to a cotype [obsolete for syntype] deposited in the Museum National d’Histoire Naturelle de Paris, France (see also Le Bras et al. 2015). Locality data were obtained from specimens of *Peripatusdominicae* deposited in the Natural History Museum of London, UK (Oliveira et al. 2012a: 25). Oliveira et al. (2012a) regarded all subspecies of *Peripatusdominicae* as separate species based on the great distances between type localities (ranging from 115 km to 1,380 km) and their occurrence on a distinct island. Requires morphological revision and molecular characterization.

**67. *Peripatusevelinae* Marcus, 1937**

**Synonyms.**Peripatus (Epiperipatus) evelinae, as originally described (Marcus 1937: 905); *Peripatusevelinae* (Peck 1975: 348).

**Holotype.** Not designated (See remarks). Lectotype deposited in the Museu de Zoologia da Universidade de São Paulo, São Paulo, Brazil.

**Type locality.** Brazil, Goiás, possibly the environs of Nova Roma (see Remarks). Originally described as “the area between Cana Brava [currently Minaçu] and Nova Roma, at the border between Goyaz [sic] and Minas Gerais” (Marcus, 1937: 906).

**Language of species description.** Portuguese.

**Remarks.** The holotype has not been designated explicitly in the original description. The lectotype has been designated and deposited in the Museu de Zoologia da Universidade de São Paulo, São Paulo, Brazil (Froehlich 1968: 160). The author originally used the obsolete spelling ‘Goyaz’ for referring to the State of Goiás in Brazil. The original description contains imprecise locality data: two localities provided lie 162 km apart from each other. More precise locality data are found in a subsequent redescription of this species by Froehlich (1968: 160). Requires morphological revision and molecular characterization.

**68. *Peripatushaitiensis* Brues, 1913**

**Synonyms.***Peripatusdominicaehaitiensis* (Brues 1913: 519); Peripatusdominicaevar.haitiensis (Brues 1935: 61); *Peripatushaitiensis* (Oliveira et al. 2012a: 25).

**Holotype.** Deposited in the Museum of Comparative Zoology at Harvard University, Cambridge, USA.

**Type locality.** Haiti, Massif (Plateau) de la Selle, Furcy, La Visite National Park, ca 1,525–2,135 m [5,000–7,000 ft] ASL (see Remarks).

**Language of species description.** English.

**Remarks.** The name var.haitiensis found in Brues (1935: 61) was most likely deemed to be subspecific, as the author did not expressly give it infrasubspecific rank (see ICZN Art. 45.6.4). Also, Brues (1913: 519) previously used *haitiensis* at subspecific rank. Oliveira et al. (2012a) regarded *Peripatushaitiensis*, as well as the other subspecies of *Peripatusdominicae*, as separate species based on the great distances between type localities (ranging from 115 km to 1,380 km). A more precise type locality for this species has been provided in a subsequent publication (Brues 1935: 61). Requires morphological revision and molecular characterization.

**69. *Peripatusheloisae* Carvalho, 1941**

**Synonyms.** None.

**Holotype.** Previously deposited in the Museu Nacional do Rio de Janeiro, Rio de Janeiro, Brazil (see Remarks).

**Type locality.** Brazil, Mato Grosso, possibly Santa Terezinha (See Remarks). Originally described as “left border of Tapirapé river, next to its confluence with the river Araguaia” (Carvalho, 1941: 448).

**Language of species description.** Portuguese.

**Remarks.** The holotype has confirmedly been destroyed during the fire that consumed great part of the collections held at the Museu Nacional do Rio de Janeiro in 2019 (A.B. Kury and C.S. Costa, pers. comm. 2023). Although the species name has been introduced in 1941, a comprehensive species description was only published a year later (Carvalho 1942). The river Araguaia has been misspelt as Araraguaia [sic] by Carvalho (1941: 448). According to Sampaio-Costa et al. (2009: 554), the locality described as ‘Barra do Tapirapé’ by Carvalho (1942: 66) currently corresponds to the municipality of Santa Terezinha. Requires morphological revision, molecular characterization, and neotype designation.

**70. *Peripatusjuanensis* Bouvier, 1900b**

**Synonyms.***Peripatusdominicaejuanensis*, as originally described (Bouvier 1900b: 394); *Peripatusjuanensis* (Clark 1913a: 17).

**Holotype.** Not designated (see Remarks).

**Type locality.** Puerto Rico, Utuado.

**Language of species description.** French.

**Remarks.** Additional historical and morphological details of this species have been published after the original description (Bouvier 1905: 266). The holotype has not been designated explicitly in the original description. Röhlig et al. (2010: 227) refer to ‘syntypes’ placed in the Museum für Naturkunde Berlin, Germany. Bouvier (1907a: 518) also refers to a cotype [obsolete for syntype] deposited in the Museum National d’Histoire Naturelle de Paris, France, which was donated by the Museum für Naturkunde Berlin (see also Le Bras et al. 2015). Molecular data including transcriptome recently assigned to *Peripatusjuanensis* (see Murienne et al. 2014; Giribet et al. 2018; Baker et al. 2021) may not strictly correspond to the original species, as sequenced specimens were collected up to 97 km away from the type locality. Requires revision.

**71. *Peripatusjuliformis* Guilding, 1826**

**Synonyms.** None.

**Holotype.** Not designated (see Remarks).

**Type locality.** Saint Vincent Island, possibly Mount Bonum (see Remarks). Originally described as “at the foot of the immense mountain Bon Homme” (Guilding 1826: 444).

**Language of species description.** Latin.

**Remarks.** A wrong year of description [1825] has commonly been assigned to the species name (e.g., Bouvier 1905: 161; Clark 1913a: 17). Additional historical and morphological details of this species have been published after the original description (Bouvier 1905: 223). The holotype has not been designated explicitly in the original description. The species has been redescribed by Read (1988b: 227); also according to Read (1988b: 227), the current name of the type locality might be Mount Bonum. Requires morphological revision and molecular characterization.

**72. *Peripatuslachauxensis* Brues, 1935**

**Synonyms.**Peripatusdominicaevar.lachauxensis, as originally described (Brues 1935: 61); *Peripatusdominicaelachauxensis* (Peck 1975: 348); *Peripatuslachauxensis* (Oliveira et al. 2012a: 26).

**Holotype.** Not designated (see Remarks).

**Type locality.** Haiti, southeastern foothills of the Massif (Plateau) de la Hotte, southeastern peninsula of Haiti, Étang Lachaux, ca 305 m [1,000 ft] ASL.

**Language of species description.** English.

**Remarks.** The holotype has not been designated explicitly in the original description. The name var. lachauxensis (Brues 1935: 62) was most likely deemed to be subspecific, as the author did not expressly give it infrasubspecific rank (see ICZN Art. 45.6.4). Oliveira et al. (2012a), however, regarded *Peripatuslachauxensis*, as well as the other subspecies of *Peripatusdominicae*, as separate species based on the great distances between type localities (ranging from 115 km to 1,380 km). The only exception is *Peripatusdarlingtoni*, which occurs relatively close to *Peripatuslachauxensis* (~ 7 km). Yet, morphological differences together with their occurrences at different altitudes (*Peripatuslachauxensis*: 305 m; *Peripatusdarlingtoni*: 914 m) and environments (Brues 1935: 61–62) suggest non-conspecificity between these two species (see Oliveira et al. 2012a: 23). Requires morphological revision and molecular characterization.

**73. *Peripatusmanni* Brues, 1913**

**Synonyms.** None.

**Holotype.** Deposited in the Museum of Comparative Zoology at Harvard University, Cambridge, USA.

**Type locality.** Haiti, Massif (Plateau) de la Selle, La Visite National Park, ca 1,525–2,135 m [5,000–7,000 ft] (see Remarks).

**Language of species description.** English.

**Remarks.** A more precise type locality for this species has been provided in a subsequent publication (Brues 1935: 61). Requires morphological revision and molecular characterization.

**74. *Peripatusruber* Fuhrmann, 1913**

**Synonyms.** None.

**Holotype.** Deposited in the Musée d ´Historie Naturalle de Genève, Switzerland (see Remarks).

**Type locality.** Costa Rica, San José, Goicoechea, Rancho Redondo, possibly 9°57.69'N, 83°56.93'W, ca 2,000 m ASL (see Remarks). Originally described as “Rancho Redondo, Costa Rica” (Fuhrmann 1913: 248).

**Language of species description.** German. French translation available (Fuhrmann 1914).

**Remarks.** The holotype has not been designated explicitly in the original description. Since *Peripatusruber* is described based on a single specimen (Fuhrmann 1913: 248), which is deposited in the Musée d ´Historie Naturalle de Genève, Switzerland (Fuhrmann 1914: 190), this should be regarded as holotype fixed by monotypy, following the ICZN (Art. 73.1.2). Barquero-González et al. (2016: 1407) suggested the type locality of *Peripatusruber* is likely to be in the district of Rancho Redondo, Goicoechea canton, Costa Rica. Requires morphological revision and molecular characterization.

**75. *Peripatussedgwicki* Bouvier, 1899d**

**Synonyms.** None.

**Holotype.** Not designated (see Remarks).

**Type locality.** Venezuela, Federal district, La Guaira, environs of Caracas (see Remarks).

**Language of species description.** French.

**Remarks.** Note that the generic abbreviation *P.sedgwicki* may create confusion with the peripatopsid species *Peripatopsissedgwicki* (see Oliveira et al. 2012a: 27). Additional historical and morphological details of this species have been published after the original description (Bouvier 1905: 211). The holotype has not been designated explicitly in the original description. Bouvier (1907a: 518) refers to a cotype [obsolete for syntype] deposited in the Museum National d’Histoire Naturelle de Paris, France (see also Le Bras et al. 2015). A more precise locality for this species has subsequently been provided by Bouvier (1905: 213). The species redescription (Bouvier 1905: 220), however, included specimens from different localities, thus the existence of a species complex within *Peripatussedgwicki* cannot be excluded. For the sake of caution, the name *Peripatussedgwicki* should only be applied to specimens from the type locality. Requires morphological revision and molecular characterization.

**76. *Peripatusswainsonae* Cockerell, 1893**

**Synonyms.***Peripatusjamaicensis* mut. *swainsonae*, as originally described (Cockerell 1893: 341); *Peripatusjuliformis* mut. *gossei* (Bouvier 1900c: 751); *Peripatusjuliformisgossei* (Cockerell 1901: 326); Peripatusjuliformisvar.swainsonae (Cockerell 1901: 326); *Peripatusswainsonae* (Clark 1913a: 17) (see Remarks).

**Holotype.** Not designated (see Remarks).

**Type locality.** Jamaica, Saint Thomas Parish, Bath, Beacon Hill (see Remarks).

**Language of species description.** English.

**Remarks.** The terms variety (var.) and mutation (mut.) used by Cockerell (1893, 1901) and Bouvier (1900c) were most likely deemed to be subspecific, as the authors did not expressly give them infrasubspecific rank (see ICZN Art. 45.6.4). The synonyms of *Peripatusswainsonae* may cause confusion with those of *Plicatoperipatusjamaicensis* (see Cockerell 1901 for disambiguation). Additional historical and morphological details of this species have been published after the original description (Bouvier 1905: 232). The holotype has not been designated explicitly in the original description. Bouvier (1907a: 519) refers to a cotype [obsolete for syntype] deposited in the Museum National d’Histoire Naturelle de Paris, France, which has not been listed by Le Bras et al. (2015). The locality data have been obtained from labels of syntypes deposited in the Natural History Museum of London (Oliveira et al. 2012a: 27). Requires morphological revision and molecular characterization.

**Nomina dubia**

**nd11. *Peripatusantiguensis* Bouvier, 1899d**

**Synonyms.***Peripatusantiguensis*, as originally described (Bouvier 1899d: 1345); Peripatusdominicaevar.antiguensis (Bouvier 1905: 263); *Peripatusantiguensis* (Clark 1913a: 17).

**Holotype.** Not designated (see Remarks).

**Type locality.** Antigua and Barbuda, Antigua Island, Barlar, near Warburton (see Remarks).

**Language of species description.** French.

**Remarks.** Species name has been misspelt as *antiquensis* [sic] by Bouvier (1905: 167). Additional historical and morphological details of this species have been published after the original description (Bouvier 1905: 263). The holotype has not been designated explicitly in the original description. According to Bouvier (1905: 264, 1907a: 519), the (syn)types of this species have been deposited in the Museum National d’Histoire Naturelle de Paris, France. Le Bras et al. (2015), however, only listed one ‘type’ specimen in this institution. Neither the current name nor the position of the localities provided by Bouvier (1905: 265) on the island of Antigua could be identified. Oliveira et al. (2012a: 28) regarded *Peripatusantiguensis* as a nomen dubium since precise locality data are missing in the literature (the Antigua Island covers an area of 281 km^2^), thus precluding an unambiguous revision of this species based on topotypes.

**nd12. *Peripatusbavayi* Bouvier, 1899d**

**Synonyms.**Peripatussedgwickivar.bavaysi, as originally described (Bouvier 1899d: 1346); *Peripatusbavayi* (Clark 1913a: 17) (see Remarks).

**Holotype.** Deposited in the Museum National d’Histoire Naturelle de Paris, France (see Remarks).

**Type locality.** Guadeloupe Island (see Remarks).

**Language of species description.** French.

**Remarks.** The species name has been misspelt *bavaysi* [sic] in the original species description (Bouvier 1899d: 1346). However, its correct spelling is *bavayi*, since the description (Bouvier 1899d: 1346) reads [loose English translation]: “I provisionally attach to this species [*Peripatussedgwicki*], under the name of var. bavaysi, a peripatid with an atrophied clear line, which M. Bavay brought from Guadeloupe”. Later, the same author (Bouvier 1905: 223) states [loose English translation]: “In the meantime, I have attributed to the peripatid of Guadeloupe the name of P.sedwickivar.bavayi, in honor of M. Bavay, my excellent colleague from the Zoological Society of France.”]. Hence, the specific epithet *bavaysi* (e.g., Bouvier 1899d: 1346; Oliveira et al. 2012a: 28) should be regarded as misspelling and *>bavayi* as an emendation of the species name following the ICZN (Art. 32.5.1 and 33.2). The correct spelling of the species name appears in several subsequent publications (e.g., Bouvier 1899c: 415, 1905: 222; Clark 1913a: 17; Peck 1975: 348). Additional historical and morphological details of this species have been published after the original description (Bouvier 1905: 222). The holotype has not been designated explicitly in the original description. Since *Peripatusbavayi* has been described based on a single specimen (see Bouvier 1905: 222, 223), the ‘type’ deposited in the Museum National d’Histoire Naturelle de Paris, France (see Bouvier 1907a: 518; Le Bras et al. 2015) should be regarded as holotype fixed by monotypy, following the ICZN (Art. 73.1.2). The description contains imprecise type locality data: Guadeloupe Island covers an area of 1,628 km^2^. Oliveira et al. (2012a: 28) regarded *Peripatusbavayi* as a nomen dubium since precise locality data are missing in the literature, thus precluding an unambiguous revision of this species based on topotypes.

##### X. *Plicatoperipatus* Clark, 1913a

**Type species.***Plicatoperipatusjamaicensis* (Grabham & Cockerell, 1892), by monotypy (Clark 1913a: 17).

**Remarks.**Plicatoperipatus has originally been introduced as a subgenus of Peripatus (see Clark 1913a) but it is commonly treated at the generic rank in the literature (e.g., Read 1988a: 189; Oliveira et al. 2014: 275). To prevent taxonomic instability, *Plicatoperipatus* is regarded herein as a valid genus. An emended diagnosis has been provided for this genus (Oliveira et al. 2012b: 18).

**77. *Plicatoperipatusjamaicensis* (Grabham & Cockerell, 1892)**

**Synonyms.***Peripatusjamaicensis*, as originally described (Grabham and Cockerell 1892: 514); *Peripatusjamaicensis* mut. *gossei* (Cockerell 1893: 341); *Peripatusjamaicensis* mut. *swainsonae* (Bouvier 1900c: 757); *Peripatusjamaicensis* mut. *bouvieri* (Cockerell 1901: 326); Peripatus (Plicatoperipatus) jamaicensis (Clark 1913a: 17); *Plicatoperipatusjamaicensis* (Peck 1975: 349) (see Remarks).

**Holotype.** Not designated (see Remarks).

**Type locality.** Jamaica, Saint Thomas Parish, Bath, Beacon Hill (see Remarks).

**Language of species description.** English.

**Remarks.** The term mutation (mut.) used by Cockerell (1893, 1901) and Bouvier (1900c) was most likely deemed to be subspecific, as the authors did not expressly give it infrasubspecific rank (see ICZN Art. 45.6.4). The synonyms of *Plicatoperipatusjamaicensis* may cause confusion with those of *Peripatusswainsonae* (see Cockerell 1901 for disambiguation). Also, the synonym *Peripatusjamaicensis* mut. *bouvieri* should not be confused with the valid species name *Peripatusbouvieri*. Oliveira et al. (2012a: 29) regarded both ‘mut. *bouvieri*’ and ‘mut. *gossei*’ as color variations of *Plicatoperipatusjamaicensis*, thus carrying no taxonomical meaning. Additional historical and morphological details of this species have been published after the original description (Bouvier 1905: 169). The holotype has not been designated explicitly in the original description. Bouvier (1905: 171, 1907a: 518) refers to ‘cotypes’ (obsolete for syntypes) deposited in the Museum National d’Histoire Naturelle de Paris, France, which have not been listed by Le Bras et al. (2015). The type locality data were obtained from labels of specimens deposited in the Natural History Museum of London, UK (Oliveira et al. 2012a: 29). Morphological (Oliveira et al. 2014) and molecular data (Murienne et al. 2014; Giribet et al. 2018) recently assigned to *Plicatoperipatusjamaicensis* were obtained from specimens collected 10–20 km away from the type locality. Requires morphological revision and molecular characterization.

##### XI. *Principapillatus* Oliveira, Franke, Hering, Schaffer, Rowell, Weck-Heimann, Monge-Nájera, Morera-Brenes & Mayer, 2012b

**Type species.***Principapillatushitoyensis* Oliveira, Franke, Hering, Schaffer, Rowell, Weck-Heimann, Monge-Nájera, Morera-Brenes & Mayer, 2012b, by monotypy (Oliveira et al. 2012b: 11).

**Remarks.***Principapillatus* has recently been regarded as junior synonym of *Epiperipatus* Clark, 1913a (Costa et al. 2021). However, the classification of *Epiperipatus* is still inconclusive (see Remarks for *Epiperipatus* below). For the sake of nomenclatural stability, and following the ICZN (Art. 23.3.6), *Principapillatus* is considered herein as a valid name; *Epiperipatus* as defined by Costa et al. (2021) is referred to as *sensu lato*.

**78. *Principapillatushitoyensis* Oliveira, Franke, Hering, Schaffer, Rowell, Weck-Heimann, Monge-Nájera, Morera-Brenes & Mayer, 2012b**

**Synonyms.***Epiperipatushitoyensis* (Costa et al. 2021: 790) (see Remarks).

**Holotype.** Deposited in the Museo de Zoología de la Universidad de Costa Rica, San José, Costa Rica.

**Type locality.** Costa Rica, Limón, Reserva Biológica Hitoy Cerere, 09°40.36'N, 83°02.62'W, ca 300 m ASL.

**Language of species description.** English.

**Remarks.** Species recently classified within *Epiperipatus* sensu lato by Costa et al. (2021) but treated herein under its original combination following the ICZN (Art. 23.3.6); further details provided in the Remarks for *Epiperipatus* above. Species description includes morphological, molecular and karyotype data. Slime protein profiling data provided subsequently (Baer et al. 2014).

##### XII. *Speleoperipatus* Peck, 1975

**Type species.***Speleoperipatusspelaeus* Peck, 1975, by monotypy (Peck 1975: 349).

**Remarks.** Although an emended diagnosis has been provided for this genus (Oliveira et al. 2012b: 18), *Speleoperipatus* has never been re-investigated after its description, thus requiring proper morphological and molecular characterization.

**79. *Speleoperipatusspelaeus* Peck, 1975**

**Synonyms.** None.

**Holotype.** Deposited in the Museum of Comparative Zoology at Harvard University, Cambridge, USA.

**Type locality.** Jamaica, Clarendon, Pedro River, Pedro Great Cave, ca 520 m [1,700 ft] ASL.

**Language of species description.** English.

**Remarks.** Requires morphological revision and molecular characterization.

##### XIII. *Typhloperipatus* Kemp, 1913

**Type species.***Typhloperipatuswilliamsoni* Kemp, 1913, by monotypy (Kemp 1913: 241).

**Remarks.** Although an emended diagnosis has been provided for this genus (Oliveira et al. 2012b: 18), *Typhloperipatus* has never been re-investigated after its description, thus requiring proper morphological and molecular characterization.

**80. *Typhloperipatuswilliamsoni* Kemp, 1913**

**Synonyms.** None.

**Holotype.** Not designated (see Remarks).

**Type locality.** Republic of India, Assam, Dihang River, vicinity of Rotung, ca 365–760 m [1,200–2,500 ft] ASL.

**Language of species description.** English.

**Remarks.** Although the species name has been introduced in 1913, a comprehensive species description has only been published a year later (Kemp, 1914). The holotype has not been designated explicitly in the original description. Syntypes are deposited in the Natural History Museum of London, UK. Requires morphological revision and molecular characterization.

#### ﻿Peripatopsidae Bouvier, 1905

**Type genus.***Peripatopsis* Pocock, 1894.

**Remarks.** The wrong year [1907] is commonly assigned to Peripatopsidae (e.g., Ruhberg 1985: 78), possibly referring to Bouvier’s monograph on this taxon (Bouvier 1907b). The citation ‘Peripatopsidae Bouvier, 1904c’ (e.g., Clark 1913a: 18; Reid 1996: 665) refers to Bouvier’s indication of a subfamily named ‘Péripatopsidés’ (Bouvier 1904c: 45, footnote). Yet, Peripatopsidae has only been raised explicitly as a new taxon at the family rank a year later (Bouvier 1905: 65), when the Latin term ‘nov. fam.’ has been applied to introduce the name.

##### I. *Acanthokara* Reid, 1996

**Type species.***Acanthokarakaputensis* Reid, 1996, by monotypy (Reid 1996: 716).

**Remarks.** Requires molecular characterization.

**1. *Acanthokarakaputensis* Reid, 1996**

**Synonyms.** None.

**Holotype.** Deposited in the Australian Museum, Sydney, Australia.

**Type locality.** Australia, New South Wales, Nadewar Range, Mount Kaputar, 30°16'S, 150°10'E, ca 1,500 m ASL.

**Language of species description.** English.

**Remarks.** Requires molecular characterization.

##### II. *Aethrikos* Reid, 1996

**Type species.***Aethrikossetosa* Reid, 1996, by monotypy (Reid 1996: 719).

**2. *Aethrikossetosa* Reid, 1996**

**Synonyms.** None.

**Holotype.** Deposited in the Australian Museum, Sydney, Australia.

**Type locality.** Australia, New South Wales, Styx River State Forest, 30°31'S, 152°21'E, ca 1,380 m ASL.

**Language of species description.** English.

**Remarks.** Molecular data have recently been provided for this species (Murienne et al. 2014; Giribet et al. 2018).

##### III. *Aktinothele* Reid, 1996

**Type species.***Aktinotheleeucharis* Reid, 1996, by monotypy (Reid 1996: 724).

**Remarks.** Requires molecular characterization.

**3. *Aktinotheleeucharis* Reid, 1996**

**Synonyms.** None.

**Holotype.** Deposited in the Australian National Insect Collection, Canberra, Australia.

**Type locality.** Australia, Queensland, Finch Hatton Gorge, 21°05'S, 148°38'E, ca 200 m ASL.

**Language of species description.** English.

**Remarks.** The species description includes specimens from different localities lying up to 94 km away from the type locality, hence the existence of a species complex within *Aktinotheleeucharis* cannot be excluded. For the sake of caution, the name *Aktinotheleeucharis* should only be applied to specimens from the type locality. Requires morphological revision and molecular characterization.

##### IV. *Anoplokaros* Reid, 1996

**Type species.***Anoplokaroskeerensis* Reid, 1996, by monotypy (Reid 1996: 730).

**Remarks.** Requires molecular characterization.

**4. *Anoplokaroskeerensis* Reid, 1996**

**Synonyms.** None.

**Holotype.** Deposited in the Australian Museum, Sydney, Australia.

**Type locality.** Australia, New South Wales, Mount Keira (near Scout Camp), 34°24'S, 150°50'E, ca 320 m ASL.

**Language of species description.** English.

**Remarks.** The species description includes specimens from different localities lying up to 84 km away from the type locality, hence the existence of a species complex within *Anoplokaroskeerensis* cannot be excluded. For the sake of caution, the name *Anoplokaroskeerensis* should only be applied to specimens from the type locality. Requires morphological revision and molecular characterization.

##### V. *Austroperipatus* Baehr, 1977

**Type species.***Austroperipatusparadoxus* (Bouvier, 1914a), by monotypy (Baehr 1977: 17).

**Remarks.** Requires molecular characterization.

**5. *Austroperipatusaequabilis* Reid, 1996**

**Synonyms.** None.

**Holotype.** Deposited in the Queensland Museum, Brisbane, Australia.

**Type locality.** Australia, Queensland, Mount Finnigan, 37 km south of Cooktown, 15°49'S, 145°17'E, ca 850–1,100 m ASL.

**Language of species description.** English.

**Remarks.** The species description includes specimens from different localities lying up to 95 km away from the type locality, hence the existence of a species complex within *Austroperipatusaequabilis* cannot be excluded. For the sake of caution, the name *Austroperipatusaequabilis* should only be applied to specimens from the type locality. Requires morphological revision and molecular characterization.

**6. *Austroperipatuseridelos* Reid, 1996**

**Synonyms.** None.

**Holotype.** Deposited in the Queensland Museum, Brisbane, Australia.

**Type locality.** Australia, Queensland, Boonjie, 13 km east-southeast [ESE] of Malanda, 17°24'S, 145°44'E, ca 700 m.

**Language of species description.** English.

**Remarks.** The species description includes specimens from different localities lying up to 92 km away from the type locality, hence the existence of a species complex within *Austroperipatuseridelos* cannot be excluded. For the sake of caution, the name *Austroperipatuseridelos* should only be applied to specimens from the type locality. Requires morphological revision and molecular characterization.

**7. *Austroperipatusparadoxus* (Bouvier, 1914a)**

**Synonyms.***Ooperipatusparadoxus*, as originally described (Bouvier 1914a: 1548); *Austroperipatusparadoxus* (Baehr 1977: 17).

**Holotype.** Not designated (see Remarks).

**Type locality.** Australia, Queensland, Bellenden Ker, 17°12'S, 145°51'E, ca 1,310 m [4,000 ft] ASL.

**Language of species description.** French.

**Remarks.** A wrong year of description [1915] is sometimes assigned to this species (e.g., Baehr 1977: 9). The holotype has not been designated explicitly in the original description. The holotype and paratype mentioned by Ruhberg (1985: 114, 115), and deposited in the Mjöberg collection at the Naturhistoriska Riksmuseet, Stockholm, Sweden, should rather be regarded as syntypes. These specimens have previously been cited as lectotype and paralectotypes by Reid (1996: 736) and Oliveira et al. (2012a: 31), although their designation as such does not fulfil the directives and recommendations of the ICZN (Art. 74.7). The species redescriptions (Ruhberg 1985: 114; Reid 1996: 736) include specimens from different localities lying up to 113 km away from the type locality, hence the existence of a species complex within *Austroperipatusparadoxus* cannot be excluded. For the sake of caution, the name *Austroperipatusparadoxus* should only be applied to specimens from the type locality. Requires morphological revision and molecular characterization.

**8. *Austroperipatussuperbus* Reid, 1996**

**Synonyms.** None.

**Holotype.** Deposited in the Queensland Museum, Brisbane, Australia.

**Type locality.** Australia, Queensland, Hinchinbrook Island, Gayundah Creek, 18°22'S, 146°13'E, ca 80 m ASL.

**Language of species description.** English.

**Remarks.** Requires molecular characterization.

##### VI. *Baeothele* Reid, 1996

**Type species.***Baeothelesaukros* Reid, 1996, by monotypy (Reid 1996: 749).

**Remarks.** Requires molecular characterization.

**9. *Baeothelesaukros* Reid, 1996**

**Synonyms.** None.

**Holotype.** Deposited in the Australian Museum, Sydney, Australia.

**Type locality.** Australia, New South Wales, Wollemi National Park, Mount Coricudgy, 32°50'S, 150°21'E, ca 1,350 m ASL.

**Language of species description.** English.

**Remarks.** The species description includes specimens from different localities lying up to 140 km away from the type locality, hence the existence of a species complex within *Baeothelesaukros* cannot be excluded. For the sake of caution, the name *Baeothelesaukros* should only be applied to specimens from the type locality. Requires morphological revision and molecular characterization.

##### VII. *Centorumis* Reid, 1996

**Type species.***Centorumistrigona* Reid, 1996, by monotypy (Reid 1996: 753).

**Remarks.** Requires molecular characterization.

**10. *Centorumistrigona* Reid, 1996**

**Synonyms.** None.

**Holotype.** Deposited in the Australian Museum, Sydney, Australia.

**Type locality.** Australia, New South Wales, Gloucester Tops, 32°03'S, 151°39'E, ca 700 m ASL.

**Language of species description.** English.

**Remarks.** Requires molecular characterization.

##### VIII. *Cephalofovea* Ruhberg, Tait, Briscoe & Storch, 1988

**Type species.***Cephalofoveatomahmontis* Ruhberg, Tait, Briscoe & Storch, 1988, by monotypy (Ruhberg et al. 1988: 120).

**Remarks.** Requires molecular characterization.

**11. *Cephalofoveacameroni* Reid, Tait, Briscoe & Rowell, 1995**

**Synonyms.** None.

**Holotype.** Deposited in the Australian Museum, Sydney, Australia.

**Type locality.** Australia, New South Wales, Rydal, 33°29'S, 150°02'E, ca 900 m ASL.

**Language of species description.** English.

**Remarks.** The species description includes specimens from different localities lying up to 58 km away from the type locality, hence the existence of a species complex within *Cephalofoveacameroni* cannot be excluded. For the sake of caution, the name *Cephalofoveacameroni* should only be applied to specimens from the type locality. Requires morphological revision and molecular characterization.

**12. *Cephalofoveaclandestina* Reid, Tait, Briscoe & Rowell, 1995**

**Synonyms.** None.

**Holotype.** Deposited in the Australian Museum, Sydney, Australia.

**Type locality.** Australia, New South Wales, Kanangra Boyd National Park, 33°50'S, 150°00'E, ca 1,140 m ASL.

**Language of species description.** English.

**Remarks.** Requires molecular characterization.

**13. *Cephalofoveapavimenta* Reid, Tait, Briscoe & Rowell, 1995**

**Synonyms.** None.

**Holotype.** Deposited in the Australian Museum, Sydney, Australia.

**Type locality.** Australia, New South Wales, Mount Canobolas, 33°21'S, 148°59'E, ca 1,395 m ASL.

**Language of species description.** English.

**Remarks.** Requires molecular characterization.

**14. *Cephalofoveatomahmontis* Ruhberg, Tait, Briscoe & Storch, 1988**

**Synonyms.** None.

**Holotype.** Deposited in the Australian National Insect Collection, Canberra, Australia.

**Type locality.** Australia, New South Wales, Mount Tomah, 33°33'S, 150°25'E, ca 1,015 m ASL.

**Language of species description.** English.

**Remarks.** Requires molecular characterization.

##### IX. *Critolaus* Reid, 1996

**Type species.***Critolauslepidus* Reid, 1996, by monotypy (Reid 1996: 760).

**Remarks.** Requires molecular characterization.

**15. *Critolauslepidus* Reid, 1996**

**Synonyms.** None.

**Holotype.** Deposited in the Queensland Museum, Brisbane, Australia.

**Type locality.** Australia, Queensland, Calliope Range, Kroombit Tops (south-southwest of Calliope Beauty Spot 98), 24°22'S, 150°59'E, ca 860 m ASL.

**Language of species description.** English.

**Remarks.** The species description includes specimens from different localities lying up to 47 km away from the type locality, hence the existence of a species complex within *Critolauslepidus* cannot be excluded. For the sake of caution, the name *Critolauslepidus* should only be applied to specimens from the type locality. Requires morphological revision and molecular characterization.

##### X. *Dactylothele* Reid, 1996

**Type species.***Dactylothelehabros* Reid, 1996, by monotypy (Reid 1996: 763).

**Remarks.** Requires molecular characterization.

**16. *Dactylothelehabros* Reid, 1996**

**Synonyms.** None.

**Holotype.** Deposited in the Queensland Museum, Brisbane, Australia.

**Type locality.** Australia, New South Wales, Nothofagus Mountain, 12 km north of Woodenbong, 28°17'S, 152°38'E, ca 1,200 m ASL.

**Language of species description.** English.

**Remarks.** The species description includes specimens from different localities lying up to 72 km away from the type locality, hence the existence of a species complex within *Dactylothelehabros* cannot be excluded. For the sake of caution, the name *Dactylothelehabros* should only be applied to specimens from the type locality. Requires morphological revision and molecular characterization.

##### XI. *Diemenipatus* Oliveira, Ruhberg, Rowell & Mayer, 2018

**Type species.***Diemenipatustaiti* Oliveira, Ruhberg, Rowell & Mayer, 2018, by original designation (Oliveira et al. 2018: 913).

**17. *Diemenipatusmesibovi* Oliveira, Ruhberg, Rowell & Mayer, 2018**

**Synonyms.** None.

**Holotype.** Deposited in the Queen Victoria Museum and Art Gallery, Launceston, Tasmania, Australia.

**Type locality.** Australia, Tasmania, environs of Mount King William I at the Lyell Highway (Route A10), 42°12.71'S, 146°07.26'E, ca 815 m ASL.

**Language of species description.** English.

**Remarks.** Species description includes morphological, molecular and karyotype data. Slime protein profiling data provided previously by Baer et al. (2014) under the name ‘Tasmania sp2’. Additional molecular data recently assigned to *Diemenipatusmesibovi* (see Giribet et al. 2018) may not strictly correspond to the original species, as the sequenced specimens were collected more than 82 km east from the type locality.

**18. *Diemenipatustaiti* Oliveira, Ruhberg, Rowell & Mayer, 2018**

**Synonyms.** None.

**Holotype.** Deposited in the Queen Victoria Museum and Art Gallery, Launceston, Tasmania, Australia.

**Type locality.** Australia, Tasmania, Scotts Peak Road, Lake Pedder, Huon River, 43°02.28'S, 146°18.09'E, ca 310 m ASL.

**Language of species description.** English.

**Remarks.** Species description includes morphological, molecular and karyotype data. Slime protein profiling data provided previously by Baer et al. (2014) under the name ‘Tasmania sp1’.

##### XII. *Dystactotylos* Reid, 1996

**Type species.***Dystactotylosaletes* Reid, 1996, by monotypy (Reid 1996: 768).

**Remarks.** Requires molecular characterization.

**19. *Dystactotylosaletes* Reid, 1996**

**Synonyms.** None.

**Holotype.** Deposited in the Queensland Museum, Brisbane, Australia.

**Type locality.** Australia, Massey Range, 4 km west of Centre Bellenden Ker, 17°16'S, 145°49'E, ca 1,250 m ASL.

**Language of species description.** English.

**Remarks.** The species description includes specimens from different localities lying up to 30 km away from the type locality, hence the existence of a species complex within *Dystactotylosaletes* cannot be excluded. For the sake of caution, the name *Dystactotylosaletes* should only be applied to specimens from the type locality. Requires morphological revision and molecular characterization.

##### XIII. *Euperipatoides* Ruhberg, 1985

**Type species.***Euperipatoidesleuckartii* (Sänger, 1871), by monotypy (Ruhberg 1985: 117).

**20. *Euperipatoideskanangrensis* Reid, 1996**

**Synonyms.** None.

**Holotype.** Deposited in the Australian Museum, Sydney, Australia.

**Type locality.** Australia, New South Wales, Kanangra Boyd National Park, 33°59'S, 150°08'E, ca 1,140 m ASL.

**Language of species description.** English.

**Remarks.** Molecular data including transcriptome available for this species (e.g., Murienne et al. 2014; Oliveira et al. 2018; Giribet et al. 2018; Baker et al. 2021).

**21. *Euperipatoidesleuckartii* (Sänger, 1871)**

**Synonyms.***Peripatusleuckartii*, as originally described (Sänger 1871: 257); Peripatusleuckartivar.orientalis (Fletcher 1895: 185); *Peripatusorientalis* (Bouvier 1902d: 110); *Ooperipatusleuckarti* (Bouvier 1907b: 273); Peripatoidesleuckartiivar.orientalis (Dendy 1906: 176); *Peripatoidesleuckartii* (Dendy 1906: 177); *Peripatoidesorientalis* (Clark 1914: 317); *Euperipatoidesleuckartii* (Ruhberg 1985: 118) (See Remarks).

**Holotype.** Not designated (see Remarks). Neotype deposited in the Australian Museum, Sydney, Australia.

**Type locality.** Australia, New South Wales, Mount Tomah, 33°33'S, 150°25'E, ca 1,015 m ASL.

**Language of species description.** Russian. French (Anonymous 1900) and English (Tait et al. 2022) translations available (see Remarks).

**Remarks.** See Ruhberg (1985: 118, 119) for a complete list of synonyms and synonymization. A wrong year of description [1869] has commonly been assigned to the species name (e.g., Ruhberg 1985: 117; Reid 1996: 663). According to Oliveira et al. (2012a: 34), 1869 corresponds to the year of the conference, in which the species has been presented, whereas the proceedings containing the species description has only been published two years later (Sänger 1871; Tait et al. 2022). Also, the author’s name has wrongly been cited as H. Sänger (e.g., Anonymous 1900: 9; Bouvier 1907b: 273), whereas his full name was Nikolai Karlovich Sänger and the correct citation would be N. [K.] Sänger (Tait et al. 2022). The French translation of Sänger’s work has mistakenly been credited to him (Oliveira et al. 2012a: 33) although the name of the translator remains unknown. The species name is commonly misspelt as *leuckarti* (e.g., Fletcher 1888: 892; Ruhberg 1985: 118). The holotype has not been designated explicitly in the original description. A syntype originally deposited in the zoological collection of the Institute of Biology at the University of Leipzig, Germany, was presumably lost during the II World War, when part of the scientific collection was transferred to the Staatliches Museum für Tierkunde in Dresden, Germany. A neotype has been designated by Reid (1996: 774) and is deposited in the Australian Museum, Sydney, Australia. Note that the synonym Peripatusleuckartivar.orientalis may cause confusion with Peripatusleuckartivar.occidentalis, the latter being a synonym of *Kumbadjenaoccidentalis*. Molecular data available for this species (e.g., Murienne et al. 2014; Oliveira and Mayer 2017; Oliveira et al. 2018; Giribet et al. 2018).

**22. *Euperipatoidesrowelli* Reid, 1996**

**Synonyms.** None.

**Holotype.** Deposited in the Australian Museum, Sydney, Australia.

**Type locality.** Australia, New South Wales, Tallaganda State Forest, Forbes Creek Road, 35°28'S, 149°32'E, ca 1,000 m ASL.

**Language of species description.** English.

**Remarks.** An annotated draft genome of *Euperipatoidesrowelli* has recently been provided by Thomas et al. (2020). Additional molecular data are available for this species (e.g., Bull et al. 2013; Bull and Sunnucks 2014; Murienne et al. 2014; Oliveira and Mayer 2017; Giribet et al. 2018). The species description includes specimens from different localities lying up to 102 km away from the type locality, hence the existence of a species complex within *Euperipatoidesrowelli* cannot be excluded. A putative species complex may exist even within the Tallaganda Forest (Bull et al. 2013; Bull and Sunnucks 2014). For the sake of caution, the name *Euperipatoidesrowelli* should only be applied to specimens from the type locality. Requires closer revision.

##### XIV. *Florelliceps* Tait & Norman, 2001

**Type species.***Florellicepsstutchburyae* Tait & Norman, 2001, by monotypy (Tait and Norman 2001: 303).

**Remarks.** Requires molecular characterization.

**23. *Florellicepsstutchburyae* Tait & Norman, 2001**

**Synonyms.** None.

**Holotype.** Deposited in the Australian National Insect Collection, Canberra, Australia.

**Type locality.** Australia, New South Wales, Mount Warning National Park, 28°24'S, 153°16'E, ca 400 m ASL.

**Language of species description.** English.

**Remarks.** The species description includes specimens from different localities lying up to 63 km away from the type locality, hence the existence of a species complex within *Florellicepsstutchburyae* cannot be excluded. For the sake of caution, the name *Florellicepsstutchburyae* should only be applied to specimens from the type locality. Requires morphological revision and molecular characterization.

##### XV. *Hylonomoipos* Reid, 1996

**Type species.***Hylonomoiposakares* Reid, 1996, by original designation (Reid 1996: 778).

**Remarks.** Requires molecular characterization.

**24. *Hylonomoiposakares* Reid, 1996**

**Synonyms.** None.

**Holotype.** Deposited in the Australian National Insect Collection, Canberra, Australia.

**Type locality.** Australia, Queensland, Lamington National Park (O’Reillys), 28°14'S, 153°08'E, ca 910 m ASL.

**Language of species description.** English.

**Remarks.** The species description includes specimens from different localities lying up to 55 km away from the type locality, hence the existence of a species complex within *Hylonomoiposakares* cannot be excluded. For the sake of caution, the name *Hylonomoiposakares* should only be applied to specimens from the type locality. Requires morphological revision and molecular characterization.

**25. *Hylonomoiposbrookensis* Reid, 1996**

**Synonyms.** None.

**Holotype.** Deposited in the Queensland Museum, Brisbane, Australia.

**Type locality.** Australia, Queensland, Upper Brookfield, 27°30'S, 152°55'E, ca 40 m ASL.

**Language of species description.** English.

**Remarks.** Requires molecular characterization.

##### XVI. *Konothele* Reid, 1996

**Type species.***Konothelekallimos* Reid, 1996, by monotypy (Reid 1996: 786).

**Remarks.** Requires molecular characterization.

**26. *Konothelekallimos* Reid, 1996**

**Synonyms.** None.

**Holotype.** Deposited in the Queensland Museum, Brisbane, Australia.

**Type locality.** Australia, Queensland, Mount Hemmant, 6 km southwest of Cape Tribulation, 16°07'S, 145°25'E, ca 880 m ASL.

**Language of species description.** English.

**Remarks.** The species description includes specimens from different localities lying up to 53 km away from the type locality, hence the existence of a species complex within *Konothelekallimos* cannot be excluded. For the sake of caution, the name *Konothelekallimos* should only be applied to specimens from the type locality. Requires morphological revision and molecular characterization.

##### XVII. *Kumbadjena* Reid, 2002

**Type species.***Kumbadjenaoccidentalis* (Fletcher, 1895), by original designation (Reid 2002: 131).

**27. *Kumbadjenaextrema* Sato, Buckman-Young, Harvey & Giribet, 2018**

**Synonyms.** None.

**Holotype.** Deposited in the Western Australian Museum, Perth, Australia.

**Type locality.** Australia, Western Australia, Limeburners Road, deep gully near Torndirrup NP, 35°05.45'S, 117°54.67'E, ca 40 m ASL (see Remarks).

**Language of species description.** English.

**Remarks.** Altitude data obtained from Google Earth based on the given geographic coordinates. *Kumbadjenaextrema* is described based on morphological and molecular data. The species description includes specimens from different localities lying up to 60 km away from the type locality, hence the existence of a species complex within *Kumbadjenaextrema* cannot be excluded. For the sake of caution, the name *Kumbadjenaextrema* should only be applied to specimens from the type locality. Requires revision.

**28. *Kumbadjenakaata* Reid, 2002**

**Synonyms.** None.

**Holotype.** Deposited in the Western Australian Museum, Perth, Australia.

**Type locality.** Australia, Western Australia, Porongurup National Park, Scenic Drive, 3.1 km west of intersection of Scenic Drive and Bolganup Road, 34°39'S, 117°51'E, ca 320 m ASL.

**Language of species description.** English.

**Remarks.** Molecular data available for this species (Sato et al. 2018).

**29. *Kumbadjenakarricola* Sato, Buckman-Young, Harvey & Giribet, 2018**

**Synonyms.** None.

**Holotype.** Deposited in the Western Australian Museum, Perth, Australia.

**Type locality.** Australia, Western Australia, Treen Brook State Forest, 100 m off Vasse Highway, 34°26.75'S, 115°59'E, ca 125 m ASL (see Remarks).

**Language of species description.** English.

**Remarks.** Altitude data obtained from Google Earth based on the given geographic coordinates. The species description includes morphological and molecular data.

**30. *Kumbadjenaoccidentalis* (Fletcher, 1895)**

**Synonyms.**Peripatusleuckartivar.occidentalis, as originally described (Fletcher 1895: 186); *Peripatoidesoccidentalis* (Dakin 1920: 367); *Occiperipatoidesoccidentalis* (Ruhberg 1985: 126); *Kumbadjenaoccidentalis* (Reid 2002: 132).

**Holotype.** Not designated (see Remarks). Neotype deposited in the Western Australian Museum, Perth, Australia.

**Type locality.** Australia, Western Australia, Bridgetown Jarrah Park, 20.3 km west of intersection of South Western Highway and Brockman Highway, 34°01'S, 116°00'E, ca 250 m ASL.

**Language of species description.** English.

**Remarks.** The holotype has not been designated explicitly in the original description. A neotype has been designated and deposited in the Western Australian Museum, Perth, Australia (see Reid 2002: 132, 136 for further information). Note that the synonym Peripatusleuckartivar.occidentalis may cause confusion with Peripatusleuckartivar.orientalis, the latter being a synonym of *Euperipatoidesleuckartii*. Molecular data, including transcriptome provided for *Kumbadjenaoccidentalis* (e.g., Sato et al. 2018; Baker et al. 2021) may not strictly correspond to the original species, as some of the sequenced specimens were collected up to 93 km west from the type locality.

**31. *Kumbadjenashannonensis* Reid, 2002**

**Synonyms.** None.

**Holotype.** Deposited in the Western Australian Museum, Perth, Australia.

**Type locality.** Australia, Western Australia, Shannon National Park, Giant Karri Grove, Deeside Coast Road, 5 km south of intersection of Middleton Road and Deeside Coast Road, 34°38'S, 116°20'E, ca 150 m ASL.

**Language of species description.** English.

**Remarks.** Molecular data available for this species (Sato et al. 2018).

**32. *Kumbadjenatoolbrunupensis* Sato, Buckman-Young, Harvey & Giribet, 2018**

**Synonyms.** None.

**Holotype.** Deposited in the Western Australian Museum, Perth, Australia.

**Type locality.** Australia, Western Australia, Stirling Range National Park, Toolbrunup Peak, 750 m northwest of carpark, 34°23.42'S, 118°03.3'E, ca 560 m ASL (see Remarks).

**Language of species description.** English.

**Remarks.** Altitude data obtained from Google Earth based on the given geographic coordinates. The species description includes morphological and molecular data.

##### XVIII. *Lathropatus* Reid, 2000a

**Type species.***Lathropatusnemorum* Reid, 2000a, by monotypy (Reid 2000a: 154).

**Remarks.** Requires molecular characterization.

**33. *Lathropatusnemorum* Reid, 2000a**

**Synonyms.** None.

**Holotype.** Deposited in the Melbourne Museum [originally described as Museum Victoria], Melbourne, Australia.

**Type locality.** Australia, Victoria, Cobboboonee Nation Park [previously State Forest], Southern end, approximately 11.4 km northwest of Portland, beside Elbow Road, off Nelson Portland Road, 38°17'S, 141°33'E, ca 60 m ASL.

**Language of species description.** English.

**Remarks.** The species description includes specimens from different localities lying up to 30 km away from the type locality, hence the existence of a species complex within *Lathropatusnemorum* cannot be excluded. For the sake of caution, the name *Lathropatusnemorum* should only be applied to specimens from the type locality. Requires morphological revision and molecular characterization.

##### XIX. *Leucopatus* Oliveira, Ruhberg, Rowell & Mayer, 2018

**Type species.***Leucopatusanophthalmus* (Ruhberg, Mesibov, Briscoe & Tait, 1991) by monotypy (Oliveira et al. 2018: 925).

**34. *Leucopatusanophthalmus* (Ruhberg, Mesibov, Briscoe & Tait, 1991)**

**Synonyms.***Tasmanipatusanophthalmus* (Ruhberg et al. 1991: 9).

**Holotype.** Deposited in the Queen Victoria Museum and Art Gallery, Launceston, Australia.

**Type locality.** Australia, Tasmania, Elephant Pass, 4l°38'S, 148°14'E, ca 380 m ASL (see Remarks).

**Language of species description.** English.

**Remarks.** Altitude data provided by Oliveira et al. (2018). Species redescription includes morphological, molecular, karyotype and slime protein profiling data (Oliveira et al. 2018). The species name has been misspelt as *anopthalmus* [sic] by Reid (1996: 881). Additional molecular data available for this species (e.g., Murienne et al. 2014; Giribet et al. 2018). According to Oliveira et al. (2018: 930) the existence of a species complex within *Leucopatusanophthalmus* cannot be excluded. For the sake of caution, the name *Leucopatusanophthalmus* should only be applied to specimens from the type locality. Geographic coordinates assigned to *Leucopatusanophthalmus* in Giribet at al. (2018) may contain a typo.

##### XX. *Leuropezos* Reid, 1996

**Type species.***Leuropezoseungellensis* Reid, 1996, by monotypy (Reid 1996: 792).

**Remarks.** Requires molecular characterization.

**35. *Leuropezoseungellensis* Reid, 1996**

**Synonyms.** None.

**Holotype.** Deposited in the Australian National Insect Collection, Canberra, Australia.

**Type locality.** Australia, Queensland, Eungella National Park, Crediton Creek, 21°11'S, 148°33'E, ca 750 m ASL.

**Language of species description.** English.

**Remarks.** Requires molecular characterization.

##### XXI. *Mantonipatus* Ruhberg, 1985

**Type species.***Mantonipatuspersiculus* Ruhberg, 1985, by monotypy (Ruhberg 1985: 121).

**Remarks.** Requires molecular characterization.

**36. *Mantonipatuspersiculus* Ruhberg, 1985**

**Synonyms.** None.

**Holotype.** Deposited in the Zoologisches Institut und Zoologisches Museum, University of Hamburg, Hamburg, Germany.

**Type locality.** Australia, South Australia, Mount Lofty Range, Carey Gully, Wotton’s Scrub, 34°58'S, 138°46'E, ca 480 m ASL.

**Language of species description.** German.

**Remarks.** Requires molecular characterization.

##### XXII. *Metaperipatus* Clark, 1913a

**Type species.***Metaperipatusblainvillei* (Gervais, 1837), by monotypy (Clark 1913a: 18).

**37. *Metaperipatusinae* Mayer, 2007**

**Synonyms.** None.

**Holotype.** Deposited in the Museo Zoológico de la Universidad de Concepción, Concepción, Chile.

**Type locality.** Chile, VIII region del Bío-Bío, forest near Contulmo, 38°01'S, 73°11'W, ca 390 m ASL.

**Language of species description.** English.

**Remarks.** The complete mitochondrial genome is available for this species (Braband et al. 2010a). Additional molecular data including transcriptome have recently become available for *Metaperipatusinae* (e.g., Murienne et al. 2014; Giribet et al. 2018; Baker et al. 2021).

**Nomen dubium**

**nd1. *Metaperipatusblainvillei* (Gervais, 1837)**

**Synonyms.***Veniliablainvillei* (Gervais 1837: 38 as footnote); *Peripatusblainvillei* (Blanchard 1847: 140); *Peripatuschiliensis* (Sedgwick 1888: 480); *Peripatoidesblainvillei* (Bouvier 1901b: 59); *Peripatopsisblainvillei* (Bouvier 1901b: 61); *Metaperipatusblainvillei* (Clark 1913a: 18) (see Remarks).

**Holotype.** Not designated (see Remarks).

**Type locality.** Chile (see Remarks).

**Language of species description.** French.

**Remarks.** An incorrect author [Blanchard] is commonly assigned to *Metaperipatusblainvillei* (e.g., Clark 1913a: 18). This species was named provisionally *Veniliablainvillei* in a letter of M. Gay to M. de Blainville (see Gervais 1837: 38). The species name has been misspelt *T.blainvillii* [sic] Blanch. by Schmarda (1878: 77) and *Metaperipatusblainsvillei* [sic] by Daniels et al. (2016: 515). The holotype has not been designated explicitly in the original description. Oliveira et al. (2012a: 37) regarded *Metaperipatusblainvillei* as nomen dubium, since the precise locality of the first record is unknown and the type material has been lost (see Bouvier 1901b: 59). Specimens collected from different localities across a large area have been assigned to *Metaperipatusblainvillei* (e.g., Bouvier 1901b: 59; Johow 1911: 81–82; Ruhberg 1985: 108; Mayer 2007: 22). The wide distribution, together with an unusual variation in the number of leg pairs in specimens from different localities (see Mayer 2007), suggest that the existence of a species complex within *Metaperipatusblainvillei* cannot be excluded and revision would be required. Nevertheless, the lack of precise collecting data and type material will make a revision of this species difficult. Molecular data have become available for specimens collected in the environs of Lago Tinquilco, IX Region de la Araucania, 39°09'S, 71°42'W, ca 815 m (Murienne et al. 2014; Oliveira et al. 2018; Giribet et al. 2018).

##### XXIII. *Minyplanetes* Reid, 1996

**Type species.***Minyplaneteskroombensis* Reid, 1996, by monotypy (Reid 1996: 798).

**Remarks.** Requires molecular characterization.

**38. *Minyplaneteskroombensis* Reid, 1996**

**Synonyms.** None.

**Holotype.** Deposited in the Queensland Museum, Brisbane, Australia.

**Type locality.** Australia, Queensland, Kroombit Tops, 24°25'S, 151°03'E, ca 940 m ASL.

**Language of species description.** English.

**Remarks.** The species description includes specimens from different localities lying up to 61 km away from the type locality, hence the existence of a species complex within *Minyplaneteskroombensis* cannot be excluded. For the sake of caution, the name *Minyplaneteskroombensis* should only be applied to specimens from the type locality. Requires morphological revision and molecular characterization.

##### XXIV. *Nodocapitus* Reid, 1996

**Type species.***Nodocapitusbarryi* Reid, 1996, by original designation (Reid 1996: 802).

**Remarks.** Requires molecular characterization.

**39. *Nodocapitusbarryi* Reid, 1996**

**Synonyms.** None.

**Holotype.** Deposited in the Australian Museum, Sydney, Australia.

**Type locality.** Australia, New South Wales, Richmond Range National Park [previously State Forest], 28°40'S, 152°45'E, ca 400 m ASL.

**Language of species description.** English.

**Remarks.** The species description includes specimens from different localities lying up to 87 km away from the type locality, hence the existence of a species complex within *Nodocapitusbarryi* cannot be excluded. For the sake of caution, the name *Nodocapitusbarryi* should only be applied to specimens from the type locality. Requires morphological revision and molecular characterization.

**40. *Nodocapitusformosus* Reid, 1996**

**Synonyms.** None.

**Holotype.** Deposited in the Australian National Insect Collection, Canberra, Australia.

**Type locality.** Australia, Queensland, Mount Elliot, 19°29'S, 146°59'E, ca 1,050 m ASL.

**Language of species description.** English.

**Remarks.** Requires molecular characterization.

**41. *Nodocapitusinornatus* Reid, 1996**

**Synonyms.** None.

**Holotype.** Deposited in the Australian Museum, Sydney, Australia.

**Type locality.** Australia, New South Wales, Gibraltar Range National Park, 29°28'S, 152°21'E, ca 900 m ASL.

**Language of species description.** English.

**Remarks.** Requires molecular characterization.

##### XXV. *Occiperipatoides* Ruhberg, 1985

**Type species.***Occiperipatoidesgilesii* (Spencer, 1909), by original designation (Ruhberg 1985: 124) (see Remarks).

**Remarks.** The genus originally contained two nominal species, but *Occiperipatoidesoccidentalis* (Fletcher, 1895) is now treated under the name *Kumbadjenaoccidentalis* (Fletcher, 1895) (see Ruhberg 1985; Reid 1996).

**42. *Occiperipatoidesgilesii* (Spencer, 1909)**

**Synonyms.***Peripatoideswoodwardi* Bouvier, 1909 (junior synonym, nomen nudum; Bouvier 1909: 315); *Peripatoidesgilesii* (Spencer 1909: 240); *Occiperipatoidesgilesi* (Ruhberg 1985: 124) (see Remarks).

**Holotype.** Not designated (see Remarks).

**Type locality.** Australia, Western Australia, Armadale, 32°09'S, 116°00'E, ca 45 m ASL (see Remarks).

**Language of species description.** English.

**Remarks.** The name *Peripatoideswoodwardi* is regarded as a junior synonym, as it has been published nine months after the description of *Peripatusgilesii* (Oliveira et al. 2012a: 38). Hence, Oliveira et al. (2012a: 38) regarded *Peripatoideswoodwardi* Bouvier, 1909 as nomen nudum to avoid nomenclatural inconsistencies. The holotype of *Occiperipatoidesgilesii* has not been designated explicitly in the original description. Ruhberg (1985: 125) and Reid (1996: 815) mention syntypes deposited in three institutions: The Museum National d’Histoire Naturelle de Paris (France; not listed by Le Bras et al. 2015), the Natural History Museum of London (UK), and the Zoologisches Staatsinstitut und Zoologisches Museum Hamburg (Germany). Weidner (1959: 93) refers to a holotype and a ‘paratypoid’ in the Zoologisches Staatsinstitut und Zoologisches Museum Hamburg, Germany, under the name *Peripatoideswoodwardi*. Röhlig et al. (2010: 227) also refer to a paratype with the same name placed in the Museum für Naturkunde Berlin, Germany. According to the ICZN (Art. 72.9), the ‘types’ associated with the name *Peripatoideswoodwardi* (Weidner 1959: 93; Röhlig et al. 2010: 227) should be regarded as syntypes of *Occiperipatoidesgilesii*. The species name is commonly misspelt as *gilesi* (e.g., Ruhberg 1985: 124). Altitude data obtained from Google Earth based on the geographic coordinates provided by Reid (1996: 815). The species redescription (Reid 1996: 814) includes specimens from different localities lying up to 76 km away from the type locality, hence the existence of a species complex within *Occiperipatoidesgilesii* cannot be excluded. For the sake of caution, the name *Occiperipatoidesgilesii* should only be applied to specimens from the type locality. Molecular data including transcriptome available for this species (e.g., Murienne et al. 2014; Giribet et al. 2018; Baker et al. 2021). Requires revision.

##### XXVI. *Ooperipatellus* Ruhberg, 1985

**Type species.***Ooperipatellusinsignis* (Dendy, 1890), by original designation (Ruhberg 1985: 127).

**43. *Ooperipatelluscryptus* Jackson & Taylor, 1995**

**Synonyms.** None.

**Holotype.** Not designated (see Remarks).

**Type locality.** Australia, Tasmania, Christmas Hills, Arthur River, Rapid River area (see Remarks).

**Language of species description.** English.

**Remarks.** Although the initial pages of Jackson and Taylor’s book, which contains the species description, indicate the year 1994, the work was not published until early 1995 (R. Mesibov in litt.). This species has previously been regarded as nomen dubium by Oliveira et al. (2012a: 40) based on Art. 8.5.2 of the ICZN. This article, however, refers to the nomenclatural availability of names found in works issued and distributed electronically. Since the publication by Jackson and Taylor (1995) has been issued and distributed in printed format, and since nomenclatural availability of a name is not related to its doubtful application (nomen dubium), the previous designation of *Ooperipatelluscryptus* as nomen dubium by Oliveira et al. (2012a: 40) is revoked herein. The holotype has not been designated explicitly in the original description (see also Reid 1996: 909). This species is assigned to an imprecise area of more than 2,000 km^2^ in north-western Tasmania, hence the existence of a species complex within *Ooperipatelluscryptus* cannot be excluded. Nevertheless, Jackson and Taylor (1995: 167) refer to a main population occurring in the Arthur River/Rapid River area, which is herein regarded as the type locality. Molecular data recently assigned to *Ooperipatelluscryptus* (see Giribet et al. 2018) may not strictly correspond to the original species, as the sequenced specimens were collected more than 60 km away from the main population area. Specimens studied by Murienne et al. (2014) followed by Oliveira and Mayer (2017) have not been assigned with locality data. Requires revision and type designation.

**44. *Ooperipatellusdecoratus* (Baehr, 1977)**

**Synonyms.***Ooperipatusdecoratus*, as originally described (Baehr 1977: 14); *Ooperipatellusinsignis* (Ruhberg 1985: 128); *Ooperipatellusdecoratus* (Reid 1996: 909).

**Holotype.** Deposited in the Australian National Insect Collection, Canberra, Australia.

**Type locality.** Australia, Tasmania, Mawbanna, Dip River Falls, 8 km south of Mawbama, northwest Tasmania, ca 250 m ASL.

**Language of species description.** German.

**Remarks.** The species had previously been synonymized with *Ooperipatellusinsignis* (Ruhberg 1985: 128). Oliveira et al. (2012a: 39) regarded *Ooperipatellusdecoratus* and *Ooperipatellusinsignis* as separate species since their type localities lie 411 km apart from each other and are separated by the Bass Strait. Molecular data recently assigned to *Ooperipatellusdecoratus* (see Giribet et al. 2018) may not strictly correspond to the original species, as the sequenced specimens were collected 68 km away from the type locality. Specimens studied by Murienne et al. (2014) and Oliveira and Mayer (2017) have not been assigned with locality data. Requires revision.

**45. *Ooperipatellusduwilensis* Reid, 1996**

**Synonyms.** None.

**Holotype.** Deposited in the Melbourne Museum [originally described as Museum Victoria], Melbourne, Australia.

**Type locality.** Australia, Victoria, Grampians National Park, Mount William, 37°18'S, 142°36'E, ca 1,260 m ASL.

**Language of species description.** English.

**Remarks.** Requires molecular characterization.

**46. *Ooperipatellusinsignis* (Dendy, 1890)**

**Synonyms.***Peripatusinsignis*, as originally described (Dendy 1890: 174); Peripatusleuckartiivar.typica (Fletcher 1895: 185; see Bouvier 1907b: 268); *Ooperipatusinsignis* (Dendy 1900a: 510); *Ooperipatellusinsignis* (Ruhberg 1985: 127).

**Holotype.** Not designated (see Remarks).

**Type locality.** Australia, Victoria, Mount Macedon, 37°23'S, 144°35'E, ca 1,000 m ASL.

**Language of species description.** English.

**Remarks.** The holotype has not been designated explicitly in the original description. Bouvier (1907a: 521) refers to a cotype [obsolete for syntype] deposited in the Museum National d’Histoire Naturelle de Paris, France (see also Le Bras et al. 2015). The species has been revised by Reid (1996: 822–824). *Ooperipatellusinsignis* seems to occur all over Mount Macedon, while the sympatric species *Ooperipatusoviparus* is restricted to the lower areas. Molecular data available for *Ooperipatellusinsignis* support it as a separate species from Tasmanian and New Zealand *Ooperipatellus* (Oliveira and Mayer 2017; Giribet et al. 2018), as already suggested by Clark (1913a: 19, as footnote). Slime protein profiling data provided previously by Baer et al. (2014).

**47. *Ooperipatellusnanus* Ruhberg, 1985**

**Synonyms.** None.

**Holotype.** Not designated (see Remarks).

**Type locality.** New Zealand, South Island, Takitimu Range, Cheviot Face, ca 1,160 m ASL.

**Language of species description.** German.

**Remarks.** Since the original species description was based on four juveniles (Ruhberg 1985: 131), the author decided to not designate a holotype. According to Ruhberg (1985: 131), the syntypes are deposited in Entomology Division of the Department of Scientific and Industrial Research, Auckland, New Zealand. Molecular data available for this species (e.g., Murienne et al. 2014; Oliveira and Mayer 2017; Giribet et al. 2018). Requires morphological revision and molecular characterization.

**48. *Ooperipatellusnickmayeri* Oliveira & Mayer, 2017**

**Synonyms.** None.

**Holotype.** Deposited in the Queen Victoria Museum and Art Gallery, Launceston, Tasmania, Australia.

**Type locality.** Australia, Tasmania, small fragment of forest on the Lyell Highway (Route A10), approximately 9 km driving from Tarraleah (to the NW) and 6.8 km from Wayatinah (to the SE), 42°20.61'S, 146°28.23'E, ca 545 m ASL.

**Language of species description.** English.

**Remarks.** Species description includes morphological, molecular and karyotype data. Slime protein profiling data provided previously by Baer et al. (2014) under the name *Ooperipatellus* sp.

**49. *Ooperipatellusparvus* Reid, 1996**

**Synonyms.** None.

**Holotype.** Deposited in the South Australian Museum, Adelaide, Australia.

**Type locality.** Australia, South Australia, Mount Lofty Range, Mylor, beside Onkaparinga River, 35°03'S, 138°46'E, ca 320 m ASL.

**Language of species description.** English.

**Remarks.** Requires molecular characterization.

**50. *Ooperipatellusspenceri* (Cockerell, 1913b)**

**Synonyms.***Ooperipatusinsignis* (Bouvier 1907b: 267); *Ooperipatusspenceri*, as originally described (Cockerell 1913b in Clark 1913a: 19); *Ooperipatellusinsignis* (Ruhberg 1985: 128); *Ooperipatellusspenceri* (Oliveira et al. 2012a: 40) (see Remarks).

**Holotype.** Not designated (see Remarks).

**Type locality.** Australia, Tasmania, Wellington Park, Mount Wellington.

**Language of species description.** English.

**Remarks.***Ooperipatellusspenceri* was initially assigned to *Ooperipatellusinsignis* (Bouvier 1907b: 267), but since the latter species does not occur in Tasmania, Cockerell (1913b) suggested the new name *spenceri* in a footnote of Clark’s work (1913a: 19). The holotype has not been designated explicitly in the original description. *Ooperipatellus* [then *Ooperipatus*] *spenceri* was subsequently synonymized with *Ooperipatellusinsignis* by Baehr (1977: 13) (see also Ruhberg 1985: 128). However, Oliveira et al. (2012a: 40), following Cockerell (1913b: 19 in Clark 1913a), regarded *Ooperipatellusspenceri* and *Ooperipatellusinsignis* as separate species since their type localities lie 652 km apart from each other and are separated by the Bass Strait. Requires morphological revision, molecular characterization, and type designation.

**51. *Ooperipatellusviridimaculatus* (Dendy, 1900b)**

**Synonyms.***Peripatusviridimaculatus*, as originally described (Dendy 1900b: 436); *Ooperipatusviridimaculatus* (Dendy 1900a: 510); *Ooperipatellusinsignis* (Ruhberg 1985: 128); *Ooperipatellusviridimaculatus* (Oliveira et al. 2012a: 40) (see Remarks).

**Holotype.** Not designated (see Remarks).

**Type locality.** New Zealand, South Island, ‘in the dense beech forest at the head of Lake Te Anau’.

**Language of species description.** English.

**Remarks.** The name *Ooperipatellusviridimaculatus* was first introduced in the abstract of the Annual Meeting of the Philosophical Institute of Canterbury (Dendy 1900b). The holotype has not been designated explicitly in the original description. *Ooperipatellusviridimaculatus* had previously been synonymized with *Ooperipatellusinsignis* (Ruhberg 1985: 128). However, molecular studies indicate that New Zealand *Ooperipatellus* are unlikely to be conspecific with those from Tasmania and mainland Australia (e.g., *Ooperipatellusinsignis*) (Tait and Briscoe 1995; Allwood et al. 2010; Murienne et al. 2014; Oliveira and Mayer 2017; Giribet et al. 2018). Hence, Oliveira et al. (2012a: 40) regarded *Ooperipatellusviridimaculatus* and *Ooperipatellusinsignis* as separate species, as their type localities lie 2,113 km apart from each other and are separated by the Tasman Sea. Early molecular data available for *Ooperipatellusviridimaculatus* were obtained from specimens collected up to 420 km away from the type locality (Allwood et al. 2010). More recent transcriptome data assigned to this species were obtained from specimens collected 180 km away from the type locality (Baker et al. 2021). Thus, the existence of a species complex within *Ooperipatellusviridimaculatus* cannot be excluded. For the sake of caution, the name *Ooperipatellusviridimaculatus* should only be applied to specimens from the type locality. Requires morphological revision, molecular characterization, and type designation.

##### XXVII. *Ooperipatus* Dendy, 1900a

**Type species.***Ooperipatusoviparus* (Dendy, 1895), by subsequent designation (Dendy 1902: 367).

**Remarks.** The genus *Symperipatus* suggested by Cockerell (1913b; as a footnote in Clark 1913a: 19) is regarded as objective synonym of *Ooperipatus*, as it was raised based on the same name-bearing type, namely *Ooperipatusoviparus* (ICZN Art. 61.3.3; see Oliveira et al. 2012a: 42). Requires more comprehensive molecular characterization.

**52. *Ooperipatusbirrgus* Reid, 2000a**

**Synonyms.** None.

**Holotype.** Deposited in the Melbourne Museum [originally described as Museum Victoria], Melbourne, Australia.

**Type locality.** Australia, New South Wales, South East Forest National Park, Coolangubra Section, 5 km north of intersection of Coolangubra Forest Way and Northern Access Road, 37°01'S, 149°23'E, ca 800 m ASL.

**Language of species description.** English.

**Remarks.** Molecular data available for this species (Murienne et al. 2014).

**53. *Ooperipatuscaesius* Reid, 2000a**

**Synonyms.** None.

**Holotype.** Deposited in the Melbourne Museum [originally described as Museum Victoria], Melbourne, Australia.

**Type locality.** Australia, Victoria, Mount Buffalo National Park, Track to Eurobin Falls, 36°43'S, 146°50'E, ca 500 m ASL.

**Language of species description.** English.

**Remarks.** Molecular data available for this species (e.g., Murienne et al. 2014; Giribet et al. 2018).

**54. *Ooperipatuscentunculus* Reid, 1996**

**Synonyms.** None.

**Holotype.** Deposited in the Melbourne Museum [originally described as Museum Victoria], Melbourne, Australia.

**Type locality.** Australia, Victoria, Mount Donna Buang, 37°42'S, 145°41'E, ca 1,250 m ASL.

**Language of species description.** English.

**Remarks.** Requires molecular characterization.

**55. *Ooperipatuscostatus* Reid, 1996**

**Synonyms.** None.

**Holotype.** Deposited in the Australian Museum, Sydney, Australia.

**Type locality.** Australia, Australian Capital Territory, Namadgi National Park, Stockyard Gap, 35°33'S, 148°46'E, ca 1,560 m ASL.

**Language of species description.** English.

**Remarks.** The species description includes specimens from different localities lying up to 109 km away from the type locality, hence the existence of a species complex within *Ooperipatuscostatus* cannot be excluded. For the sake of caution, the name *Ooperipatuscostatus* should only be applied to specimens from the type locality. Requires morphological revision and molecular characterization.

**56. *Ooperipatushispidus* Reid, 1996**

**Synonyms.** None.

**Holotype.** Deposited in the Australian Museum, Sydney, Australia.

**Type locality.** Australia, New South Wales, Tallaganda State Forest, Forbes Creek Road, 35°28'S, 149°32'E, ca 1,000 m ASL.

**Language of species description.** English.

**Remarks.** The species description includes specimens from different localities lying up to 60 km away from the type locality, hence the existence of a species complex within *Ooperipatushispidus* cannot be excluded. For the sake of caution, the name *Ooperipatushispidus* should only be applied to specimens from the type locality. Requires morphological revision and molecular characterization.

**57. *Ooperipatuslepidus* Reid, 2000a**

**Synonyms.** None.

**Holotype.** Deposited in the Melbourne Museum [originally described as Museum Victoria], Melbourne, Australia.

**Type locality.** Australia, Victoria, Granite Flat, 9 km south of Mitta Mitta, beside Omeo Highway, 350 m north of intersection of Omeo Highway and Walsh’s Road, 36°35'S, 147°27'E, ca 340 m ASL.

**Language of species description.** English.

**Remarks.** Requires molecular characterization.

**58. *Ooperipatusnebulosus* Reid, 2000a**

**Synonyms.** None.

**Holotype.** Deposited in the Melbourne Museum [originally described as Museum Victoria], Melbourne, Australia.

**Type locality.** Australia, Victoria, Mirimbah, Carters Mill Campground, 950 m along Carters Road from Mount Buller Road, 37°06'S, 146°22'E, ca 640 m ASL.

**Language of species description.** English.

**Remarks.** Requires molecular characterization.

**59. *Ooperipatusoviparus* (Dendy, 1895)**

**Synonyms.***Peripatusoviparus*, as originally described (Dendy 1895: 195); *Ooperipatusoviparus* (Dendy 1900a: 510); *Symperipatusoviparus* (Cockerell 1913b; as a footnote in Clark 1913a: 19) (see Remarks).

**Holotype.** Not designated (see Remarks). Lectotype deposited in the Muséum National d’Histoire Naturelle, Paris, France.

**Type locality.** Australia, Victoria, Mount Macedon, 37°23'S, 144°35'E, ca 900 m ASL (see Remarks).

**Language of species description.** English.

**Remarks.***Symperipatus* (Cockerell 1913b; as a footnote in Clark 1913a: 19) was raised based on the same name-bearing type as *Ooperipatus*, i.e., *Ooperipatusoviparus*, thus being an objective synonym of the latter (Oliveira et al. 2012a: 42; ICZN Art. 61.3.3). The holotype has not been designated explicitly in the original description. The species has been revised and a lectotype has been designated by Reid (1996). The lectotype deposited in the Muséum National d’Histoire Naturelle, Paris, France (Reid 1996: 830) correspond to the ‘type’ from Mount Macedon listed by Le Bras et al. (2015). The additional specimen from Mount Baw Baw listed by Le Bras et al. (2015) as ‘type’ requires verification. *Ooperipatusoviparus* mainly inhabits lower areas of Mount Macedon, while the sympatric species *Ooperipatellusinsignis* occur in all altitudes. Slime protein profiling data provided by Baer et al. (2014). Requires morphological revision and molecular characterization.

**60. *Ooperipatusporcatus* Reid, 2000a**

**Synonyms.** None.

**Holotype.** Deposited in the Melbourne Museum [originally described as Museum Victoria], Melbourne, Australia.

**Type locality.** Australia, Victoria, Mount Useful Scenic Reserve, 14.5 km north of intersection of Binns Road and McEvoys Track, 37°43'S, 146°31'E, ca 750 m ASL.

**Language of species description.** English.

**Remarks.** Molecular data available for this species (e.g., Murienne et al. 2014; Giribet et al. 2018).

**61. *Ooperipatuspulchellus* Reid, 1996**

**Synonyms.** None.

**Holotype.** Deposited in the Melbourne Museum [originally described as Museum Victoria], Melbourne, Australia.

**Type locality.** Australia, Victoria, Baw Baw National Park, Mount Baw Baw, 37°50'S, 146°17'E, ca 1,570 m ASL.

**Language of species description.** English.

**Remarks.** Requires molecular characterization.

**62. *Ooperipatussilvanus* Reid, 2000a**

**Synonyms.** None.

**Holotype.** Deposited in the Melbourne Museum [originally described as Museum Victoria], Melbourne, Australia.

**Type locality.** Australia, Victoria, Otway range, 0.1 km south of intersection of Young Creek Track and Philips Road, 38°40'S, 143°30'E, ca 260 m ASL.

**Language of species description.** English.

**Remarks.** Requires molecular characterization.

##### XXVIII. *Opisthopatus* Purcell, 1899

**Type species.***Opisthopatuscinctipes* Purcell, 1899, by original designation (Purcell 1899: 349).

**Remarks.** The genus *Opisthopatus* was rejected by Sedgwick (1908: 405), although the name has continuously been used in the literature (see Ruhberg 1985). Recent molecular data support *Opisthopatus* as a valid genus (e.g., Daniels et al. 2016; Murienne et al. 2014; Giribet et al. 2018).

**63. *Opisthopatusamaxhosa* Daniels, Dambire, Klaus & Sharma, 2016**

**Synonyms.** None.

**Holotype.** Deposited in the Entomological Collection of the South African Museum (Iziko Museums of Cape Town), Cape Town, South Africa.

**Type locality.** South Africa, Eastern Cape, Jenca Valley, Langeni area, 31°21.96'S, 28°33.43'E, ca 1,410 m ASL (see Remarks).

**Language of species description.** English.

**Remarks.** Altitude data obtained from Google Earth based on the given geographic coordinates. Geographic coordinates presented in ‘table 1 [List of sample localities]’ by Daniels et al. (2016: 510) may contain a typo (minus sign associated with longitude rather than latitude); correct and more precise coordinates provided along the species description. Species described based on morphological and molecular data. *Opisthopatusamaxhosa* turned out to be a species complex, which has been investigated by Barnes and Daniels (2022).

**64. *Opisthopatusbaziya* Barnes & Daniels, 2022**

**Synonyms.** None.

**Holotype.** Deposited in the Entomological Collection of the South African Museum (Iziko Museums of Cape Town), Cape Town, South Africa.

**Type locality.** South Africa, Eastern Cape, Baziya Forest, Baziya A, 31°34.03'S, 28°24.73'E, ca 1,105 m ASL (see Remarks).

**Language of species description.** English.

**Remarks.** Geographic coordinates provided for type specimens in the original description (Barnes and Daniels 2022: 10) do not seem to correspond to the given locality name and altitude: longitude data may contain a typo in the degree position (29° instead of 28°). *Opisthopatusbaziya* is described based on morphological and molecular data.

**65. *Opisthopatuscamdebooi* Barnes & Daniels, 2022**

**Synonyms.** None.

**Holotype.** Deposited in the Entomological Collection of the South African Museum (Iziko Museums of Cape Town), Cape Town, South Africa.

**Type locality.** South Africa, Eastern Cape, Camdeboo National Park, Valley of Desolation, 32°15.95'S, 24°29.51'E, ca 1,350 m ASL.

**Language of species description.** English.

**Remarks.***Opisthopatuscamdebooi* is described based on morphological and molecular data.

**66. *Opisthopatuscinctipes* Purcell, 1899**

**Synonyms.** None.

**Holotype.** Not designated (see Remarks).

**Type locality.** South Africa, Eastern Cape, Uitenhage, Dunbrody, near Blue Cliff Station (see Remarks).

**Language of species description.** English.

**Remarks.** The holotype has not been designated explicitly in the original description. Specimens of *Opisthopatuscinctipes* studied by Purcell (1899) had originally been deposited in the South African Museum of Natural History. Whether or not these syntypes are still preserved at this institution could not be verified. Bouvier (1907a: 520) refers to a cotype [obsolete for syntype] deposited in the Museum National d’Histoire Naturelle de Paris, France (see also Le Bras et al. 2015). The name *Opisthopatuscinctipes* had previously been assigned to specimens collected in several areas across Eastern Cape, KwaZulu-Natal and Mpumalanga provinces. More recently, the *Opisthopatuscinctipes* species complex has been revised and the occurrence range of this species has been restricted to Eastern Cape Afrotemperate and Indian Ocean Coastal Belt forests (Daniels et al. 2016: 530). This revision, however, did not include specimens from the type locality (topotypes). Since Daniels et al. (2016) recorded *Opisthopatuscinctipes* for multiple sparse localities, the existence of a species complex within *Opisthopatuscinctipes* still cannot be excluded. Molecular data, including the mitochondrial genome provided for *Opisthopatuscinctipes* (Braband et al. 2010b; Murienne et al. 2014) may not strictly correspond to the original species, as the sequenced specimens were collected 508–612 km away from the type locality. For the sake of caution, the name *Opisthopatuscinctipes* should only be applied to specimens from the type locality. Requires further revision.

**67. *Opisthopatusdrakensbergi* Daniels, Dambire, Klaus & Sharma, 2016**

**Synonyms.** None.

**Holotype.** Deposited in the Entomological Collection of the South African Museum (Iziko Museums of Cape Town), Cape Town, South Africa.

**Type locality.** South Africa, KwaZulu-Natal, Royal Natal National Park, 28°41.37'S, 28°56.25'E, ca 1,480 m ASL (see Remarks).

**Language of species description.** English.

**Remarks.** Altitude data obtained from Google Earth based on the given geographic coordinates. Geographic coordinates presented in ‘table 1 [List of sample localities]’ by Daniels et al. (2016: 510) may contain a typo (minus sign associated with longitude rather than latitude); correct and more precise coordinates provided along the species description. *Opisthopatusdrakensbergi* is described based on morphological and molecular data. The species description includes specimens from different localities lying up to 378 km away from the type locality, hence the existence of a species complex within *Opisthopatusdrakensbergi* cannot be excluded. For the sake of caution, the name *Opisthopatusdrakensbergi* should only be applied to specimens from the type locality. Requires revision.

**68. *Opisthopatusherbertorum* Ruhberg & Hamer, 2005**

**Synonyms.** None (see Remarks).

**Holotype.** Deposited in the Natal Museum, Pietermaritzburg, South Africa (see Remarks).

**Type locality.** South Africa, KwaZulu-Natal, Mount Currie Nature Reserve, near Kokstad, alongside road between main entrance and pass, in forest patch near ravine, 30°17.22'S, 29°13.67'E, ca 1,460 m ASL (see Remarks).

**Language of species description.** English.

**Remarks.** This species has recently been regarded as junior synonym of *Opisthopatusroseus* by Daniels et al. (2016: 531). However, these two species appear separated by long branches in the molecular phylogenetic tree (Daniels et al. 2016: 518), besides occurring relatively far away from each other: the type locality of *Opisthopatusherbertorum* lies 52 km northwest from the one of *Opisthopatusroseus*. Thus, conspecificity is, in this case, still inconclusive and for the sake of caution, I opted to regard *Opisthopatusherbertorum* as a valid name until further investigations are conducted. The allotype designated for this species (Ruhberg and Hamer 2005: 29) has no name-bearing function (see Recommendation 72A of the ICZN). Altitude data obtained from Google Earth based on the given geographic coordinates. Molecular data have recently been provided for this species (Daniels et al. 2016).

**69. *Opisthopatushighveldi* Daniels, Dambire, Klaus & Sharma, 2016**

**Synonyms.** None.

**Holotype.** Improperly designated (see Remarks).

**Type locality.** South Africa, Mpumalanga, Highveld, Graskop, 24°52.59'S, 30°53.29'E, ca 1,655 m ASL (see Remarks).

**Language of species description.** English.

**Remarks.** Two specimens have been designated as holotype in the original publication (Daniels et al. 2016: 531). Since the ICZN establishes that the holotype consists of “one, and only one, specimen” (Art. 73.1), the fixation of the name-bearing type, in this case, must be regarded as invalid. According to the availability criteria, however, new specific names published after 1999 must be accompanied in the original publication by the explicit fixation of a holotype, or syntypes, for the nominal taxon (ICZN Art. 16.4.1). To avoid nomenclatural instability by declaring *Opisthopatushighveldi* a nomen nudum and, following advice from members of the International Committee of Zoological Nomenclature, the two specimens deposited in the Entomological Collection of the South African Museum (Iziko Museums of Cape Town), Cape Town, South Africa, under the accession number SAM-ENW-C007163 should now be regarded as syntypes. This would be in line with the criteria established by the ICZN (Art. 73.2.1.1) for syntype designation, which says “(w)hen a nominal taxon is established after 1999, only those specimens expressly indicated by the author as those upon which the new taxon is based (see Article 72.3) are syntypes”. Accordingly, the seven specimens previously regarded as paratypes (SAM-ENW-C007162) must be excluded from the type series, as paratypes, per se, do not represent name-bearing types (ICZN 73.1); these specimens should now be regarded as additional material. Altitude data obtained from Google Earth based on the given geographic coordinates. Geographic coordinates presented in ‘table 1 [List of sample localities]’ by Daniels et al. (2016: 510) may contain a typo (minus sign associated with longitude rather than latitude); correct and more precise coordinates provided along the species description. Species described based on morphological and molecular data. Transcriptome data recently assigned to *Opisthopatushighveldi* (Baker et al. 2021) may not strictly correspond to the original species, as the sequenced specimen was collected more than 420 km away from the type locality. The species description includes specimens from different localities lying up to 427 km away from the type locality, hence the existence of a species complex within *Opisthopatushighveldi* cannot be excluded. For the sake of caution, the name *Opisthopatushighveldi* should only be applied to specimens from the type locality. Requires revision.

**70. *Opisthopatuskwazululandi* Daniels, Dambire, Klaus & Sharma, 2016**

**Synonyms.** None.

**Holotype.** Improperly designated (see Remarks).

**Type locality.** South Africa, KwaZulu-Natal, Vernon Crookes Nature Reserve, 32°36.52'S, 27°14.42'E, ca 990 m ASL (see Remarks).

**Language of species description.** English.

**Remarks.** Two specimens have been designated as holotype in the original publication (Daniels et al. 2016: 534). Since the ICZN establishes that the holotype consists of “one, and only one, specimen” (Art. 73.1), the fixation of the name-bearing type, in this case, must be regarded as invalid. According to the availability criteria, however, new specific names published after 1999 must be accompanied in the original publication by the explicit fixation of a holotype, or syntypes, for the nominal taxon (ICZN Art. 16.4.1). To avoid nomenclatural instability by declaring *Opisthopatuskwazululandi* a nomen nudum and, following advice from members of the International Committee of Zoological Nomenclature, the two specimens deposited in the Entomological Collection of the South African Museum (Iziko Museums of Cape Town), Cape Town, South Africa, under the accession number SAM-ENW-C007178 should now be regarded as syntypes. This would be in line with the criteria established by the ICZN (Art. 73.2.1.1) for syntype designation, which says “(w)hen a nominal taxon is established after 1999, only those specimens expressly indicated by the author as those upon which the new taxon is based (see Article 72.3) are syntypes”. Accordingly, the seven specimens previously regarded as paratypes (SAM-ENW-C007190) must be excluded from the type series, as paratypes, per se, do not represent name-bearing types (ICZN 73.1); these specimens should now be regarded as additional material. Altitude data obtained from Google Earth based on the given geographic coordinates. Species described based on morphological and molecular data. Geographic coordinates presented in ‘table 1 [List of sample localities]’ by Daniels et al. (2016: 510) may contain a typo (minus sign associated with longitude rather than latitude); correct and more precise coordinates provided along the species description. Transcriptome data recently assigned to *Opisthopatuskwazululandi* (Mapalo et al. 2020; Baker et al. 2021) may not strictly correspond to the original species, as the sequenced specimen was collected more than 940 km away from the type locality. The species description includes specimens from different localities lying up to 680 km away from the type locality, hence the existence of a species complex within *Opisthopatuskwazululandi* cannot be excluded. For the sake of caution, the name *Opisthopatuskwazululandi* should only be applied to specimens from the type locality. Requires revision.

**71. *Opisthopatusroseus* Lawrence, 1947**

**Synonyms.** None.

**Holotype.** Not designated (see Remarks).

**Type locality.** South Africa, KwaZulu-Natal, East Griqualand, Ingeli [Ngele] Forest, near Kokstad.

**Language of species description.** English.

**Remarks.** Amending Oliveira et al. (2012a: 44), the holotype of *Opisthopatusroseus* has not been designated explicitly in the original description. The syntypes are deposited in the Natal Museum, Pietermaritzburg, South Africa (Lawrence 1947: 165; Ruhberg 1985: 86). The specimens mistakenly regarded as holotypes [sic] by Daniels et al. (2016: 531) correspond to syntypes. Species recently redescribed based on morphological and molecular data (Daniels et al. 2016). Additional molecular data available for this species (e.g., Murienne et al. 2014; Giribet et al. 2018).

**72. *Opisthopatusswatii* Daniels, Dambire, Klaus & Sharma, 2016**

**Synonyms.** None.

**Holotype.** Improperly designated (see Remarks).

**Type locality.** South Africa, Mpumalanga, Highveld, Mount Sheba Nature Reserve, 24°56.33'S, 30°42.81'E, ca 1,680 m ASL (see Remarks).

**Language of species description.** English.

**Remarks.** Three specimens have been designated as holotype in the original publication (Daniels et al. 2016: 534). Since the ICZN establishes that the holotype consists of “one, and only one, specimen” (Art. 73.1), the fixation of the name-bearing type, in this case, must be regarded as invalid. According to the availability criteria, however, new specific names published after 1999 must be accompanied in the original publication by the explicit fixation of a holotype, or syntypes, for the nominal taxon (ICZN Art. 16.4.1). To avoid nomenclatural instability by declaring *Opisthopatusswatii* a nomen nudum and, following advice from members of the International Committee of Zoological Nomenclature, the three specimens deposited in the Entomological Collection of the South African Museum (Iziko Museums of Cape Town), Cape Town, South Africa, under the accession number SAM-ENW-C007169 should now be regarded as syntypes. This would be in line with the criteria established by the ICZN (Art. 73.2.1.1) for syntype designation, which says “(w)hen a nominal taxon is established after 1999, only those specimens expressly indicated by the author as those upon which the new taxon is based (see Article 72.3) are syntypes”. Accordingly, the 28 specimens previously regarded as paratypes (SAM-ENW-C007170) must be excluded from the type series, as paratypes, per se, do not represent name-bearing types (ICZN 73.1); these specimens should now be regarded as additional material. Altitude data obtained from Google Earth based on the given geographic coordinates. Geographic coordinates presented in ‘table 1 [List of sample localities]’ by Daniels et al. (2016: 510) may contain a typo (minus sign associated with longitude rather than latitude); correct and more precise coordinates provided along the species description. Species described based on morphological and molecular data. The species description includes specimens from different localities lying up to 99 km away from the type locality, hence the existence of a species complex within *Opisthopatusswatii* cannot be excluded. For the sake of caution, the name *Opisthopatusswatii* should only be applied to specimens from the type locality. Requires revision.

**Nomina dubia**

**nd2. *Opisthopatusamatolensis* Choonoo, 1947**

**Synonyms.**Opisthopatuscinctipesvar.amatolensis (Choonoo 1947: 72); *Opisthopatusamatolensis* (Oliveira et al. 2012a: 43).

**Holotype.** Not designated (see Remarks).

**Type locality.** South Africa, Eastern Cape. The precise locality might be southeast of Houghton’s farm, along the road from Alice towards Hogsback, ca 1,160 m [3,800 ft] ASL (Choonoo 1947: 71).

**Language of species description.** English.

**Remarks.** The holotype has not been designated explicitly in the original description. The variety *amatolensis* had previously been considered as invalid by Ruhberg (1985: 85) and Hamer et al. (1997: 292). However, Oliveira et al. (2012a: 43) suggested that the great distance between the type localities of *Opisthopatusamatolensis* and *Opisthopatuscinctipes* (161 km) provides sufficient evidence for ruling out conspecificity and raised this variety to species status. For the sake of caution, *Opisthopatusamatolensis* is herein treated as a nomen dubium until further investigations are conducted.

**nd3. *Opisthopatuslaevis* Lawrence, 1947**

**Synonyms.**Opisthopatuscinctipesvar.laevis as originally described (Lawrence 1947: 168); *Opisthopatuslaevis* (Oliveira et al. 2012a: 44).

**Holotype.** Not designated (see Remarks).

**Type locality.** South Africa, KwaZulu-Natal, East Griqualand, Bulwer.

**Language of species description.** English.

**Remarks.** The holotype has not been designated explicitly in the original description. The variety *laevis* had previously been considered as invalid by Ruhberg (1985: 85) and Hamer et al. (1997: 292). However, Oliveira et al. (2012a: 44) suggested that the great distance between the type localities of *Opisthopatuslaevis* and *Opisthopatuscinctipes* (571 km) provides sufficient evidence for ruling out conspecificity and raised this variety to species status. For the sake of caution, *Opisthopatuslaevis* is herein treated as a nomen dubium until further investigations are conducted.

**nd4. *Opisthopatusnatalensis* Bouvier, 1900d**

**Synonyms.**Opisthopatuscinctipesvar.natalensis, as originally described (Bouvier 1900d: 368); *Opisthopatusnatalensis* (Oliveira et al. 2012a: 44).

**Holotype.** Not designated (see Remarks).

**Type locality.** South Africa, Kwa-Zulu-Natal, Durban.

**Language of species description.** French.

**Remarks.** The holotype has not been designated explicitly in the original description. Bouvier (1907a: 520) refers to a cotype [obsolete for syntype] deposited in the Museum National d’Histoire Naturelle de Paris, France (see also Le Bras et al. 2015). The variety *natalensis* had previously been considered as invalid by Ruhberg (1985: 85) and Hamer et al. (1997: 292). However, Oliveira et al. (2012a: 44) suggested that the great distance between the type localities of *Opisthopatusnatalensis* and *Opisthopatuscinctipes* (656 km) provides sufficient evidence for ruling out conspecificity and raised this variety to species status. For the sake of caution, *Opisthopatusnatalensis* is herein treated as a nomen dubium until further investigations are conducted.

##### XXIX. *Paraperipatus* Willey, 1898a

**Type species.***Paraperipatusnovaebritanniae* (Willey, 1898b), by monotypy (Willey 1898a: 4).

**Remarks.***Paraperipatus* was initially introduced as a subgenus of *Peripatus* (see Willey 1898a: 3) but it is commonly treated at the generic rank in the literature (e.g., Bouvier 1900d: 369; Ruhberg 1985: 142; Reid 1996: 902). To prevent taxonomic instability, *Paraperipatus* is regarded herein as a valid genus. Most species of the genus are understudied and require thorough morphological and molecular revision.

**73. *Paraperipatusceramensis* (Muir & Kershaw, 1909)**

**Synonyms.***Peripatusceramensis*, as originally described (Muir and Kershaw 1909: 737); *Paraperipatusceramensis* (Horst 1910: 218).

**Holotype.** Not designated (see Remarks).

**Type locality.** Republic of Indonesia, Maluku Islands, West Seram, vicinity of Përoe (see Remarks).

**Language of species description.** English.

**Remarks.** The holotype has not been designated explicitly in the original description. Syntypes are deposited in the Museum of Comparative Zoology at Harvard University, Cambridge, USA, and in the Museum National d’Histoire Naturelle de Paris, France (see Le Bras et al. 2015 for the latter). The locality cited as Përoe by Muir and Kershaw (1909: 740) could be on the Seram island. Requires morphological revision and molecular characterization.

**74. *Paraperipatuskeiensis* Horst, 1923**

**Synonyms.** None.

**Holotype.** Not designated (see Remarks).

**Type locality.** Republic of Indonesia, Kai Islands, Great Kai Island [Kai Besar], Gunung Daab, ca 300 m ASL. Originally described as “Goenoeng Daab, Great Kei, at the height of about 300 m” (Horst, 1923: 119).

**Language of species description.** English.

**Remarks.** The species name has been misspelt as *Paraper.keyensis* [sic] in the original description (Horst 1923: 119). The holotype has not been designated explicitly in the original description. Requires morphological revision and molecular characterization.

**75. *Paraperipatuslorentzi* Horst, 1910**

**Synonyms.** None (see Remarks).

**Holotype.** Not designated (see Remarks)

**Type locality.** Republic of Indonesia, Western New Guinea, West Papua, Wichmann Mountains, southern part of the Arfak Range, ca 2,743 [9,000 ft] ASL (see Remarks).

**Language of species description.** English.

**Remarks.** The species has previously been synonymized with *Paraperipatuspapuensis* (Ruhberg 1985: 151). Oliveira et al. (2012a: 46), however, regarded *Paraperipatuslorentzi* as a separate species, since it differs morphologically from *Paraperipatuspapuensis* and is unlikely to show an overlapping distribution with the latter (Brues 1921: 51, 52). The holotype has not been designated explicitly in the original description. Hence, the specimens referred by Brues (1921: 51) as types correspond to syntypes. The location of the syntypes is unclear: at the time the species was described, the author was hosted at the Leyden Museum [currently Naturalis Biodiversity Center] in Leiden, Netherlands. However, these specimens are currently not listed in their online database. Additional information on the type locality has been provided by Brues (1921: 51). Molecular sequences provided by Giribet et al. (2018) under the name Paraperipatuscf.lorentzi are likely to belong to this species. Requires revision.

**76. *Paraperipatusnovaebritanniae* (Willey, 1898b)**

**Synonyms.***Peripatusnovæ-britanniæ*, as originally described (Willey 1898b: 286); *Peripatus (Paraperipatus) novæ-britanniæ* (Willey 1898a: 4); *Paraperipatusnovæ-britanniæ* (Bouvier 1900d: 369); *Paraperipatusnovaebritanniae* (Oliveira et al. 2012a: 45) (see Remarks).

**Holotype.** Not designated (see Remarks).

**Type locality.** Papua New Guinea, Bismarck archipelago, New Britain Island, Gazelle Peninsula, Blanche Bay, Karavi. Originally described as “at an elevation of several hundred feet above see-level … in the immediate vicinity of a fresh water source and in the gully in which the stream from the source flowed, in the hills behind the native village of Karavia which lies at the head of Blanche Bay” (Willey 1898a: 2).

**Language of species description.** English.

**Remarks.** Specific epithet originally spelt *novæ-britanniæ* (Willey 1898b: 286). According to the ICZN, however, “(n)o diacritic or other mark (such as an apostrophe), or ligature of the letters a and e (æ) … is to be used in a scientific name” (Art. 27), and “(a) name published with a diacritic or other mark, ligature, apostrophe, or hyphen … is to be corrected” (Art. 32.5.2). Thus, the specific epithet has been corrected to *novaebritanniae* by Oliveira et al. (2012a: 45). The holotype has not been designated explicitly in the original description. Syntype are deposited in the Museum of Comparative Zoology at Harvard University, Cambridge, USA. Bouvier (1907a: 520) also refers to a cotype [obsolete for syntype] deposited in the Museum National d’Histoire Naturelle de Paris, France (see also Le Bras et al. 2015). The species name was first introduced by Willey (1898b) as a brief note, whereas a more comprehensive species description followed in a separate publication (Willey 1898a). Requires morphological revision and molecular characterization.

**77. *Paraperipatuspapuensis* (Sedgwick, 1910)**

**Synonyms.***Peripatuspapuensis*, as originally described (Sedgwick 1910: 369); *Paraperipatuspapuensis* (Bouvier 1914b: 222); *Paraperipatusleopoldi* Leloup, 1931 (objective synonym; Ruhberg 1985: 151; see Comments on *Paraperipatusleopoldi* below) (see Remarks).

**Holotype.** Not designated (see Remarks).

**Type locality.** Republic of Indonesia, Western New Guinea, West Papua, Sarayu, Central Arfak Mountains, ca 1,065 m [3,500 ft] ASL.

**Language of species description.** English.

**Remarks.** Comments on the objective synonym *Paraperipatusleopoldi* Leloup, 1931 are provided along with the nomina dubia below. The holotype has not been designated explicitly in the original description. Syntypes are deposited in the Museum of Comparative Zoology at Harvard University, Cambridge, USA, and in the Museum National d’Histoire Naturelle de Paris, France (see Le Bras et al. 2015 for the latter). Requires morphological revision and molecular characterization.

**78. *Paraperipatusvanheurni* Horst, 1922**

**Synonyms.** None.

**Holotype.** Not designated (see Remarks).

**Type locality.** Republic of Indonesia, Western New Guinea, Papua, Maoke Mountains, ca 2,900 m ASL. Originally described as “Doormanpad, New Guinea” (Horst 1922: 113) (see Remarks).

**Language of species description.** English.

**Remarks.** The holotype has not been designated explicitly in the original description. The current name of Doormanpad, or Doormanpadbivak, might be Maoke Mountains [= Pegunungan Maoke]. The locality has erroneously been assigned to New Guinea by Oliveira et al. (2012a: 47), but according to Schileyko and Stoev (2016: 262) it is located “in Indonesian Province Papua at upper Lorentz River in the Snow Mountains, 03°30'S, 138°30'E”. Requires morphological revision and molecular characterization.

**Nomina dubia**

**nd5. *Paraperipatusamboinensis* Pflugfelder, 1948**

**Synonyms.** None.

**Holotype.** Not designated (see Remarks).

**Type locality.** Republic of Indonesia, Maluku Islands, Ambon Island.

**Language of species description.** German.

**Remarks.** The holotype has not been designated explicitly in the original description. According to Ruhberg (1985: 146), the syntypes of this species are lost. The description contains imprecise type locality data: Ambon Island covers an area of 775 km^2^. Oliveira et al. (2012a: 46) regarded *Paraperipatusamboinensis* as a nomen dubium since type material is unknown and precise locality data are missing in the literature, thus precluding an unambiguous revision of this species based on topotypes.

**nd6. *Paraperipatusschultzei* Heymons, 1912**

**Synonyms.**Paraperipatusschultzeivar.ferrugineus (Heymons 1912: 216) (see Remarks).

**Holotype.** Not designated (see Remarks).

**Type locality.** Papua New Guinea, north of New Guinea, Sepik River system, ca 1,570 m ASL. Originally described as “inland region, on a mountain at 1,570 m” (Heymons 1912: 215) (see Remarks).

**Language of species description.** German.

**Remarks.** The holotype has not been designated explicitly in the original description. A (syn)type specimen previously deposited in the Museum für Naturkunde Berlin, Germany, is believed to be lost (Röhlig et al. 2010: 228; Oliveira et al. 2012a: 47). A more precise locality for this species is provided in Röhlig et al. (2010: 228). A putative variety, Paraperipatusschultzeivar.ferrugineus, has been described from the same locality based on color differences and a distinct number of leg pairs (Heymons 1912: 216), but since these characters are known to vary intraspecifically, Ruhberg (1985: 153) regarded Paraperipatusschultzeivar.ferrugineus as synonym of *Paraperipatusschultzei*. Oliveira et al. (2012a:47) regarded *Paraperipatusschultzei* as a nomen dubium since type material is unknown and precise locality data are missing in the literature, thus precluding an unambiguous revision of this species based on topotypes.

**nd7. *Paraperipatusstresemanni* Bouvier, 1914b**

**Synonyms.** None.

**Holotype.** Not designated (see Remarks).

**Type locality.** Republic of Indonesia, inland region of Seram Island (see Remarks).

**Language of species description.** French.

**Remarks.** The species name has been misspelt as *stresemani* [sic] by Leloup (1931: 6). The holotype has not been designated explicitly in the original description. A syntype is deposited in the Museum National d’Histoire Naturelle de Paris, France (see Le Bras et al. 2015). The description contains imprecise locality data: The Seram Island occupies an area of ~ 17,100 km^2^. Oliveira et al. (2012a:47) regarded *Paraperipatusstresemanni* as a nomen dubium since precise locality data are missing in the literature, thus precluding an unambiguous revision of this species based on topotypes.

**Comments on *Paraperipatusleopoldi* Leloup, 1931**

The *Paraperipatusleopoldi* has been described by Leloup (1931) for the Republic of Indonesia (Western New Guinea, West Papua, environs of Sakaoeni, 500 m). Besides the imprecise locality data and lack type designation in the original description, taxonomy of *Paraperipatusleopoldi* is marked by numerous twists and turns. In the original species description, Leloup (1931: 12, 13) overlooked the principle of priority (see ICZN Art. 23) and regarded six additional species of *Paraperipatus* as intermediate forms [formes intermédiaires] of *Paraperipatusleopoldi*, namely *Paraperipatuslorentzi*, *Paraperipatusvanheurni*, *Paraperipatuskeiensis*, *Paraperipatusstresemanni*, *Paraperipatusschultzei* and *Paraperipatuspapuensis*. This mistake has subsequently been pointed out by Brongersma (1932: 411), who suggested that the name *Paraperipatusleopoldi* needed to be suppressed and all species interpreted as intermediate forms should rather be treated under the oldest name, i.e., *Paraperipatuspapuensis*. Many decades later, Ruhberg (1985: 151) reverted this synonymization by considering *Paraperipatuslorentzi*, *Paraperipatusvanheurni*, *Paraperipatuskeiensis*, *Paraperipatusstresemanni*, *Paraperipatusschultzei* and *Paraperipatuspapuensis* as valid names. Ruhberg (1985: 151) also designated the syntype of *Paraperipatuspapuensis* as the type of *Paraperipatusleopoldi*, thus declaring the latter as objective synonym of *Paraperipatuspapuensis*. Oliveira et al. (2012a: 47), however, overlooked the objective synonymization and rather regarded *Paraperipatusleopoldi* as a nomen dubium, since this species was found ~ 145 km apart from *Paraperipatuspapuensis* and conspecificity, in this case, would be unlikely. For the sake of clarity, the name *Paraperipatusleopoldi* must now be treated as objective synonym of *Paraperipatuspapuensis*, while the species occurring in the environs of Sakaoeni (West Papua) needs to be assigned with a valid species name accompanied by proper type designation.

##### XXX. *Paropisthopatus* Ruhberg, 1985

**Type species.***Paropisthopatusumbrinus* (Johow, 1911), by original designation (Ruhberg 1985: 110).

**Remarks.** Requires molecular characterization.

**79. *Paropisthopatusumbrinus* (Johow, 1911)**

**Synonyms.**Peripatus (Peripatopsis) umbrinus, as originally described (Johow 1911: 84); *Metaperipatusumbrinus* (Clark 1915: 21; Peck 1975: 344); *Paropisthopatusumbrinus* (Ruhberg 1985: 111).

**Holotype.** Not designated (see Remarks).

**Type locality.** Chile, Valparaíso, Balneario de Zapallar, near the border of the Aconcagua province (32°33'S), La Higuera mountain, Quebrada del Tigre (300–500 m), and a ‘point situated next to the top’ (700 m) (see Remarks).

**Language of species description.** Spanish.

**Remarks.** The holotype has not been designated explicitly in the original description. Based on the latitude, altitude and locality data originally provided, the type locality might currently correspond to the El Boldo Park [Zapallar], at approximately 32°33'S and 71°26.39'W. Requires morphological revision and molecular characterization.

**Nomen dubium**

**nd8. *Paropisthopatuscostesi* (Gravier & Fage, 1925)**

**Synonyms.***Opisthopatuscostesi*, as originally described (Gravier and Fage 1925: 194); *Metaperipatuscostesi* (Peck 1975: 344); *Paropisthopatuscostesi* (Ruhberg 1985: 111).

**Holotype.** Not designated (see Remarks).

**Type locality.** Chile, Colchagua (see Remarks).

**Language of species description.** French.

**Remarks.** The holotype has not been designated explicitly in the original description. The description contains imprecise locality data: the province of Colchagua in Chile occupies 5,678 km^2^. Ruhberg (1985: 111) considered this species as uncertain, and Oliveira et al. (2012a: 48) declared *Paropisthopatuscostesi* as a nomen dubium since type material is unknown and precise locality data are missing in the literature, thus precluding an unambiguous revision of this species based on topotypes.

##### XXXI. *Peripatoides* Pocock, 1894

**Type species.***Peripatoidesnovaezealandiae* (Hutton, 1876), by original designation (Pocock 1894: 519).

**80. *Peripatoidesaurorbis* Trewick, 1998**

**Synonyms.** None.

**Holotype.** Deposited in the Museum of New Zealand [Te Papa Tongarewa], Wellington, New Zealand. Holotype designated ambiguously in the original species description (see Remarks).

**Type locality.** New Zealand, North Island, Kawau Island (see Remarks).

**Language of species description.** English.

**Remarks.** Species diagnosed based on molecular data only. Although the author explicitly demonstrated his intention of designating the holotype (Trewick 1998: 322), the information originally provided is insufficient to identify the name-bearing type unambiguously: the author only writes ‘Holotype Kawau Island’, a locality from which nine specimens have been obtained for the study (Trewick 1998: 309). Since neither the sex, nor the depository institution, nor the accession number of the holotype has been specified, its location and identity remained unclear. However, the online database of the Museum of New Zealand [Te Papa Tongarewa] indicates that the holotype has been deposited in this institution under the accession number AI.012621. According to the ICZN, nominal species-group taxa established before 2000 “may have its name-bearing type fixed from the type series originally, or subsequently” (Art. 72.2) and “any evidence, published or unpublished, may be taken into account to determine what specimens constitute the type series” (Art. 72.4.1.1). Hence, *Peripatoidesaurorbis* is herein regarded as a valid species, as opposed to its previous designation as nomen dubium (Oliveira et al. 2012a: 49). This species has been described based on specimens from three different areas lying 95–250 km away from the type locality. Also, additional molecular data (Murienne et al. 2014; Giribet et al. 2018) including transcriptome (Laumer et al. 2019; Baker et al. 2021) recently assigned to *Peripatoidesaurorbis*, may not strictly correspond to the original species, as they were obtained from specimens collected 157–559 km away from the type locality. Hence, the existence of a species complex within *Peripatoidesaurorbis* cannot be excluded. For the sake of caution, the name *Peripatoidesaurorbis* should only be applied to specimens from the type locality. The geographic coordinates originally provided by Trewick (1998: 309) for the type locality [36°30'S, 174°40'E] do not seem to correspond to the position of the Kawau Island. Requires revision.

**81. *Peripatoidesindigo* Ruhberg, 1985**

**Synonyms.** None.

**Holotype.** Deposited in the Entomology Division of the Department of Scientific and Industrial Research, Auckland, New Zealand.

**Type locality.** New Zealand, South Island, Nelson district, Bainham Paturau, Twin Forks Cave, 3.22 km [2 miles] south of the Paturau River and 1.6 km [1 mile] inland from the coast.

**Language of species description.** German.

**Remarks.** Requires morphological revision and molecular characterization.

**82. *Peripatoideskawekaensis* Trewick, 1998**

**Synonyms.** None.

**Holotype.** Deposited in the Museum of New Zealand [Te Papa Tongarewa], Wellington, New Zealand. Holotype designated ambiguously in the original species description (see Remarks).

**Type locality.** New Zealand, North Island, Hawke’s Bay, Hutchinson Reserve (see Remarks).

**Language of species description.** English.

**Remarks.** Species diagnosed based on molecular data only. Although the author explicitly demonstrated his intention of designating the holotype (Trewick 1998: 322), the information originally provided is insufficient to identify the name-bearing type unambiguously: the author only writes ‘Holotype Hutchinson Reserve’, a locality from which three specimens have been obtained for the study (Trewick 1998: 309). Since neither the sex, nor the depository institution, nor the accession number of the holotype has been specified, its location and identity remained unclear. However, the online database of the Museum of New Zealand [Te Papa Tongarewa] indicates that the holotype has been deposited in this institution under the accession number AI.012622. According to the ICZN, nominal species-group taxa established before 2000 “may have its name-bearing type fixed from the type series originally, or subsequently” (Art. 72.2) and “any evidence, published or unpublished, may be taken into account to determine what specimens constitute the type series” (Art. 72.4.1.1). As opposed to other species described by Trewick (1998), *Peripatoideskawekaensis* has previously been regarded as valid by Oliveira et al. (2012a: 48) due to its restricted distribution. The geographic coordinates originally provided by Trewick (1998: 309) for the type locality [39°16'S, 176°32'E] do not seem to correspond to the position of the Hutchinson Reserve. Requires revision.

**83. *Peripatoidesmorgani* Trewick, 1998**

**Synonyms.** None.

**Holotype.** Deposited in the Museum of New Zealand [Te Papa Tongarewa], Wellington, New Zealand. Holotype designated ambiguously in the original species description (see Remarks).

**Type locality.** New Zealand, North Island, Mohi Bush Scenic Reserve (see Remarks).

**Language of species description.** English.

**Remarks.** Species diagnosed based on molecular data only. Although the author explicitly demonstrated his intention of designating the holotype (Trewick 1998: 321), the information originally provided is insufficient to identify the name-bearing type unambiguously: the author only writes ‘Holotype Mohi Bush’, a locality from which five specimens have been obtained for the study (Trewick 1998: 309). Since neither the sex, nor the depository institution, nor the accession number of the holotype has been specified, its location and identity remained unclear. However, the online database of the Museum of New Zealand [Te Papa Tongarewa] indicates that the holotype has been deposited in this institution under the accession number AI.012623. According to the ICZN, nominal species-group taxa established before 2000 “may have its name-bearing type fixed from the type series originally, or subsequently” (Art. 72.2) and “any evidence, published or unpublished, may be taken into account to determine what specimens constitute the type series” (Art. 72.4.1.1). Hence, *Peripatoidesmorgani* is herein regarded as a valid species, as opposed to its previous designation as nomen dubium (Oliveira et al. 2012a: 49). This species has been described based on specimens from seven different areas lying 30–190 km away from the type locality. Hence, the existence of a species complex within *Peripatoidesmorgani* cannot be excluded. For the sake of caution, the name *Peripatoidesmorgani* should only be applied to specimens from the type locality. The geographic coordinates originally provided by Trewick (1998: 309) for the type locality [39°35'S, 177°05'E] do not seem to correspond to the position of the Mohi Bush Scenic Reserve. Requires revision.

**84. *Peripatoidesnovaezealandiae* (Hutton, 1876)**

**Synonyms.***Peripatusnovæ-zealandiæ*, as originally described (Hutton 1876: 361); *Peripatoidesnovæ-zealandiæ* (Pocock 1894: 519); *Peripatoidesnovaezealandiae* (Watt 1961: 97) (see Remarks).

**Holotype.** Not designated (see Remarks). Neotype deposited in the Museum of New Zealand [Te Papa Tongarewa], Wellington, New Zealand. Neotype designated ambiguously in the original species description (see Remarks).

**Type locality.** New Zealand, North Island, Wellington, Ōtari-Wilton’s Bush Native Botanic Garden (see Remarks). Originally described as “Otari Plant Museum” (Trewick 1998: 309, 321).

**Language of species description.** English.

**Remarks.** Specific epithet originally spelt *novæ-zealandiæ* (Hutton 1876: 361). According to the ICZN, however, “(n)o diacritic or other mark (such as an apostrophe), or ligature of the letters a and e (æ) … is to be used in a scientific name” (Art. 27), and “(a) name published with a diacritic or other mark, ligature, apostrophe, or hyphen … is to be corrected” (Art. 32.5.2). Thus, the correct spelling for the specific epithet is *novaezealandiae* (e.g., Watt 1961; Ruhberg 1985). The species name was previously used for every onychophoran species with 15 leg pairs found in New Zealand (Ruhberg 1985: 138–140; Trewick 1998: 309). The original species description was based on specimens from different localities on both North Island and South Island. Trewick (1998: 321) subsequently restricted the distribution of this species to the southern part of the North Island and, since the holotype has not been designated explicitly in the original description, a neotype from the Ōtari-Wilton’s Bush Native Botanic Garden has been elected. However, the information provided by Trewick is insufficient to identify the name-bearing type unambiguously: the author only writes ‘Neotype Otari’, a locality from which five specimens have been obtained for the study (Trewick 1998: 309). Since neither the sex, nor the depository institution, nor the accession number of the neotype has been specified, its location and identity remained unclear. According to the online database of the Museum of New Zealand [Te Papa Tongarewa], the neotype has been deposited in this institution under the accession number AI.012624. Considering that nominal species-group taxa established before 2000 “may have its name-bearing type fixed from the type series originally, or subsequently” (ICZN Art. 72.2) and “any evidence, published or unpublished, may be taken into account to determine what specimens constitute the type series” (ICZN Art. 72.4.1.1), *Peripatoidesnovaezealandiae* is herein regarded as a valid species, as opposed to its previous designation as nomen dubium (Oliveira et al. 2012a: 49). This species has been identified from six different areas lying 46–128 km away from the type locality. Additional molecular data available for *Peripatoidesnovaezealandiae* were mainly obtained from topotypes (e.g., Murienne et al. 2014; Giribet et al. 2018), although one specimen (DNA103694) identified as such by Murienne et al. (2014) was collected 487 km away from the type locality and may not strictly correspond to the original species. Hence, the existence of a species complex within *Peripatoidesnovaezealandiae* still cannot be excluded. For the sake of caution, the name *Peripatoidesnovaezealandiae* should only be applied to specimens from the type locality. The geographic coordinates originally provided by Trewick (1998: 309) for the type locality [41°6'S, 174°45'E] do not seem to correspond to the position of the Ōtari-Wilton’s Bush Native Botanic Garden. Requires revision.

**85. *Peripatoidessuteri* (Dendy, 1894)**

**Synonyms.**Peripatusnovæ-zealandiævar.suteri, as originally described (Dendy 1894: 401); *Peripatussuteri* (Dendy 1900c: 444); *Peripatoidessuteri* (Bouvier 1901b: 60).

**Holotype.** Not designated (see Remarks).

**Type locality.** New Zealand, North Island, Stratford.

**Language of species description.** English.

**Remarks.** The holotype has not been designated explicitly in the original description. Bouvier (1907a: 520) refers to a cotype [obsolete for syntype] deposited in the Museum National d’Histoire Naturelle de Paris, France (see also Le Bras et al. 2015). Ruhberg (1985: 142) refers to an additional ‘type’ specimen deposited in the Natural History Museum of London, UK, which should be regarded as syntype, as well as to syntypes deposited in the Canterbury Museum, Christchurch, New Zealand, and Zoologisches Museum, University of Hamburg, Hamburg, Germany. Molecular data assigned to *Peripatoidessuteri* (see Trewick 1998, 2000; Giribet et al. 2018) may not strictly correspond to the original species, as the sequenced specimens were collected up to 276 km away from the type locality. Hence, the existence of a species complex within *Peripatoidessuteri* cannot be excluded. For the sake of caution, the name *Peripatoidessuteri* should only be applied to specimens from the type locality. Requires revision.

**86. *Peripatoidessympatrica* Trewick, 1998**

**Synonyms.** None.

**Holotype.** Deposited in the Museum of New Zealand [Te Papa Tongarewa], Wellington, New Zealand. Holotype designated ambiguously in the original species description (see Remarks).

**Type locality.** New Zealand, North Island, Norsewood, ANZAC Park Scenic Reserve (see Remarks). Originally described as ANZAC Reserve, Norsewood (Trewick 1998: 309).

**Language of species description.** English.

**Remarks.** Species diagnosed based on molecular data only. Although the author explicitly demonstrated his intention of designating the holotype (Trewick 1998: 322), the information originally provided is insufficient to identify the name-bearing type unambiguously: the author only writes ‘Holotype Norsewood’, a locality from which eight specimens have been obtained for the study (Trewick 1998: 309). Since neither the sex, nor the depository institution, nor the accession number of the holotype has been specified, its location and identity remained unclear. However, the online database of the Museum of New Zealand [Te Papa Tongarewa] indicates that the holotype has been deposited in this institution under the accession number AI.012625. According to the ICZN, nominal species-group taxa established before 2000 “may have its name-bearing type fixed from the type series originally, or subsequently” (Art. 72.2) and “any evidence, published or unpublished, may be taken into account to determine what specimens constitute the type series” (Art. 72.4.1.1). Hence, *Peripatoidessympatrica* is herein regarded as a valid species, as opposed to its previous designation as nomen dubium (Oliveira et al. 2012a: 50). This species has been described based on specimens from nine different areas lying 45–342 km away from the type locality. Also, additional molecular data (Murienne et al. 2014; Giribet et al. 2018) including a complete mitochondrial genome (Segovia et al. 2011) recently assigned to *Peripatoidessympatrica*, may not strictly correspond to the original species, as they were obtained from specimens collected 292–692 km away from the type locality. Hence, the existence of a species complex within *Peripatoidessympatrica* cannot be excluded. For the sake of caution, the name *Peripatoidessympatrica* should only be applied to specimens from the type locality. The geographic coordinates originally provided by Trewick (1998: 309) for the type locality [40°52'S, 176°13'E] do not seem to correspond to the position of the ANZAC Park Scenic Reserve. Requires revision.

##### XXXII. *Peripatopsis* Pocock, 1894

**Type species.***Peripatopsiscapensis* (Grube, 1866), by original designation (Pocock 1894: 519).

**87. *Peripatopsisalba* Lawrence, 1931**

**Synonyms.** None.

**Holotype.** Not designated (see Remarks).

**Type locality.** South Africa, Cape Town, Table Mountain Nature Reserve, Table Mountain Caves, in sandstone formation near the top of Table Mountain.

**Language of species description.** English.

**Remarks.** The species name has been misspelt as *P.albida* [sic] in Robson (1964: 284). Lawrence (1931: 104) designated two ‘types’ in the original description but did not specify explicitly which one of them is the holotype. Hence, both specimens should be regarded as syntypes. Lawrence (1931) also did not indicate the depository institution for the syntypes, but it is likely that these specimens have been placed in the Entomological Collection of the South African Museum (Iziko Museums of Cape Town), Cape Town, South Africa, as the author used to work at this institution when the species was described. Molecular data available for this species (Daniels et al. 2013). Requires revision.

**88. *Peripatopsisbalfouri* (Sedgwick, 1885)**

**Synonyms.***Peripatusbalfouri*, as originally described (Sedgwick 1885: 450); *Peripatopsisbalfouri* (Purcell 1899: 341).

**Holotype.** Not designated (see Remarks). Neotype deposited in the Entomological Collection of the South African Museum (Iziko Museums of Cape Town), Cape Town, South Africa.

**Type locality.** South Africa, Western Cape, Cape Peninsula, Cape Point, Booi se Skerm, 34°18.27'S, 18°27.60'E, ca 55 m ASL (see Remark). Originally described as “Cape” by Sedgwick (1885: 451) and subsequently as “Table Mountain” by the same author (Sedgwick 1888: 440).

**Language of species description.** English.

**Remarks.** The holotype has not been designated explicitly in the original description. According to Ruhberg (1985: 92), the location of the material originally studied by Sedgwick could not be identified. *Peripatopsisbalfouri* has previously been interpreted as a species complex (Daniels et al. 2009), which has subsequently been split into different species based on molecular data (Daniels et al. 2013). A neotype has been designated by Daniels et al. (2013: 670) and deposited in the South African Museum of Natural History, Iziko Museum of Cape Town (South Africa). The neotype designation fixes the Booi se Skerm as the species type locality (altitude data obtained from Google Earth based on the given geographic coordinates). *Peripatopsisbalfouri* sensu stricto, as defined by Daniels et al. (2013), encloses specimens from nine additional areas lying 20–89 km apart from the type locality. Hence, the existence of a species complex within *Peripatopsisbalfouri* still cannot be excluded. For the sake of caution, the name *Peripatopsisbalfouri* should only be applied to specimens from the type locality. Requires revision.

**89. *Peripatopsisbirgeri* Ruhberg & Daniels, 2013**

**Synonyms.** None.

**Holotype.** Deposited in the Entomological Collection of the South African Museum (Iziko Museums of Cape Town), Cape Town, South Africa.

**Type locality.** South Africa, KwaZulu-Natal, Mount Currie Nature Reserve, 30°28.73'S, 29°22'E, ca 1,695 m ASL (see Remarks).

**Language of species description.** English.

**Remarks.** Altitude data obtained from Google Earth based on the given geographic coordinates. The species description includes morphological and molecular data. According to the original authors (Ruhberg and Daniels 2013: 141), *Peripatopsisbirgeri* could constitute a species complex, which has recently been revised and split into two different species (Grobler et al. 2023). Yet, *Peripatopsisbirgeri* is still recorded from several areas lying up to 90 km from the type locality. Given the presence of distinct monophyletic units within this clade (Grobler et al. 2023: 7), the existence of a species complex within this species can still not be excluded. For the sake of caution, the name *Peripatopsisbirgeri* should only be applied to specimens from the type locality. Geographic coordinates presented by Grobler et al. (2023) may contain a typo (DDM values presented in DMS format without proper conversion).

**90. *Peripatopsisbolandi* Daniels, McDonald & Picker, 2013**

**Synonyms.** None.

**Holotype.** Deposited in the Entomological Collection of the South African Museum (Iziko Museums of Cape Town), Cape Town, South Africa.

**Type locality.** South Africa, Western Cape, Hottentots Holland mountains, Simonberg, 33°53.52'S, 18°55.42'E, ca 690 m ASL (see Remarks).

**Language of species description.** English.

**Remarks.** Altitude data obtained from Google Earth based on the given geographic coordinates. The species description includes morphological and molecular data. Transcriptome data recently assigned to *Peripatopsisbolandi* were obtained from a specimen collected 15 km away from the type locality (Baker et al. 2021). According to the original authors (Daniels et al. 2013: 672), *Peripatopsisbolandi* may constitute a species complex, which requires revision. For the sake of caution, the name *Peripatopsisbolandi* should only be applied to specimens from the type locality.

**91. *Peripatopsiscapensis* (Grube, 1866)**

**Synonyms.***Peripatusbrevis* de Blainville in Gervais, 1836 (nomen nudum; Gervais 1836: XV); *Peripatusbrevis* de Blainville in Gervais, 1837 (senior synonym, nomen oblitum; de Blainville in litt., footnote in Gervais 1837: 38); *Peripatuscapensis* Grube, 1866 (junior synonym, nomen protectum; Grube 1866: 65); *Peripatopsiscapensis* (Pocock 1894: 519) (see Remarks).

**Holotype.** Not designated. Neotype deposited in the Zoologisches Museum, Hamburg, Germany (see Remarks).

**Type locality.** South Africa, Cape Town, Table Mountain, Rhodes Memorial (see Remarks).

**Language of species description.** German.

**Remarks.***Peripatusbrevis* represents the second onychophoran species ever described. The name first appears in a communication by Gervais (1836: XV) to the Bulletin Entomologique (published along with the Annales de Société Entomologique de France) citing the discovery of M. de Blainville. Since this publication does not include a proper description, it fails to conform to the provisions of the ICZN (Art. 12). Hence, *Peripatusbrevis* de Blainville in Gervais, 1836 is a nomen nudum. The name *Peripatusbrevis* only became available a year later (Gervais 1837: 38, footnote). The publication by de Blainville in the Comptes Rendus Hebdomadaires des Séances de l’académie des Sciences (de Blainville 1837: 147) should not be considered for nomenclatural purposes, as it does not include a proper description of *Peripatusbrevis* and has been published seven months after Gervais (1837). Over the years, the identity of this species has been questioned due to its vague description, with most authors suggesting that *Peripatusbrevis* and *Peripatuscapensis* could represent one single species (e.g., Moseley 1874: 759; Bouvier 1901c: 74–75, 1907b: 145–150; Ruhberg 1985: 94). Although *Peripatusbrevis* most likely represents the senior synonym of *Peripatuscapensis*, the latter has long been used in the literature and its replacement has been avoided for the sake of nomenclatural stability (Bouvier 1901c; Ruhberg 1985). Since *Peripatusbrevis* de Blainville in Gervais, 1837 has not been used as a valid name after 1899 and *Peripatuscapensis* Grube, 1866 has broadly become the presumed valid name for the species, the former should now be regarded as nomen oblitum and the latter as nomen protectum, following the rules of the ICZN (Art. 23.9.1). The holotype of *Peripatopsiscapensis* has not been designated explicitly in the original description. Since Bouvier (1907b: 146) stated that the syntypes have been lost, Ruhberg (1985: 94) designated a male specimen found at the Rhodes Memorial (Table Mountain, Cape Town, South Africa) as neotype, which has been deposited in the Zoologisches Museum, Hamburg, Germany. *Peripatopsiscapensis* was regarded as a species complex by Daniels et al. (2009) and subsequently split into different species using morphological and molecular methods (McDonald et al. 2012).

**92. *Peripatopsiscederbergiensis* Daniels, McDonald & Picker, 2013**

**Synonyms.** None.

**Holotype.** Deposited in the Entomological Collection of the South African Museum (Iziko Museums of Cape Town), Cape Town, South Africa.

**Type locality.** South Africa, Western Cape, Cederberg Mountains, Algeria, Helskloof, 32°21.13'S, 19°03.57'E, ca 870 m ASL (see Remarks).

**Language of species description.** English.

**Remarks.** Altitude data obtained from Google Earth based on the given geographic coordinates. The species description includes morphological and molecular data.

**93. *Peripatopsisclavigera* Purcell, 1899**

**Synonyms.** None.

**Holotype.** Not designated (see Remarks). Neotype deposited in the Entomological Collection of the South African Museum (Iziko Museums of Cape Town), Cape Town, South Africa.

**Type locality.** South Africa, Western Cape, Knysna, Diepwalle Nature Reserve, 33°56.58'S, 23°08.67'E, ca 400 m ASL (see Remarks).

**Language of species description.** English.

**Remarks.** The holotype has not been designated explicitly in the original description. Specimens of *Peripatopsisclavigera* studied by Purcell (1899) had originally been deposited in the South African Museum of Natural History. Barnes et al. (2020: 583) designated a female collected at the Diepwalle Nature Reserve (Western Cape, South Africa) as neotype of *Peripatopsisclavigera*, but the authors did not clarify whether or not the syntypes are still available. If so, the neotype designation would need to be reverted. assumed that the syntypes are no longer preserved in South African Museum of Natural History. The neotype is deposited in the latter institution (Barnes et al. 2020: 583). The species has previously been regarded as a species complex by Daniels et al. (2009) and subsequently split into different species using morphological and molecular methods (Barnes et al. 2020). Molecular data obtained from a specimen collected at the Homtini River (Western Cape, South Africa) and initially assigned to *Peripatopsisclavigera* by Daniels et al. (2013) rather correspond to *Peripatopsismira* Barnes, Reiss & Daniels, 2020 (see *Peripatopsismira* below). Additional molecular data assigned to *Peripatopsisclavigera* (e.g., Murienne et al. 2014; Giribet et al. 2018) may not strictly correspond to the original species, as the sequenced specimens were collected 53 km of the type locality.

**94. *Peripatopsisedenensis* Barnes, Reiss & Daniels, 2020**

**Synonyms.** None.

**Holotype.** Deposited in the Entomological Collection of the South African Museum (Iziko Museums of Cape Town), Cape Town, South Africa.

**Type locality.** South Africa, Western Cape, Plettenberg Bay, Harkerville Forest, Garden of Eden Nature Reserve, 34°01.58'S, 23°11.52'E, ca 315 m ASL.

**Language of species description.** English.

**Remarks.** The species description includes morphological and molecular data. *Peripatopsisedenensis* has been described based on specimens from different localities lying up to 36 km away from the type locality. Given the disconnected haplotype network within this species (Barnes et al. 2020: 578), as well as the presence of distinct monophyletic units within this clade (Barnes et al. 2020: 575), the existence of a species complex within *Peripatopsisedenensis* cannot be excluded. For the sake of caution, the name *Peripatopsisedenensis* should only be applied to specimens from the type locality. Requires revision.

**95. *Peripatopsisferox* Barnes, Reiss & Daniels, 2020**

**Synonyms.** None.

**Holotype.** Deposited in the Entomological Collection of the South African Museum (Iziko Museums of Cape Town), Cape Town, South Africa.

**Type locality.** South Africa, Western Cape, Wilderness, Wilderness Nature Reserve, Half-Collared Kingfisher Trail, 33°59.14'S, 22°36.42'E, ca 55 m ASL.

**Language of species description.** English.

**Remarks.** The species description includes morphological and molecular data. *Peripatopsisferox* has been described based on specimens from many different localities lying up to 51 km away from the type locality. Given the disconnected haplotype network within this species (Barnes et al. 2020: 578), as well as the presence of distinct monophyletic units within this clade (Barnes et al. 2020: 575), the existence of a species complex within *Peripatopsisferox* cannot be excluded. For the sake of caution, the name *Peripatopsisferox* should only be applied to specimens from the type locality. Requires revision.

**96. *Peripatopsishamerae* Ruhberg & Daniels, 2013**

**Synonyms.** None.

**Holotype.** Deposited in the Entomological Collection of the South African Museum (Iziko Museums of Cape Town), Cape Town, South Africa.

**Type locality.** South Africa, Eastern Cape, Kamala Game Reserve, Somerset East area, Groot Bruintjieshoogte [Rietfontein], 32°36.30'S, 25°20.57'E, ca 1,265 m ASL (see Remarks).

**Language of species description.** English.

**Remarks.** Altitude data obtained from Google Earth based on the given geographic coordinates. The species description includes morphological and molecular data.

**97. *Peripatopsisjanni* Ruhberg & Daniels, 2013**

**Synonyms.** None.

**Holotype.** Deposited in the Entomological Collection of the South African Museum (Iziko Museums of Cape Town), Cape Town, South Africa.

**Type locality.** South Africa, Eastern Cape, Amathole Mountains, Kologha Forest, 32°40.67'S, 27°15'E, ca 1,230 m ASL (see Remarks).

**Language of species description.** English.

**Remarks.** Altitude data obtained from Google Earth based on the given geographic coordinates. The species description includes morphological and molecular data. *Peripatopsisjanni* has been described based on specimens from two different localities lying 31 km away from each other. Hence, it is unclear whether *Peripatopsisjanni* may represent a species complex. Requires closer revision.

**98. *Peripatopsislawrencei* McDonald, Ruhberg & Daniels, 2012**

**Synonyms.** None.

**Holotype.** Deposited in the Entomological Collection of the South African Museum (Iziko Museums of Cape Town), Cape Town, South Africa.

**Type locality.** South Africa, Western Cape, Riviersonderend, Oubos, 34°04.57'S, 19°49.73'E, ca 360 m ASL (see Remarks).

**Language of species description.** English.

**Remarks.** Altitude data obtained from Google Earth based on the given geographic coordinates. The species description includes morphological and molecular data. *Peripatopsislawrencei* has been described based on specimens from different localities lying up to more than 122 km away from the type locality. Given the presence of distinct monophyletic clades within *Peripatopsislawrencei* (McDonald et al. 2012: 59), the existence of a species complex within *Peripatopsislawrencei* cannot be excluded. For the sake of caution, the name *Peripatopsislawrencei* should only be applied to specimens from the type locality. Requires revision.

**99. *Peripatopsisleonina* Purcell, 1899**

**Synonyms.** None.

**Holotype.** Not designated (see Remarks).

**Type locality.** South Africa, Cape Town, Cape town side of Signal Hill (Lions Hill).

**Language of species description.** English.

**Remarks.** The holotype has not been designated explicitly in the original description. Specimens of *Peripatopsisleonina* studied by Purcell (1899) had originally been deposited in the South African Museum of Natural History. Whether or not these syntypes are still preserved at this institution could not be verified. Weidner (1959: 93) and Ruhberg (1985: 98) refer to ‘paratypoids’ and ‘ex types’ deposited in the Zoologisches Museum, Hamburg, Germany, the type-status of which is unclear. *Peripatopsisleonina* has not been recorded from natural habitats since 1912 (specimen NHM-1936.4.28.4 deposited in the Natural History Museum of London, UK), suggesting that the species is either extinct or critically endangered (Brinck 1957: 13; Ruhberg 1985: 98; Daniels et al. 2009: 201). Although this species requires morphological revision and molecular characterization, it may be difficult to accomplish due to the rarity of specimens and the critically endangered (CR) status of the species (Hamer 2003).

**100. *Peripatopsismellaria* Barnes, Reiss & Daniels, 2020**

**Synonyms.** None.

**Holotype.** Deposited in the Entomological Collection of the South African Museum (Iziko Museums of Cape Town), Cape Town, South Africa.

**Type locality.** South Africa, Western Cape, George, Witfontein Nature Reserve, 33°56.14'S, 22°26.09'E, ca 280 m ASL.

**Language of species description.** English.

**Remarks.** The species description includes morphological and molecular data. *Peripatopsismellaria* has been described based on specimens from different localities lying up to 45 km away from the type locality. Given the disconnected haplotype network within this species (Barnes et al. 2020: 578), as well as the presence of distinct monophyletic units within this clade (Barnes et al. 2020: 575), the existence of a species complex within *Peripatopsismellaria* cannot be excluded. For the sake of caution, the name *Peripatopsismellaria* should only be applied to specimens from the type locality. Requires revision.

**101. *Peripatopsismira* Barnes, Reiss & Daniels, 2020**

**Synonyms.** None.

**Holotype.** Deposited in the Entomological Collection of the South African Museum (Iziko Museums of Cape Town), Cape Town, South Africa.

**Type locality.** South Africa, Western Cape, Rheenendal, Homtini River, 33°56.51'S, 22°55.12'E, ca 235 m ASL.

**Language of species description.** English.

**Remarks.** The species description includes morphological and molecular data. *Peripatopsismira* has been described based on specimens from different localities lying up to 61 km away from the type locality. Given the disconnected haplotype network within this species (Barnes et al. 2020: 578), the existence of a species complex within *Peripatopsismira* cannot be excluded. For the sake of caution, the name *Peripatopsismira* should only be applied to specimens from the type locality. Requires revision.

**102. *Peripatopsismoseleyi* (Wood-Mason, 1879)**

**Synonyms.***Peripatusmoseleyi*, as originally described (Wood-Mason 1879: 155); *Peripatopsismoseleyi* (Purcell 1899: 338) (see Remarks).

**Holotype.** Not designated (see Remarks). Neotype deposited in the Entomological Collection of the South African Museum (Iziko Museums of Cape Town), Cape Town, South Africa.

**Type locality.** South Africa, Eastern Cape, Pirie Forest near King William’s town, 32°44.64'S, 27°17'E, ca 650 m ASL (see Remarks).

**Language of species description.** English.

**Remarks.** Species name suggested in a footnote in the original paper (Wood-Mason 1879: 155). The holotype has not been designated explicitly in the original description. Ruhberg and Daniels (2013: 139) designated a male collected at the Pirie Forest (Eastern Cape, South Africa) as neotype, which has been deposited in the South African Museum (Iziko Museums of Cape Town), Cape Town, South Africa. This species has previously been identified as a species complex (Daniels et al. 2009; Daniels and Ruhberg 2010) and has been subsequently revised by Ruhberg and Daniels (2013). Yet, *Peripatopsismoseleyi* sensu Ruhberg and Daniels (2013: 132; Clade ‘3’) still encloses specimens from different localities lying up to 53 km away from the type locality. Hence, the existence of a species complex within *Peripatopsismoseleyi* still cannot be excluded. For the sake of caution, the name *Peripatopsismoseleyi* should only be applied to specimens from the type locality. Requires revision.

**103. *Peripatopsisoverbergiensis* McDonald, Ruhberg & Daniels, 2012**

**Synonyms.** None.

**Holotype.** Deposited in the Entomological Collection of the South African Museum (Iziko Museums of Cape Town), Cape Town, South Africa.

**Type locality.** South Africa, Western Cape, Langeberg, Grootvadersbosch Nature Reserve, 33°58.92'S, 20°49.38'E, ca 415 m ASL (see Remarks).

**Language of species description.** English.

**Remarks.** Altitude data obtained from Google Earth based on the given geographic coordinates. Transcriptome data have recently been provided for this species (Sharma et al. 2014; Laumer et al. 2019; Baker et al. 2021). The species description includes morphological and molecular data. *Peripatopsisoverbergiensis* has been described based on specimens from different localities lying up to 215 km away from the type locality. Given the disconnected haplotype network within this species (McDonald and Daniels 2012: 830), as well as the presence of distinct monophyletic units within this clade (McDonald et al. 2012: 59), the existence of a species complex within *Peripatopsisoverbergiensis* cannot be excluded. For the sake of caution, the name *Peripatopsisoverbergiensis* should only be applied to specimens from the type locality. Requires revision.

**104. *Peripatopsispolychroma* Grobler, Myburgh, Barnes & Daniels, 2023**

**Synonyms.** None.

**Holotype.** Deposited in the Entomological Collection South African Museum of Natural History, Iziko Museum of Cape Town, Cape Town, South Africa.

**Type locality.** South Africa, KwaZulu-Natal, northern Drakensberg, Kamberg Nature Reserve (Mount Lebanon), 29°18.725'S, 29°41.220'E, ca 1,585 m ASL.

Language of species description: English.

**Remarks.** The species description includes morphological and molecular data. *Peripatopsispolychroma* has been described based on specimens from many different localities lying up to 136 km away from the type locality. Given the disconnected haplotype network within this species (Grobler et al. 2023: 8), as well as the presence of distinct monophyletic units within this clade (Grobler et al. 2023: 7), the existence of a species complex within *Peripatopsispolychroma* cannot be excluded. For the sake of caution, the name *Peripatopsispolychroma* should only be applied to specimens from the type locality. Geographic coordinates presented by Grobler et al. (2023) may contain a typo (DDM values presented in DMS format without proper conversion). Requires revision.

**105. *Peripatopsispurpureus* Daniels, McDonald & Picker, 2013**

**Synonyms.** None.

**Holotype.** Deposited in the Entomological Collection of the South African Museum (Iziko Museums of Cape Town), Cape Town, South Africa.

**Type locality.** South Africa, Western Cape, Limietberg, Du Toit’s Kloof, 33°42.72'S, 19°06.55'E, ca 760 m ASL (see Remarks).

**Language of species description.** English.

**Remarks.** Altitude data obtained from Google Earth based on the given geographic coordinates. The species description includes morphological and molecular data. *Peripatopsispurpureus* has been described based on specimens from different localities lying up to 36 km away from the type locality. Given the presence of distinct monophyletic units within this clade (Daniels et al. 2013: 662), the existence of a species complex within *Peripatopsispurpureus* cannot be excluded. For the sake of caution, the name *Peripatopsispurpureus* should only be applied to specimens from the type locality. Requires revision.

**106. *Peripatopsissedgwicki* Purcell, 1899**

**Synonyms.***Peripatusdewaali* Weber, 1898 (senior synonym, nomen oblitum; Weber 1898: 8); *Peripatopsissedgwicki* Purcell, 1899 (junior synonym, nomen protectum; Purcell 1899: 345) (see Remarks).

**Holotype.** Not designated (see Remarks).

**Type locality.** South Africa, Western Cape, most likely the environs of Knysna (Weber 1898: 8) (see Remarks).

**Language of species description.** Dutch.

**Remarks.** Note that the abbreviation *P.sedgwicki* may create confusion with the peripatid species *Peripatussedgwicki* (see Röhlig et al. 2010: 230; Oliveira et al. 2012a: 53). Oliveira et al. (2012a: 53) regarded the name *Peripatopsissedgwicki* Purcell, 1899 as nomen protectum and the senior synonym *Peripatusdewaali* Weber, 1898 (Weber, 1898: 8) as nomen oblitum, following the commandments of the ICZN (Art. 23.9.1). The holotype has not been designated explicitly in the original description. Specimens of *Peripatopsissedgwicki* studied by Purcell (1899) had originally been deposited in the South African Museum of Natural History. Whether or not these syntypes are still preserved at this institution could not be verified. Bouvier (1907a: 520) refers to a cotype [obsolete for syntype] deposited in the Museum National d’Histoire Naturelle de Paris, France (see also Le Bras et al. 2015). Molecular data including Expressed Sequence Tags (ESTs) available for putative specimens of *Peripatopsissedgwicki* (e.g., Meusemann et al. 2010; Daniels et al. 2009, 2017). However, *Peripatopsissedgwicki* is a species complex (Daniels et al. 2009, 2017), which requires morphological revision and molecular characterization.

**107. *Peripatopsisstelliporata* Sherbon & Walker, 2004**

**Synonyms.** None (see Remarks).

**Holotype.** Deposited in the Natural History Museum of London, UK.

**Type locality.** South Africa, Cape Town, Newlands Forest, 33°12'S, 18°24'E, ca 335 m ASL (see Remarks).

**Language of species description.** English.

**Remarks.** This species has recently been regarded as junior synonym of *Peripatopsisbalfouri* (Daniels et al. 2013: 669). However, *Peripatopsisstelliporata* is morphologically distinct from *Peripatopsisbalfouri* (Sherbon and Walker 2004: 304) besides forming a monophyletic subclade within the *Peripatopsisbalfouri* complex (Daniels et al. 2013: 663). Also, it occurs relatively far away from the type locality of *Peripatopsisbalfouri* (~ 38 km). Thus, their conspecificity is still inconclusive. For the sake of caution, and following the ICZN (Art. 23.3.6), I opted to regard *Peripatopsisstelliporata* as a valid species. Further revision is required. Altitude data obtained from Google Earth based on the given geographic coordinates.

**108. *Peripatopsisstorchi* Ruhberg & Daniels, 2013**

**Synonyms.** None.

**Holotype.** Deposited in the Entomological Collection of the South African Museum (Iziko Museums of Cape Town), Cape Town, South Africa.

**Type locality.** South Africa, Eastern Cape, Katberg, 32°28.22'S, 26°40.11'E, ca 1,045 m ASL.

**Language of species description.** English.

**Remarks.** The species description includes morphological and molecular data.

**109. *Peripatopsistulbaghensis* Barnes, Reiss & Daniels, 2020**

**Synonyms.** None.

**Holotype.** Deposited in the Entomological Collection of the South African Museum (Iziko Museums of Cape Town), Cape Town, South Africa.

**Type locality.** South Africa, Western Cape, Tulbagh, Groot Winterhoek, Secret Falls, 33°10.39'S, 19°07.56'E, ca 735 m ASL.

**Language of species description.** English.

**Remarks.** The species description includes morphological and molecular data.

**Nomina dubia**

**nd9. *Peripatopsisintermedia* Hutchinson, 1928**

**Synonyms.** None.

**Holotype.** Deposited in the Entomological Collection of the South African Museum (Iziko Museums of Cape Town), Cape Town, South Africa (see Remarks).

**Type locality.** South Africa, 11.26 km [7 miles] east of Montagu.

**Language of species description.** English.

**Remarks.** A holotype has been designated in the original description under the term ‘type’ (Hutchinson 1928: 338). The species has previously been regarded as a synonym of *Peripatopsisbalfouri* by Ruhberg (1985: 91). Oliveira et al. (2012a: 52) regarded the synonymization premature, as the only specimen known for this species (type) is in bad conditions (Ruhberg 1985: 91) and does not allow proper comparison with *Peripatopsisbalfouri* for unambiguously concluding about their synonymy. Although specimens of *Peripatopsisintermedia* have never been re-collected, the long distance between the type locality of *Peripatopsisbalfouri* and *Peripatopsisintermedia* (more than 170 km) lead Oliveira et al. (2012a: 52) to re-establish *Peripatopsisintermedia* as a valid species, which requires revision. Given these taxonomic uncertainties, and for the sake of cation, I opted for regarding *Peripatopsisintermedia* as a nomen dubium until further investigations are conducted.

##### XXXIII. *Phallocephale* Reid, 1996

**Type species.***Phallocephaletallagandensis* Reid, 1996, by monotypy (Reid 1996: 846).

**110. *Phallocephaletallagandensis* Reid, 1996**

**Synonyms.** None.

**Holotype.** Deposited in the Australian Museum, Sydney, Australia.

**Type locality.** Australia, New South Wales, Tallaganda State Forest, Forbes Creek Road, 35°28'S, 149°32'E, ca 1,000 m ASL.

**Language of species description.** English.

**Remarks.** The species description includes specimens from different localities lying up to 61 km away from the type locality, hence the existence of a species complex within *Phallocephaletallagandensis* cannot be excluded. For the sake of caution, the name *Phallocephaletallagandensis* should only be applied to specimens from the type locality. Molecular data available for this species may not strictly correspond to the original species, as the sequenced specimens were collected 41 km away from the type locality (Murienne et al. 2014; Giribet et al. 2018). Requires morphological revision and molecular characterization.

##### XXXIV. *Planipapillus* Reid, 1996

**Type species.***Planipapillustaylori* Reid, 1996, by original designation (Reid 1996: 851).

**Remarks.** Requires molecular characterization.

**111. *Planipapillusannae* Reid, 2000b**

**Synonyms.** None.

**Holotype.** Deposited in the Melbourne Museum [originally described as Museum Victoria], Melbourne, Australia.

**Type locality.** Australia, Victoria, 5.9 km northwest of Bonang, beside Deddick River Road (between Bonang and Tubbut), 37°11'S, 148°41'E, ca 740 m ASL.

**Language of species description.** English.

**Remarks.** Requires molecular characterization.

**112. *Planipapillusberti* Reid, 2000b**

**Synonyms.** None.

**Holotype.** Deposited in the Melbourne Museum [originally described as Museum Victoria], Melbourne, Australia.

**Type locality.** Australia, Victoria, Granite Flat, 9 km south of Mitta Mitta, beside Omeo Highway, north of intersection of Omeo Highway and Walsh’s Road, 36°35'S, 147°27'E, ca 350 m ASL.

**Language of species description.** English.

**Remarks.** Requires molecular characterization.

**113. *Planipapillusbiacinaces* Reid, 1996**

**Synonyms.** None.

**Holotype.** Deposited in the Melbourne Museum [originally described as Museum Victoria], Melbourne, Australia.

**Type locality.** Australia, Victoria, Howman Gap, 36°50'S, 147°16'E, ca 1,260 m ASL.

**Language of species description.** English.

**Remarks.** Requires morphological revision and molecular characterization.

**114. *Planipapillusbiacinoides* Reid, 2000b**

**Synonyms.** None.

**Holotype.** Deposited in the Melbourne Museum [originally described as Museum Victoria], Melbourne, Australia.

**Type locality.** Australia, Victoria, beside Livingtone Creek at intersection of Birregun Road and Upper Livingstone Track (6.2 km south of intersection of Cassilis Road and Birregun Road), 37°05'S, 147°36'E, ca 300 m ASL.

**Language of species description.** English.

**Remarks.** Requires morphological revision and molecular characterization.

**115. *Planipapillusbulgensis* Reid, 1996**

**Synonyms.** None.

**Holotype.** Deposited in the Melbourne Museum [originally described as Museum Victoria], Melbourne, Australia.

**Type locality.** Australia, Victoria, Tarra-Bulga National Park, 38°26'S, 146°32'E, ca 580 m ASL.

**Language of species description.** English.

**Remarks.** Requires molecular characterization.

**116. *Planipapilluscyclus* Reid, 2000b**

**Synonyms.** None.

**Holotype.** Deposited in the Melbourne Museum [originally described as Museum Victoria], Melbourne, Australia.

**Type locality.** Australia, Victoria, 9 km north of Club Terrace, junction of Errinundra Road and Combienbar Road, 37°28'S, 148°55'E, ca 130 m ASL.

**Language of species description.** English.

**Remarks.** Requires molecular characterization.

**117. *Planipapillusgracilis* Reid, 2000b**

**Synonyms.** None.

**Holotype.** Deposited in the Melbourne Museum [originally described as Museum Victoria], Melbourne, Australia.

**Type locality.** Australia, Victoria, beside Livingstone Creek, at intersection of Birregun Road and Upper Livingstone Track (6.2 km south of Cassilis Road and Birregun Road), 37°05'S, 147°36'E, ca 300 m ASL.

**Language of species description.** English.

**Remarks.** Requires molecular characterization.

**118. *Planipapillusimpacris* Reid, 2000b**

**Synonyms.** None.

**Holotype.** Deposited in the Melbourne Museum [originally described as Museum Victoria], Melbourne, Australia.

**Type locality.** Australia, New South Wales, South East Forests National Park, Coolangubra Section, 5 km north of intersection of Coolangubra Forest Way and Northern Access Road, 37°01'S, 149°23'E, ca 800 m ASL.

**Language of species description.** English.

**Remarks.** Requires molecular characterization.

**119. *Planipapillusmundus* Reid, 1996**

**Synonyms.** None.

**Holotype.** Deposited in the Australian Museum, Sydney, Australia.

**Type locality.** Australia, New South Wales, Wilsons Valley, 36°21'S, 148°32'E, ca 1,360 m ASL.

**Language of species description.** English.

**Remarks.** Requires molecular characterization.

**120. *Planipapillustaylori* Reid, 1996**

**Synonyms.** None.

**Holotype.** Deposited in the Australian Museum, Sydney, Australia.

**Type locality.** Australia, New South Wales, Bombala River, 36°37'S, 149°22'E, ca 1,120 m ASL.

**Language of species description.** English.

**Remarks.** Requires molecular characterization.

**121. *Planipapillustectus* Reid, 2000b**

**Synonyms.** None.

**Holotype.** Deposited in the Melbourne Museum [originally described as Museum Victoria], Melbourne, Australia.

**Type locality.** Australia, Victoria, 6.7 km south of the intersection of Gelantipy Road and Tulloch Ard Road (10.7 km south of Gelantipy, 300 m north of Forest Creek Track), 37°17'S, 148°15'E, ca 710 m ASL.

**Language of species description.** English.

**Remarks.** Requires molecular characterization.

**122. *Planipapillusvittatus* Reid, 2000b**

**Synonyms.** None.

**Holotype.** Deposited in the Melbourne Museum [originally described as Museum Victoria], Melbourne, Australia.

**Type locality.** Australia, Victoria, Dinner Plain, 36°59'S, 147°17'E, ca 1,630 m ASL.

**Language of species description.** English.

**Remarks.** Requires revision, particularly at molecular level.

##### XXXV. *Regimitra* Reid, 1996

**Type species.***Regimitraquadricaula* Reid, 1996, by monotypy (Reid 1996: 863).

**Remarks.** Requires molecular characterization.

**123. *Regimitraquadricaula* Reid, 1996**

**Synonyms.** None.

**Holotype.** Deposited in the Australian Museum, Sydney, Australia.

**Type locality.** Australia, New South Wales, Tuggolo State Forest, 31°31'S, 151°27'E, ca 1,060 m ASL.

**Language of species description.** English.

**Remarks.** Requires molecular characterization

##### XXXVI. *Ruhbergia* Reid, 1996

**Type species.***Ruhbergiabifalcata* Reid, 1996, by original designation (Reid 1996: 868).

**Remarks.** Requires molecular characterization.

**124. *Ruhbergiabifalcata* Reid, 1996**

**Synonyms.** None.

**Holotype.** Deposited in the Australian Museum, Sydney, Australia.

**Type locality.** Australia, New South Wales, Tinderry Mountains, 35°40'S, 149°15'E, ca 1,300 m ASL.

**Language of species description.** English.

**Remarks.** Requires molecular characterization.

**125. *Ruhbergiabrevicorna* Reid, 1996**

**Synonyms.** None.

**Holotype.** Deposited in the Australian Museum, Sydney, Australia.

**Type locality.** Australia, New South Wales, Mount Fairy (northwestern Bungendore), 35°09'S, 149°33'E, ca 820 m ASL.

**Language of species description.** English.

**Remarks.** Requires molecular characterization.

**126. *Ruhbergiarostroides* Reid, 1996**

**Synonyms.** None.

**Holotype.** Deposited in the Australian Museum, Sydney, Australia.

**Type locality.** Australia, New South Wales, Wombeyan Caves, intersection of Wombeyan Caves Road and Langs Road, 34°18'S, 150°01'E, ca 420 m ASL.

**Language of species description.** English.

**Remarks.** Requires molecular characterization.

##### XXXVII. *Sphenoparme* Reid, 1996

**Type species.***Sphenoparmehobwensis* Reid, 1996, by monotypy (Reid 1996: 878).

**Remarks.** Requires molecular characterization.

**127. *Sphenoparmehobwensis* Reid, 1996**

**Synonyms.** None.

**Holotype.** Deposited in the Queensland Museum, Brisbane, Australia.

**Type locality.** Australia, Queensland, Lamington National Park, Mount Hobwee, 28°15'S, 153°14'E, ca 500 m ASL.

**Language of species description.** English.

**Remarks.** Requires molecular characterization.

##### XXXVIII. *Tasmanipatus* Ruhberg, Mesibov, Briscoe & Tait, 1991

**Type species.***Tasmanipatusbarretti* Ruhberg, Mesibov, Briscoe & Tait, 1991, by original designation (Ruhberg et al. 1991: 7).

**Remarks.** The genus originally contained two nominal species, namely *Tasmanipatusbarretti* and *Tasmanipatusanophthalmus* (Ruhberg, Mesibov, Briscoe & Tait, 1991). The latter, however, is now known under the name *Leucopatusanophthalmus* (Ruhberg, Mesibov, Briscoe & Tait, 1991) (see Oliveira et al. 2018).

**128. *Tasmanipatusbarretti* Ruhberg, Mesibov, Briscoe & Tait, 1991**

**Synonyms.** None.

**Holotype.** Deposited in the Queen Victoria Museum and Art Gallery, Launceston, Australia.

**Type locality.** Australia, Tasmania, Evercreech Rivulet, 41°27'S, 147°57'E, ca 310 m ASL (see Remarks).

**Language of species description.** English.

**Remarks.** Altitude data obtained from Google Earth based on the geographic coordinates provided by Oliveira et al. (2018). Species redescription includes morphological, molecular, karyotype and slime protein profiling data (Oliveira et al. 2018). Additional molecular data available for this species (e.g., Murienne et al. 2014; Giribet et al. 2018). According to Oliveira et al. (2018: 930) the existence of a species complex within *Tasmanipatusbarretti* cannot be excluded. For the sake of caution, the name *Tasmanipatusbarretti* should only be applied to specimens from the type locality. Geographic coordinates assigned to *Tasmanipatusbarretti* in Giribet at al. (2018) may contain a typo.

##### XXXIX. *Tetrameraden* Reid, 1996

**Type species.***Tetrameradenmeringos* Reid, 1996, by monotypy (Reid 1996: 886).

**Remarks.** Requires molecular characterization.

**129. *Tetrameradenmeringos* Reid, 1996**

**Synonyms.** None.

**Holotype.** Deposited in the Australian Museum, Sydney, Australia.

**Type locality.** Australia, New South Wales, Warrumbungle Range, Siding Springs Mountain, 31°16'S, 149°04'E, ca 1,165 m ASL.

**Language of species description.** English.

**Remarks.** Requires molecular characterization.

##### XL. *Vescerro* Reid, 1996

**Type species.***Vescerroturbinatus* Reid, 1996, by monotypy (Reid 1996: 890).

**Remarks.** Requires molecular characterization.

**130. *Vescerroturbinatus* Reid, 1996**

**Synonyms.** None.

**Holotype.** Deposited in the Queensland Museum, Brisbane, Australia.

**Type locality.** Australia, Queensland, Iron Range, Claudie River, 12°45'S, 143°14'E, ca 50 m.

**Language of species description.** English.

**Remarks.** The species description includes specimens from different localities lying up to 110 km away from the type locality, hence the existence of a species complex within *Vescerroturbinatus* cannot be excluded. For the sake of caution, the name *Vescerroturbinatus* should only be applied to specimens from the type locality. Requires morphological revision and molecular characterization.

##### XLI. *Wambalana* Reid, 1996

**Type species.***Wambalanamakrothele* Reid, 1996, by monotypy (Reid 1996: 894).

**Remarks.** Requires molecular characterization.

**131. *Wambalanamakrothele* Reid, 1996**

**Synonyms.** None.

**Holotype.** Deposited in the Australian Museum, Sydney, Australia.

**Type locality.** Australia, New South Wales, Telegherry State Forest, 32°07'S, 151°41'E, ca 900 m.

**Language of species description.** English.

**Remarks.** The species description includes specimens from different localities lying up to 33 km away from the type locality, hence the existence of a species complex within *Wambalanamakrothele* cannot be excluded. For the sake of caution, the name *Wambalanamakrothele* should only be applied to specimens from the type locality. Requires morphological revision and molecular characterization.

#### ﻿Fossil species with uncertain relationship to the extant taxa

##### I. †*Antennipatus* Garwood, Edgecombe & Giribet, 2016

**Higher classification.** Not available (see Remarks).

**Type species.** †*Antennipatusmontceauensis* Garwood, Edgecombe & Giribet, 2016, by monotypy (Garwood et al. 2016: 184).

**Remarks.** †*Antennipatus* has not been assigned to any high-ranking taxon within Onychophora (Garwood et al. 2016: 183).

**1. † *Antennipatusmontceauensis* Garwood, Edgecombe & Giribet, 2016**

**Synonyms.** None.

**Holotype.** Deposited in the Muséum d’Histoire Naturelle d’Autun, France [but belongs to the Museum National d’Histoire Naturelle de Paris, France].

**Type locality.** France, northeast of the Massif Central, Montceau-les-Mines Lagerstätte.

**Language of species description.** English.

**Remarks.** †*Antennipatusmontceauensis* is described from the Assise de Montceau (or Great Seams Formation) of the Montceau-les-Mines Lagerstätte (Garwood et al. 2016: 181). The material dates from the Carboniferous period, more precisely from the Late Pennsylvanian [Stephanian], 304–299 Ma. It remains unclear, whether this species represents a stem- or a crown-group onychophoran and, although it is plausible that it was a terrestrial organism (Garwood et al. 2016: 188), its relationship to extant onychophorans is unclear.

##### II. †*Cretoperipatus* Engel & Grimaldi, 2002

**Higher classification.**Peripatidae Burmeister, 1837 (see Remarks).

**Type species.** †*Cretoperipatusburmiticus* Engel & Grimaldi, 2002, by monotypy (Grimaldi et al. 2002: 24).

**Remarks.** A wrong authorship [Grimaldi, Engel & Nascimbene] has been assigned to †*Cretoperipatus* by Oliveira et al. (2012a: 58). The genus authorship [Engel & Grimaldi] differs from the authorship of the original publication (Grimaldi et al. 2002). †*Cretoperipatus* is currently classified within Peripatidae (Grimaldi et al. 2002: 24; Oliveira et al. 2016: 2594).

**2. † *Cretoperipatusburmiticus* Engel & Grimaldi, 2002**

**Synonyms.** None.

**Holotype.** Deposited in the American Museum of Natural History, New York, USA.

**Type locality.** Myanmar, Kachin, Tanai [Danai] Village, Ledo Road, 105 km northwest of Myitkyina.

**Language of species description.** English.

**Remarks.** A wrong authorship [Grimaldi et al.] has been assigned to †*Cretoperipatusburmiticus* by Oliveira et al. (2012a: 58). The species authorship [Engel & Grimaldi] differs from the authorship of the original publication (Grimaldi et al. 2002). The species name has been misspelt †*Cretozperipatusburmiticus* [sic] by Oliveira et al. (2012a: 58). The original species description was based on an incomplete specimen preserved in Burmese amber. The material dates from the Cretaceous period, more precisely from the earliest Cenomanian, ~ 100 Ma (Oliveira et al. 2016: 2594). The species has recently been redescribed based on new material and synchrotron radiation-based X-ray microtomography (SRµCT) data (Oliveira et al. 2016). †*Cretoperipatusburmiticus* is currently interpreted as crown-group Peripatidae, more specifically as one of the closest relatives of the Southeast Asian taxa *Typhloperipatus* and *Eoperipatus* (Oliveira et al. 2016: 2599).

##### III. †*Helenodora* Thompson & Jones, 1980

**Higher classification.** Not available (see Remarks).

**Type species.** †*Helenodorainopinata* Thompson & Jones, 1980, by monotypy (Thompson and Jones 1980: 588).

**Remarks.** †*Helenodora* has previously been designated as a subjective synonym of †*Ilyodes* Scudder, 1890 (see Pacaud et al. 1981: 38), even though the latter is a fossil taxon originally assigned to Myriapoda (Scudder 1890: 422). Re-examination of type material, however, led Murdock et al. (2016: 2) to revert the synonymy and reinstate †*Helenodora* as a valid name (see also Garwood et al. 2016: 186). †*Helenodora* has not been assigned to any high-ranking taxon within Onychophora (Thompson and Jones 1980: 588; Murdock et al. 2016: 2).

**3. † *Helenodorainopinata* Thompson & Jones, 1980**

**Synonyms.** †*Ilyodesinopinata* (Haug et al. 2012: 1673) (see Remarks).

**Holotype.** Deposited in the Field Museum of Natural History, Chicago, Illinois, USA.

**Type locality.** United States of America, Northern Illinois, Mazon Creek fossil deposit, Will and Kankakee counties, Carbondale Formation, Francis Creek Shale.

**Language of species description.** English.

**Remarks.** †*Helenodorainopinata* is unlikely to be conspecific with either †*Ilyodeselongata* Scudder, 1890 or †*Ilyodesdivisa* Scudder, 1890 (Murdock et al. 2016: 2). The combination †*Ilyodesinopinata* used by Haug et al. (2012: 1673) followed previous synonymization (Pacaud et al. 1981: 38; Rolfe et al. 1982: 427; see also supplementary discussion in Haug et al. (2012). Nevertheless, this species has subsequently been revised by Murdock et al. (2016) and the combination †*Helenodorainopinata* has been reinstated (Murdock et al. 2016: 2). The material dates from the Carboniferous period, more precisely from the Middle Pennsylvanian [Westphalian D], ~ 309 Ma. The species was tentatively assigned to crown-group Onychophora in the original description (Thompson and Jones 1980: 596). However, more recent studies rather regarded †*Helenodorainopinata* as a stem-group onychophoran (Murdock et al. 2016: 11; Garwood et al. 2016: 180).

##### IV. †*Succinipatopsis* Poinar, 2000

**Higher classification.** (Ontonychophora (Tertiapatoidea (Succinipatopsidae))) Poinar, 2000 (see Remarks).

**Type species.** †*Succinipatopsisbalticus* Poinar, 2000, by monotypy (Poinar 2000: 107).

**Remarks.** The assignment of †*Succinipatopsis* to Onychophora should be regarded as incertae sedis (see Garwood et al. 2016: 180). Higher-ranking taxa raised by Poinar (2000: 105) to accommodate †*Succinipatopsis* should not be regarded as valid taxonomic categories within Onychophora (Grimaldi et al. 2002: 25).

**4. † *Succinipatopsisbalticus* Poinar, 2000**

**Synonyms.** None.

**Holotype.** Deposited in the Oregon State University, Corvallis, USA.

**Type locality.** Baltic Region.

**Language of species description.** English.

**Remarks.** A morphological account on the only specimen known has been published four years before the formal species description (Poinar 1996). The material preserved in Baltic amber dates from the middle Paleogene period, more precisely from the Lower Eocene, ~ 40 Ma. Although originally interpreted as crown-group onychophoran, †*Succinipatopsisbalticus* neither exhibits the characteristic onychophoran cuticle nor any other diagnostic characters of the group (Garwood et al. 2016: 180). Thus, its assignment to Onychophora must be regarded as incertae sedis. Furthermore, higher-ranking taxa raised by Poinar (2000: 105) to accommodate †*Succinipatopsisbalticus* are currently not regarded as valid taxonomic categories within Onychophora (Grimaldi et al. 2002: 25). Requires revision.

##### V. †*Tertiapatus* Poinar, 2000

**Higher classification.** (Ontonychophora (Tertiapatoidea (Tertiapatidae))) Poinar, 2000 (see Remarks).

**Type species.** †*Tertiapatusdominicanus* Poinar, 2000, by monotypy (Poinar 2000: 105).

**Remarks.** The assignment of †*Tertiapatus* to Onychophora should be regarded as incertae sedis (see Garwood et al. 2016: 180). Higher-ranking taxa raised by Poinar (2000: 105) to accommodate †*Tertiapatus* should not be regarded as valid taxonomic categories within Onychophora (Grimaldi et al. 2002: 25).

**5. † *Tertiapatusdominicanus* Poinar, 2000**

**Synonyms.** None.

**Holotype.** Deposited in the Oregon State University, Corvallis, USA.

**Type locality.** Dominican Republic.

**Language of species description.** English.

**Remarks.** A morphological account on the only specimen known has been published four years before the formal species description (Poinar 1996). The material preserved in Dominican amber is assigned with imprecise age ranging from the from the middle Paleogene (Eocene; ~ 45 Ma) to the middle Neogene (Miocene; ~ 15 Ma) periods. Although originally interpreted as crown-group onychophoran, †*Tertiapatusdominicanus* exhibits arthropodized antennae (Garwood et al. 2016: 180), hence its assignment to Onychophora must be regarded as incertae sedis. Furthermore, higher-ranking taxa raised by Poinar (2000: 105) to accommodate †*Tertiapatusdominicanus* are currently not regarded as valid taxonomic categories within Onychophora (Grimaldi et al. 2002: 25). Requires revision.

**Closing date of checklist data**: 18 August 2023.

## ﻿Discussion

An updated version of the first and only annotated checklist available for Onychophora (Oliveira et al. 2012a) was long overdue, not only because of nomenclatural acts suggested during the last decade, but also due to the recent flourish of molecular data availability within the group, which progressively changed the traditional way of identifying and assessing onychophoran species. Herein, the content of the previous checklist (Oliveira et al. 2012a) has been revisited and complemented to better meet the current needs and standards in onychophorology, for example, by bringing more comprehensive taxonomic information on each taxon. This includes remarks on the accuracy of collecting data, location of type material other than the holotype, and most importantly, the availability of molecular data useful for species identification. Some of these new aspects are discussed in detail below. Furthermore, relevant historical data tardily found in old literature, e.g., the correct authorship/year for Onychophora Grube, 1850 and Peripatidae Audouin & Milne-Edwards, 1832, as well as amendments suggested by international colleagues on locality and institutions names, taxon authority, collecting data, and typos, have now been incorporated into the revised checklist.

According to the data, 237 species are currently described for Onychophora, with 140 assigned to Peripatopsidae, 92 to Peripatidae, and five to the list of fossil species with unclear relationships to extant taxa (Table 1, Suppl. materials 1, 2). Among them, 216 species may be regarded as valid, whereas the remaining 21 represent nomina dubia, i.e., available yet doubtful names, the identity of which cannot be resolved unambiguously due to major taxonomical inconsistencies (Table 1, Suppl. materials 1, 2). At first glance, the number of nomina dubia presented here does not deviate much from that of Oliveira et al. (2012a) (see Table 1, Suppl. materials 1, 2). However, several changes in species status were necessary, as relevant data and/or details of the ICZN had previously been overlooked by Oliveira et al. (2012a). A reassessment of species status in the light of new data led previous nomina dubia to be interpreted as valid species within *Ooperipatellus* and *Peripatoides*, and vice-versa within *Epiperipatus*, *Macroperipatus*, *Opisthopatus*, and *Peripatopsis*.

**Table 1.** Number of species and genera currently described for Onychophora. Previous data from Oliveira et al. (2012a) are provided for comparison purposes.

	Oliveira et al. (2012a)	present study	relative increase (%)
**Onychophora (total)**			
valid genera	52	59	13.5
valid species	180	216	20.0
nomina dubia	20	21	5.0
total species number	200	237	18.5
**Peripatidae**			
valid genera	10	13	30
valid species	73	80	9.6
nomina dubia	9	12	33.3
total species number	82	92	12.2
**Peripatopsidae**			
valid genera	39	41	5.1
valid species	104	131	26.0
nomina dubia	11	9	-18.2
total species number	115	140	21.7
**Fossil**			
valid genera	3	5	66.7
valid species	3	5	66.7
nomina dubia	0	0	0
total species number	3	5	66.7

**Figure 2.**
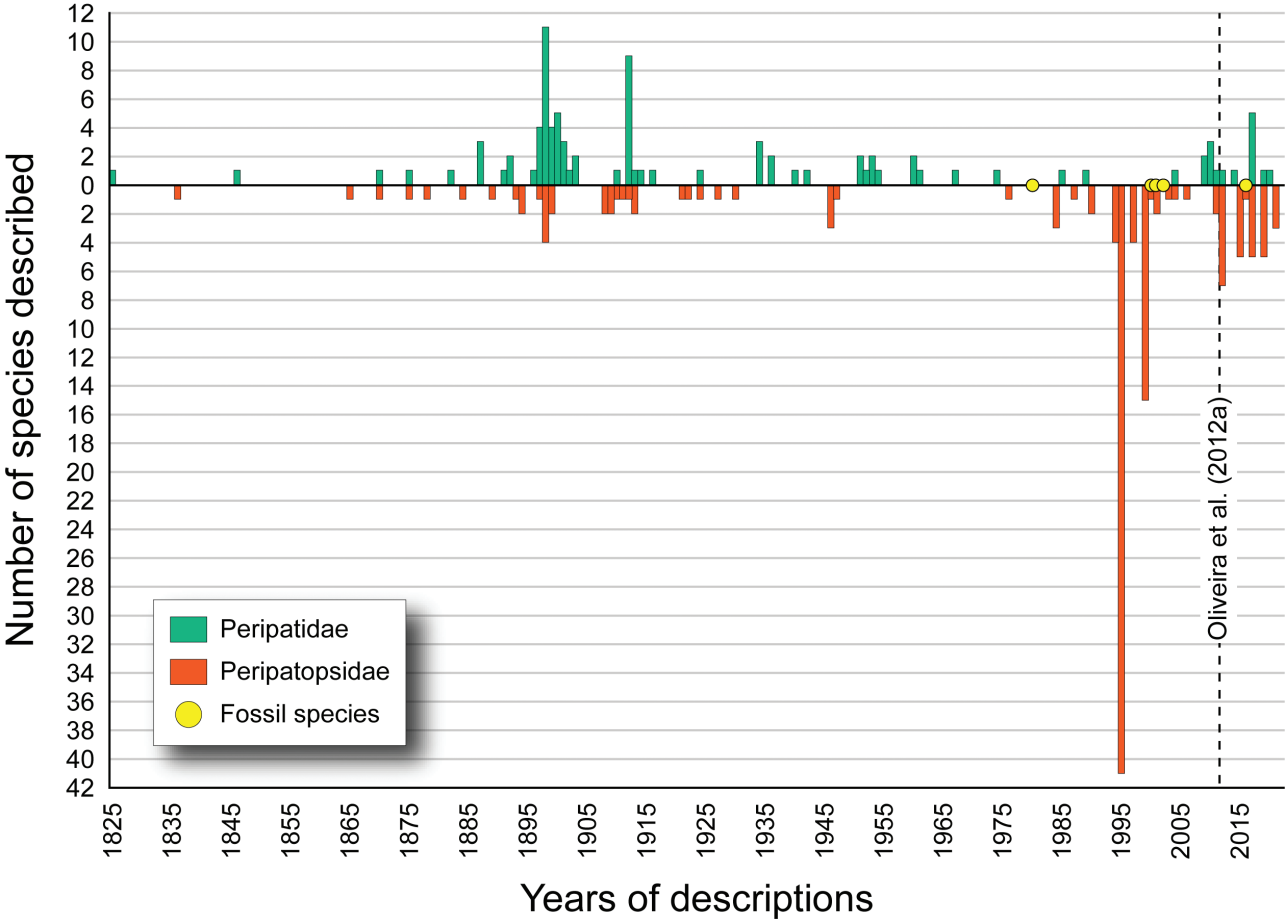
Number of onychophoran species described per year since the first description by Guilding (1826). Sparse descriptions of fossil taxa are indicated by yellow dots on the x-axis. Note the gaps in the taxonomy literature of Onychophora.

Since the previous checklist (Oliveira et al. 2012a), 37 species have been added to Onychophora. Although this represents an increase of 18.5% in the total diversity described for the group (see Table 1), taxonomic descriptions within Onychophora are still slow-paced, with an average of 3.6 species being described every year since 2012. The newly added species include 25 peripatopsids, ten peripatids, and two fossils, one of which was introduced more than four decades ago, †*Helenodorainopinata* Thompson & Jones, 1980, but was only recently unambiguously resolved within Onychophora (Murdock et al. 2016) (Fig. 2). As in the past, most taxonomic studies of the last decade have been conducted on Peripatopsidae (Fig. 2), whereas Peripatidae remains as the least studied onychophoran subgroup (Oliveira et al. 2012a). In this regard, many efforts have recently been made to improve the taxonomic scenario within this subgroup, with potential taxonomically informative features being explored at different methodological bases (Jeffery et al. 2012; Oliveira et al. 2012a, 2013, 2014, 2015; Baer et al. 2014; Chagas-Júnior and Costa 2014; Costa and Giribet 2016, 2021; Costa et al. 2018, 2021; Barquero-González et al. 2020). These studies, however, suggest that Peripatidae is taxonomically challenging at both morphological (Oliveira et al. 2012b, 2013, 2015) and molecular grounds (Giribet et al. 2018; Costa et al. 2021; Baker et al. 2021). Peripatopsidae, on the other hand, is comparatively more tractable from the taxonomic standpoint and it is somewhat expected more species to be described within this subgroup. Regardless the subgroup, the necessity of focused taxonomic works within Onychophora is very evident.

In recent years, the taxonomic criteria used for species delimitation and the characters used for species diagnoses in Onychophora have undergone a shift from a purely morphological basis (e.g., Ruhberg 1985; Read 1988b; Reid 1996; Oliveira et al. 2010), to a multimethodological approach, with molecular methods rapidly gaining ground as a standard procedure among taxonomists (e.g., Oliveira et al. 2015, 2018; Barnes et al. 2020; Costa and Giribet 2021). Consequently, numerous species have been described mainly (if not exclusively) based on molecular data (e.g., Trewick 1998; Daniels et al. 2016; Sato et al. 2018) and a great number of molecular sequences has become available for previously known taxa (e.g., Murienne et al. 2014; Giribet et al. 2018; Costa et al. 2021). Since molecular data arguably facilitate species identification, assessment, and revision, it seemed appropriated to point out their availability for each taxon herein. While compiling these data, I have identified an inconspicuous yet concerning issue: many molecular sequences have been assigned with species names, even though they have been obtained from specimens collected far-off the type locality of the respective species. Given the recent evidence that nearly all onychophoran species are endemic and restricted to very limited areas (Lacorte et al. 2011a, b; Oliveira et al. 2011, 2012b, 2015, 2018; Bull et al. 2013; Bull and Sunnucks 2014; Daniels et al. 2016; Costa et al. 2018; Barnes and Daniels 2019, 2022; Barnes et al. 2020); however, it is very likely that those sequenced specimens do not strictly correspond to the original species.

Such cases proved to be recurrent in the literature and, although authors have undoubtedly been well-intentioned, the obtained sequences are now publicly available in online databases (e.g., GenBank) and have continuously been used in other studies under the suggested species name, without further taxonomic verification. In other words, taxonomic inaccuracy is propagated unintentionally and at very fast rates within Onychophora. A prudent way of managing the current situation would be, for example, only assigning with a species name sequences obtained from type material and/or topotypes matching the original species description. If additional material has been analyzed instead, one could simply suggest an open nomenclature for the sequenced material, e.g., using the Latin terms *affinis* (aff.; related to) or *confer* (cf.; requires verification), thus avoiding dissemination of misidentified data (e.g., Giribet et al. 2018).

Finally, I have also observed that several species have originally been described based on material from multiple localities lying several kilometers from each other. Assuming that conspecificity becomes less likely as the distance between collecting sites increases (Oliveira et al. 2011), and that onychophorans are morphologically very uniform (e.g., Bouvier 1905, 1907b; Read 1988a), those species might rather represent complexes of two or more taxa being treated under the same name. From a taxonomic perspective, species complexes are per se not problematic, but they arguably mask the real species diversity of Onychophora and should thus be target of more detailed investigations (e.g., Barnes and Daniels 2019). This proved to be the case of different South African peripatopsids, which have recently been reassessed and split into several species (e.g., Daniels et al. 2013, 2016; Ruhberg and Daniels 2013; Barnes et al. 2020). While geographical information found in the literature suggests that species complexes may be more widespread among onychophorans than previously thought, assessing these data turned out puzzling. For instance, some of the locality names found along the species descriptions could not be verified herein, as they seem to be either out-of-date or incorrect. To further complicate matters, numerous geographic coordinates provided for collected material seem inaccurate, as they either do not match the position of the given locality name, or contain typos, or correspond to areas where onychophorans are unlikely occur, such as in the middle of water masses. These and the other issues discussed above have thus been pointed out in the present checklist for facilitating and encouraging future investigations. In addition, an overview of species names and the taxonomic classification within Onychophora can be accessed on the Onychophora Website (Oliveira et al. 2023), which has regularly been updated and may be considered as additional source of information on this charismatic yet understudied animal group.

## ﻿Additional information

### Author ORCIDs

Ivo de Sena Oliveira https://orcid.org/0000-0002-8340-7660

### Data availability

All of the data that support the findings of this study are available in the main text or Supplementary Information.

## Supplementary materials

Supplementary material 1 Number of valid species and nomina dubia currently assigned to each genus of Onychophora Data type xlsx Explanation note Previous data from Oliveira et al. (2012a) are provided for comparison purposes. https://binary.pensoft.net/file/937804This dataset is made available under the Open Database License (http://opendatacommons.org/licenses/odbl/1.0/). The Open Database License (ODbL) is a license agreement intended to allow users to freely share, modify, and use this Dataset while maintaining this same freedom for others, provided that the original source and author(s) are credited.Author: Ivo de Sena Oliveira

Supplementary material 2 Summary of all species and genera currently assigned to Onychophora Data type xlsx Explanation note The type species of each genus is presented in red. ^1^‘Péripates Andicoles’ sensu Bouvier (1899a: 1030) currently corresponds to the genus *Oroperipatus*. https://binary.pensoft.net/file/937805This dataset is made available under the Open Database License (http://opendatacommons.org/licenses/odbl/1.0/). The Open Database License (ODbL) is a license agreement intended to allow users to freely share, modify, and use this Dataset while maintaining this same freedom for others, provided that the original source and author(s) are credited.Author: Ivo de Sena Oliveira
